# Amalgamated cross-species transcriptomes reveal organ-specific propensity in gene expression evolution

**DOI:** 10.1038/s41467-020-18090-8

**Published:** 2020-09-08

**Authors:** Kenji Fukushima, David D. Pollock

**Affiliations:** 1 Department of Biochemistry and Molecular Genetics, University of Colorado School of Medicine, Aurora, CO 80045 USA; 2 Present Address: Institute for Molecular Plant Physiology and Biophysics, University of Würzburg, Würzburg, Germany

**Keywords:** Gene regulatory networks, Molecular evolution, Evolutionary biology, Gene expression

## Abstract

The origins of multicellular physiology are tied to evolution of gene expression. Genes can shift expression as organisms evolve, but how ancestral expression influences altered descendant expression is not well understood. To examine this, we amalgamate 1,903 RNA-seq datasets from 182 research projects, including 6 organs in 21 vertebrate species. Quality control eliminates project-specific biases, and expression shifts are reconstructed using gene-family-wise phylogenetic Ornstein–Uhlenbeck models. Expression shifts following gene duplication result in more drastic changes in expression properties than shifts without gene duplication. The expression properties are tightly coupled with protein evolutionary rate, depending on whether and how gene duplication occurred. Fluxes in expression patterns among organs are nonrandom, forming modular connections that are reshaped by gene duplication. Thus, if expression shifts, ancestral expression in some organs induces a strong propensity for expression in particular organs in descendants. Regardless of whether the shifts are adaptive or not, this supports a major role for what might be termed preadaptive pathways of gene expression evolution.

## Abstract (web summary)

Multicellularity requires complex coordinated gene expression. Fukushima and Pollock find that gene expression in different organs is likely to constrain future patterns of gene expression evolution, particularly following gene duplication.

## Introduction

Vertebrate organs organize physiological activities, and the diverse expression patterns of thousands of genes determines organ identities and functions. Because of this, the evolution of gene expression patterns plays a central role in organismal evolution. The degree of organ expression specificity correlates to how fast amino acids substitute^1^, how rapidly they change expression levels^2^, and patterns of histone modifications^3^. Major organ-altering evolutionary events such as development of the hominoid brain are also associated with gene expression shifts^4–7^. However, although gene duplication is well-known to play an important role in expression pattern shifts (see e.g., the ortholog conjecture^8–11^), the evolutionary dynamics of expression patterns with and without gene duplication remain poorly understood. An important question is whether long-term expression in one organ predisposes genes to be subsequently utilized in other organs.

A possible theoretical basis for such predisposition is the idea that certain preexisting adapted states are more conducive to evolution of specific new traits than other preexisting states. This is known as preadaptation, and when a trait makes such a shift it is referred to as exaptation^12^. Evidence for preadaptation was long ago found in phenotypic traits^13^, and recently in molecular traits such as protein sequences during de novo gene birth^14^ or during functional innovations^15^. Protein sequence evolution generally involves highly epistatic interactions and context-dependent changes^15,16^ that affect preadaptation, but the modular nature of expression regulation^17^ makes it unclear whether preexisting expression patterns constrain evolutionary outcomes.

Evolution of gene expression has been studied at genome-wide scales mainly using two distinct approaches: phylogenetic and pairwise analyses. Phylogenetic approaches model gene expression dynamics and infer ancestral expression patterns in the context of gene phylogenies. For example, Brownian motion models embody purely neutral expression evolution^18^, whereas Ornstein–Uhlenbeck (OU) models are designed to detect purifying selection and adaptive evolution along with neutral fluctuation^19–21^. Although each gene family has a distinct evolutionary history, a species phylogeny is often used for the sake of simplicity. Because such approximations cannot be applied to gene families with lineage-specific gene duplications and losses, its application has mostly been limited to single-copy genes. In contrast, pairwise analysis compares gene expression between paralogs in single species^22,23^ or between orthologs or paralogs in pairs of species^9,11,24–26^. Although pairwise approaches can evaluate the effect of gene duplications, ancestral expression cannot be inferred.

To infer the adaptive evolution of gene expression in diverse gene families, we apply OU models for complex gene family phylogenies containing gene duplications and losses, without assuming species phylogeny. We also develop a curation pipeline to amalgamate large amounts of transcriptome data from many studies for a better phylogenetic resolution.

The results of this study, using these methods and genome-scale datasets, show how gene duplication affects evolution of expression. As genes evolve, their patterns of expression occasionally shift from primarily one organ to another, forming modular connections. Our main conclusion is that these shifts are not random. When a shift occurs, the organ of primary expression for the ancestral gene strongly predicts the organ of primary expression for the descendant gene. We conclude that this supports a major role for what can be described as preadaptive pathways of gene expression evolution, by which we mean that adaptation of a gene for expression and presumably functional utility in one organ predisposes it to be more readily utilized for primary expression in another organ. A further result of this study is that expression shifts are larger and more frequent following gene duplication than in its absence. Each shift in gene expression may or may not be itself adaptive, but especially after gene duplication they are often accompanied by accelerated or decelerated rates of protein evolution. We conclude that this and the larger expression shifts observed following gene duplication support the idea that gene duplication tends to free genes up for regulatory or structural functional divergence, and sometimes both.

## Results

### Duplication-permissive genome-wide analysis of gene families

To allow evolutionary expression analysis on a broad set of genes, we used a phylogenetic approach that deals with the complex history of gene family trees with duplications and losses, and applied it to 21 tetrapod genomes (Supplementary Fig. 1). A major challenge in using gene trees was divergence time estimation, a prerequisite for applying phylogenetic comparative methods. We overcame this problem by incorporating phylogeny reconciliation in estimating divergence time of gene trees. Gene divergence nodes were constrained by the corresponding divergence times in a known species tree, and duplication nodes were constrained by ancestral and descendant speciation events (see “Methods” for details). Because we estimated individual gene phylogenies rather than using a single species phylogeny, we could analyze gene families that included many lineage-specific gene duplications and losses, making our study less biased toward conserved genes with slow gene turnovers^24^. Use of gene family trees also allowed us to include many organ-specific genes that are enriched in lineage-specific and young duplications^24^. There were only 1377 single-copy orthologs for which the species phylogeny was applicable, but we were able to include 15,475 genes per species on average (including 20,873 human genes, merged into 15,280 gene families). This approach eliminates problems with pairwise analyses that ignore phylogenetic tree structure, and allowed us to infer expression at ancestral nodes in the tree.

### Transcriptome amalgamation

To attain high resolution in our analyses, we amalgamated 1903 RNA-seq experiments from 182 research projects (i.e., 182 BioProject IDs in the NCBI SRA database) and generated a dataset covering six organs from 21 vertebrate species without missing data (Supplementary Data 1 and 2 and Supplementary Fig. 1). In comparison, other recent comparative transcriptomic analyses of vertebrates^20,24,26–32^ often used the same dataset containing 131 RNA-seq experiments from six organs and ten species^19^, with some additional data in different studies. RNA-seq reads were first mapped to corresponding reference genomes and then the expression level was quantified by two metrics: transcripts per million (TPM) and fragments per kilobase million normalized by trimmed mean of M-values^33^ (TMM-FPKM). To reduce the among-species variation, the TMM normalization was applied across all 1903 samples using the 1377 single-copy orthologs.

**Fig. 1:**
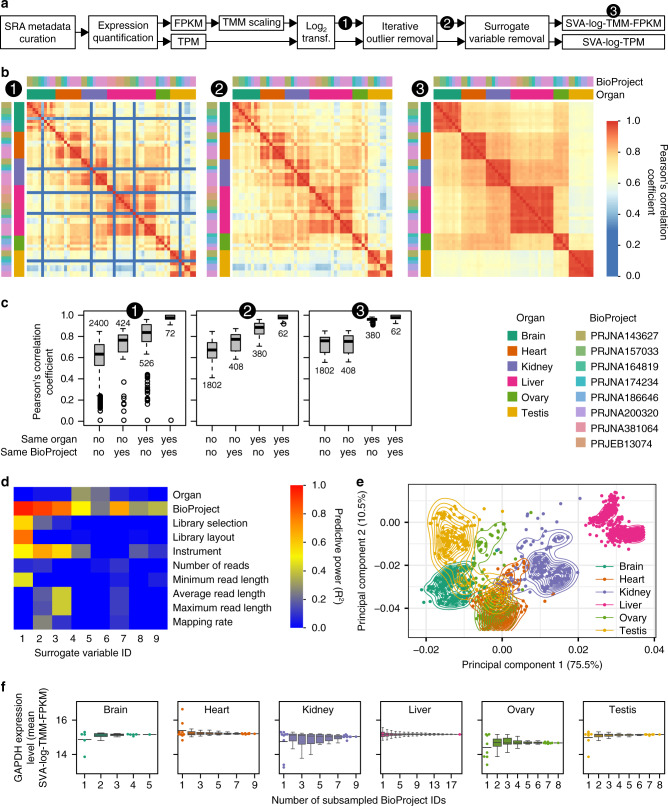
Transcriptome amalgamation to integrate heterogeneous RNA-seq samples. **a** A simplified flow chart of the transcriptome amalgamation. The full chart is available in Supplementary Fig. 1. **b**–**d** Transcriptome curation within species. Data from *Monodelphis domestica* with SVA-log-TMM-FPKM metrics are shown as an example. The heatmaps show Pearson’s correlation coefficients among RNA-seq samples (**b**). Each row and column corresponds to one RNA-seq sample. The expression levels of all genes were used to calculate the correlation coefficients. Note that anomalous samples contaminated in the curated metadata (low correlation samples in 1) are successfully removed, and that project-specific correlations visible in the uncorrected data (marked 2) are absent in the corrected data (marked 3). The boxplots show distinct distributions of the correlation coefficients depending on whether a pair of samples are the same organ or whether they are from the same research project (**c**). The numbers of comparisons are provided in the plot. The correlation coefficients are largely improved in within-organ comparisons when surrogate variables are removed, while within-project biases are attenuated. In this species, nine surrogate variables were detected against 52 RNA-seq data from eight projects (**d**). Analysis of those variables by linear regression highlights the BioProject feature as the strongest source of removed biases. For full description of predictors, see Supplementary Fig. 4. **e** A principal component analysis using expression levels of 1377 single-copy orthologs from 21 species. Points correspond to RNA-seq samples. Curves show the estimated kernel density. Explained variations in percentages are indicated in each axis. **f** Estimated organ-wise expression levels of a housekeeping gene. Since data from relatively many BioProjects are available, glyceraldehyde-3-phosphate dehydrogenase gene (GAPDH, Ensembl gene ID: ENSGALG00000014442) in *Gallus gallus* is shown as an example. Points correspond to the average expression level calculated by random subsampling. All data points and the median value (bar), rather than a boxplot, are shown if the number of subsampled BioProject combinations is <10. Boxplot elements are defined as follows: center line, median; box limits, upper and lower quartiles; whiskers, 1.5 × interquartile range; points, outliers.

To allow rapid integrated analysis of datasets, we employed automated multi-aspect quality controls, including metadata curation (Supplementary Data 3), sequence read filtering (Supplementary Fig. 2), and iterative removal of anomalous RNA-seq samples by monitoring correlations between and within data categories (Fig. 1a and Supplementary Fig. 3). The metadata curation enabled us to select appropriate samples from the NCBI SRA database. Data that were not compatible with those from other research projects (defined by BioProject ID) were removed in the correlation analysis by implementing a majority rule (Supplementary Data 3), resulting in a cleaned dataset. This filtering step was designed to fulfill the assumption that any samples from the same organ should correlate better than samples from different organs within species. When anomalous data were detected, all samples belonging to the same research projects (i.e., the same BioProject ID) were discarded.

Finally, we applied surrogate variable analysis (SVA)^34^ to detect and correct hidden biases likely originating from heterogeneous sampling conditions and sequencing procedures among experiments in both log_2_-scaled TPM and TMM-FPKM (SVA-log-TPM and SVA-log-TMM-FPKM, respectively; Supplementary Fig. 4a, b). This correction greatly improved the correlation of expression levels within organs from the same species, even when data were derived from different research projects (Fig. 1b, c and Supplementary Fig. 4c–f). Among surrogate variables, BioProject IDs tend to show a high predictive power, suggesting project-specific sources of bias (Fig. 1d and Supplementary Fig. 4g–h). Although the inclusion of many species from phylogenetically diverse lineages makes it difficult to extract organ-wise characteristics from the limited number of single-copy orthologs, a principal component analysis produced moderate organ-wise segregation in the multispecies comparison (Fig. 1e and Supplementary Fig. 4i–k), further indicating that the curated dataset is sufficiently reliable for use in cross-species expression pattern analyses. The previously-reported uniqueness of testis transcriptomes^19^ was partly resolved as the third principal component (Supplementary Fig. 4k).

To further evaluate the validity of amalgamated transcriptomes, we analyzed the expression of community-curated cell-type-specific marker genes associated with organs in PanglaoDB^35^, which organizes a number of single-cell RNA-seq experiments in human and mouse. We compared median values of log-transformed expression levels of >100 marker genes in each organ (Supplementary Fig. 5). After SVA correction, all RNA-seq samples in the both species showed the corresponding marker expression values higher than those from the other organs, suggesting our amalgamated transcriptomes preserve the organ-specific gene expression. In the cell-type-wise analysis, a few cases, such as juxtaglomerular cells in kidney and hepatic stellate cells in liver, could not resolve our organ-wise transcriptomes (Supplementary Dataset 10.17632/3vcstwdbrn.1). However, such low performance was seen in all samples rather than subsets associated with particular BioProject IDs, suggesting that the dissection decisions have negligible effects to cell-type compositions in the organs.

In addition to the better phylogenetic coverage, greater accuracy of estimated expression levels is another possible advantage of integrating many RNA-seq datasets. This idea is supported by subsampling analysis on a housekeeping gene glyceraldehyde-3-phosphate dehydrogenase, where, as more data are used, estimated expression levels in different organs tend to quickly converge to a similar range of values (Fig. 1f, ca. 15 SVA-log-TMM-FPKM; Supplementary Fig. 4l, ca. 11.5 SVA-log-TPM).

### Modeling expression evolution

**Fig. 2:**
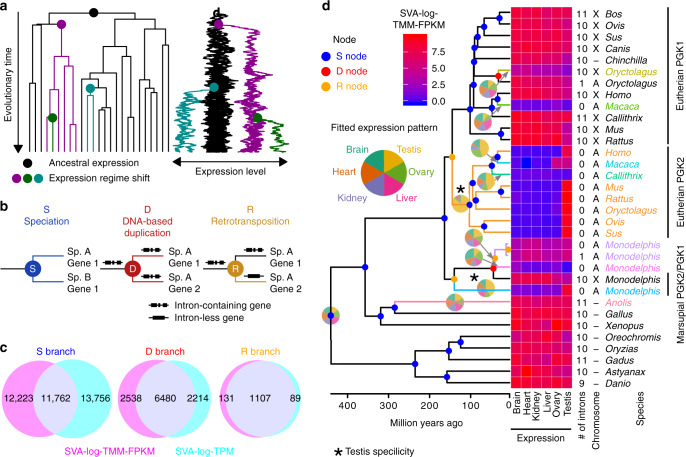
Expression evolution in a complex history of gene family evolution. **a** Modeling expression evolution with multi-optima Ornstein–Uhlenbeck process. A phylogenetic simulation is shown. Colors show branches belonging to different regimes. Regime shifts (change of color) appear as a substantial change in optimal trait values. **b** Nodes and branches of gene family phylogeny were categorized into S, D, or R based on the branching events, i.e., speciation, DNA-based duplication, or retrotransposition, respectively. **c** Venn diagrams of expression regime shifts. Circles represent the sets of branches where regime shifts were detected with SVA-log-TMM-FPKM or SVA-log-TPM. **d** The gene tree of phosphoglycerol kinases (orthogroup ID: OG0002332) is shown as an example. This orthogroup shows ortholog-specific expression patterns as well as regime shifts after speciation and lineage-specific gene duplication. Tips correspond to genes. The colors of branches and tip labels indicate expression regimes. Node colors match to the categorization in **b**. The heatmap shows expression levels and among-organ expression patterns are shown as a pie chart for each regime. To the right, the number of introns and located chromosomes (A, autosome; X, X chromosome; Y, Y chromosome) are indicated. For full information including complete tip labels and bootstrap branch supports, see Supplementary Dataset 10.17632/3vcstwdbrn.1.

We next used the amalgamated transcriptomes to evaluate how expression evolved along 15,280 maximum-likelihood gene family phylogenies (Supplementary Data 4), employing multi-optima OU models^36^ to allow for possible adaptive shifts of optimal expression levels and neutral fluctuations^19–21^. This modeling identified statistically supported expression regime shifts^36,37^ on each gene tree (Fig. 2a), which were then analyzed in the context of preceding duplication events. Speciation events (S node; Fig. 2b) with no duplication were considered the baseline mode of expression evolution because regulatory environments and expression patterns are more preserved among orthologous genes in comparison with paralogous genes produced by gene duplication^8–11^. Because OU shift detection has been applied for gene expression by assuming species tree phylogeny in single-copy genes, shifts in S branches are equivalent to those characterized previously^19,20^ but also include many more speciation events in duplication-prone gene families. Gene tree nodes associated with preceding duplication events were categorized as DNA-based duplication or retrotransposition (D or R nodes, respectively) depending on complete intron losses (Fig. 2b).

Organ-wise means of the two expression values, SVA-log-TPM and SVA-log-TMM-FPKM, were separately used to model expression evolution with OU processes. The two analyses resulted in similar numbers and characteristics of expression regime shifts (Supplementary Fig. 6), but the shift locations were sometimes inconsistent. S branches were a major source of apparently inconsistent regime shifts, whereas branches following duplication (D and R) showed largely consistent detection between the two metrics (Fig. 2c). Although the inclusion of inconsistently detected branches did not change the results, we retained only consistently detected regime shifts for all downstream analysis to draw a more robust conclusion. While SVA-log-TMM-FPKM values were reported in the main text unless otherwise mentioned, the comparisons with SVA-log-TPM-based analyses are available as Supplementary Information (see below for specific citations).

As an example of this analysis, an orthogroup of phosphoglycerol kinases (PGKs), containing all three categories of branching events followed by expression shifts (S, D, and R), is shown in Fig. 2d. This protein catalyzes the first ATP-generating step in the glycolytic pathway and is required for most cell types including sperms^38,39^. PGK1, the original copy on the X chromosome, is known to have duplicated independently in eutherians and marsupials to produce the autosomal retrocopy PGK2 that compensates the protein activity during X-inactivation^40–42^. Our automated analysis correctly recovered both retrotranspositions as well as the subsequent gains of testis-specific expression in eutherian and marsupial lineages. This illustrates that our automated genome-wide analysis can recover evolutionary trajectories that are compatible with focused single gene family analyses (see Supplementary Dataset 10.17632/3vcstwdbrn.1 for individual gene trees).

### Duplication-specific effects in expression evolution

**Fig. 3:**
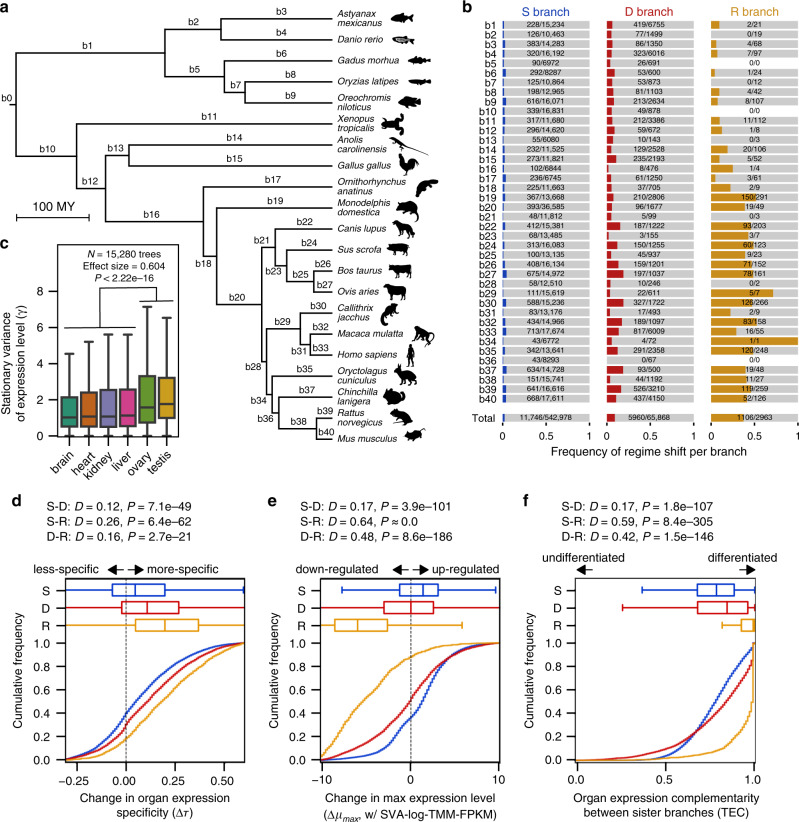
Characteristics of expression shifts in 15,280 gene trees. **a** The species tree showing analyzed genomes and their divergence time. A part of animal silhouettes were obtained from PhyloPic (http://phylopic.org). The silhouettes of *Astyanax mexicanus* and *Oreochromis niloticus* are licensed under CC BY-NC-SA 3.0 (https://creativecommons.org/licenses/by-nc-sa/3.0/) by Milton Tan (reproduced with permission), and those of *Anolis carolinensis* (by Sarah Werning), *Ornithorhynchus anatinus* (by Sarah Werning), and *Rattus norvegicus* (by Rebecca Groom; with modification) are licensed under CC BY 3.0 (https://creativecommons.org/licenses/by/3.0/). **b** Mapping of 18,812 expression shifts in the species tree. The number and proportion of expression regime shifts in S, D, and R branches are shown. Corresponding branches in the species tree are indicated in **a**. **c** Organ-specific stationary variances (*γ*) of expression level evolution in vertebrates. The distribution of *γ* between reproductive and nonreproductive organs were compared by a two-sided Brunner–Munzel test^95^. **d**–**f** Cumulative frequency of change in organ expression specificity (**d**), change in maximum expression level (**e**), and expression complementarity between sister lineages (**f**) among detected expression shifts. Number of analyzed regime shifts are shown in **b**. The *D* statistics and *P* values of pairwise branch category comparisons were calculated with two-sided Kolmogorov–Smirnov tests. Boxplot elements are defined as follows: center line, median; box limits, upper and lower quartiles; whiskers, 1.5 × interquartile range.

Across gene trees, per-branch frequencies of expression regime shifts were significantly different among S, D, and R branches (*P* ≈ 0; *χ*^2^ = 2.11 × 10^4^; *χ*^2^ test). Expression regime shifts were relatively rare in S branches, for a probability of 2.2% per branch, and an average rate of 2.5 × 10^−4^ shifts per MY (million years) (Fig. 3a, b). In agreement with the idea that gene duplication tends to free genes up for functional divergence and enhance long-term retention of duplicated copies^22,43^, the frequency of regime shifts in D branches was four times as much per branch (9.0% per branch, at a rate of 1.7 × 10^−3^ shifts per MY across all genes and all D branches). Thus, although far fewer branches are preceded by DNA-based duplication events (65,868 branches) than speciation events (542,978 branches), D branches account for over 33% of all regime shifts consistently detected by the two expression measures. We note that this result reinforces previous results on the ortholog conjecture, the idea that duplicated gene copies (paralogs) are more prone to expression shifts than orthologs^8–11^. R branches were far more likely to result in expression regime shifts (37.3% per branch, at a rate of 7.0 × 10^−3^ shifts per MY), but with only 2963 R branches, this resulted in only 1106 shifts (5.6% of the total). Translocated genes are more likely to shift expression than those that do not (Supplementary Fig. 7), in line with previous observations from the human genome^23^. While the expression shift frequency in S and D branches varies slightly across the phylogeny, R branches showed much stronger among-lineage heterogeneity, and had a particularly high frequency in the mammalian lineage (Fig. 3b). These retrotransposition-related expression changes may be related to the variation in the retrotransposition rate itself, which is known to vary across lineages^44,45^. Among-species heterogeneity in gene prediction quality may also be attributed to this pattern because early-diverging species tended to show higher percentages of missing single-copy orthologs than those in mammalian species (Supplementary Fig. 8; but see *Danio rerio*, *Oreochromis niloticus*, and *Oryctolagus cuniculus* as counterexamples). In the absence of regime shifts, expression levels varied most in ovary and testes, which had significantly higher average stationary variances than the other four organs on the basis of tree-wise stationary variance (Fig. 3c). This supports previously observed high variation of gene expression in testes^19^ and extends it to the reproductive organs of both sexes.

If expression regime shifts are due to functional divergence, it is highly relevant to characterize how expression properties changed from ancestral to derived regimes. To do this, we examined changes in organ expression specificity, maximum expression level, and organ expression complementarity. The specificity measure τ ranges from 0 for uniformly expressed genes to 1 for genes with highly specific expression^46^. The distributions of regime shifts in D and R branches are shifted toward greater organ specificity compared to shifts in S branches, with R branches creating the most specific expression (Fig. 3d). To characterize the on state transcriptional activity, we analyzed the maximum fitted expression levels among the six organs (*μ*_max_). D and R branches appear to be enriched for downregulation compared to S branches (Fig. 3e). Complementarity of organ expression patterns was measured to evaluate the differentiation between a pair of sister branches. We used a metric on the fitted organ-wise expression levels (*μ*) called TEC, which ranges from 0 for completely overlapping expression to 1 for mutually exclusive patterns^43^^.^ Nearly all branches with regime shifts (95%) had complementarity values >0.5, indicating that most regime shifts detected involve differentiation of expression patterns, rather than overall up- or downregulation. Regime shifts in D and R branches often had more complementary expression than those in S branches (Fig. 3f), further supporting the role of gene duplication in functional differentiation. The more drastic effect in R branches probably reflects the regular loss of regulatory elements in retrotranspositions, whereas DNA-based duplication can more often retain regulatory regions^47^. Jointly, these results indicate that, compared with the baseline from speciation-associated shifts, gene duplication tend to produce more organ-specific, more often downregulated, and more differentiated expression patterns. Although the downregulation may be explainable by a tendency to need less of the newly functional expression regime, it may also be explained by either recent nonfunctionalization^48,49^ or specialized expression in organs that were not part of this analysis.

### Context-dependent change in the rate of protein evolution

**Fig. 4:**
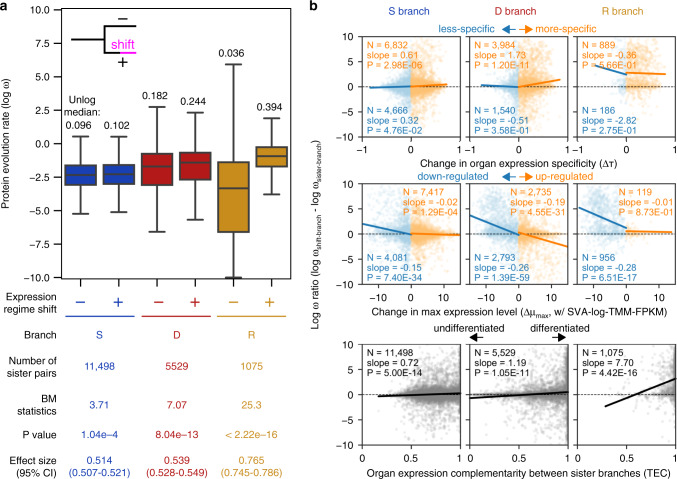
Context-dependent changes of protein evolution rate coupled with expression regime shifts. **a** Distribution of *ω* values. A plus (+) indicates branches with expression shifts, whereas minus (−) branches are sisters to the plus branches. Statistical differences between pairs of distributions were tested using a two-sided Brunner–Munzel test^95^. Non-log-transformed median values are shown above the boxplots. For visualization purposes, extreme values exceeding ±10 were clipped. Boxplot elements are defined as follows: center line, median; box limits, upper and lower quartiles; whiskers, 1.5 × interquartile range. **b** Relationships between protein evolution rate and change in expression properties. Points correspond to expression regime shifts. Dashed lines indicate no between-branch difference in *ω*. Solid lines show a linear regression. Its slope and number of regime shifts are provided in the plot. Regime shifts with negative and positive changes were separately analyzed for organ expression specificity (upper) and maximum expression level (middle). *P* values indicate whether the slopes were significantly different from zero (two-sided *t* tests).

Change in gene expression can be accompanied by accelerated or decelerated protein evolution, which may be detected by change in the ratio of nonsynonymous/synonymous substitutions (*dN/dS* or ω) along branches. In D and R branches, median *ω* values are more than double the baseline seen in S branches (Fig. 4a; Supplementary Fig. 9a), again supporting the ortholog conjecture and the idea that gene duplication tends to free genes up for functional divergence^22,43^. Within each of S, D, and R branch categories, branches with expression regime shifts accompany an increased *ω* compared to sister branches (Fig. 4a). The increased rate was quite small in S branches (median *ω*, 0.096 in branches with shifts versus 0.102 in sister branches), much bigger in D branches (0.182 versus 0.244), and huge in R branches (0.036 versus 0.394). If the changes in expression and the rate of protein evolution are due to functional changes, this may indicate that functional divergence is sometimes effected by joint changes in expression and accelerated protein evolution.

Although 52.3% of all branches with expression regime shifts had higher *ω* relative to sister branches (*ω* ratio), 27.5% of sister branch pairs are relatively undifferentiated, with differences in *ω* within ±5%, and 42.5% had lower *ω* in the branches with expression shifts. This finding led us to hypothesize that the direction of the rate changes in protein evolution is linked to how, rather than whether, expression is changed. Strikingly, we found a context-dependent association between protein and expression evolution (Fig. 4b and Supplementary Fig. 9b). Increased *ω* ratio was linked strongly to increased, rather than decreased, organ expression specificity in S and D branches, potentially reflecting adaptive evolution coupled with specialized expression. However, it was in turn highly associated with decreased specificity in R branches, which may be explained by frequent gene decay in unsuccessful retro-copies. The change in maximum expression level was overall negatively correlated with *ω* ratio, but this link was stronger in downregulation compared with upregulation, except for D branches where the *ω* ratio was smaller when Δ*μ*_max_ was larger (Fig. 4b). It has been reported that high expression slows protein evolution^1^, and our results suggest that DNA-based duplication creates such constraints when accompanied by upregulation. The organ expression complementarity between sister lineages was positively correlated with *ω* ratio (Fig. 4b), and its association was strongest in R branches, suggesting that protein evolution accelerates as gene expression patterns differentiate from their ancestral state. Collectively, these results suggest that protein evolution rate is linked to changes in expression properties through a complicated association, which masks their relationships in a global, unstratified analysis, and potentially explain a previous report of no strong relation^26^.

### Organ-specific propensity in gene expression evolution

The preadaptation hypothesis predicts that the ancestral organ expression prior to the shifts will affect which organs are likely to become the target of newly specific expression. To assess this, we tested whether expression shifts are random with respect to change in expression from one organ to another, by characterizing the organ in which genes are most highly expressed (primary-expressed organ, PEO).

**Fig. 5:**
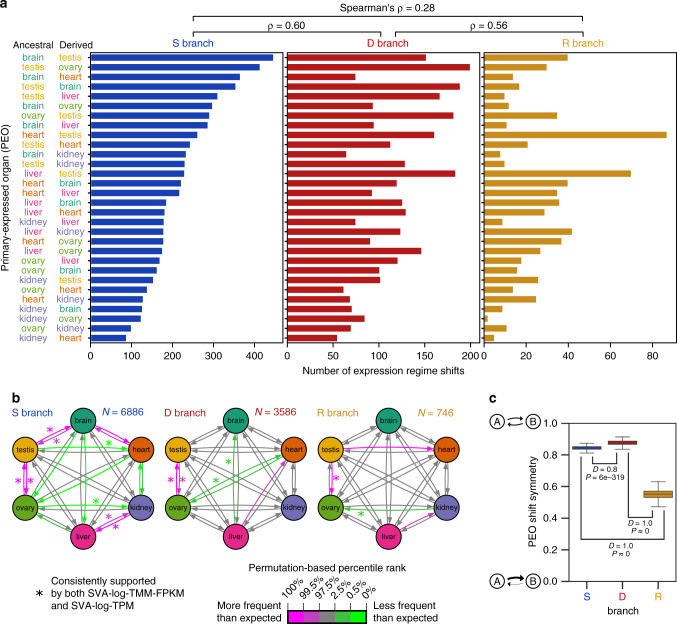
Evolutionary dynamics of gene expression. **a** Shift distributions of primary-expressed organs (PEOs). *Y*-axis was sorted by abundance in S branches. Spearman’s correlation coefficients among S, D, and R branches are shown above the plots. **b** Preadaptation networks in organ expression. Arrows represent transitions from ancestral PEOs to derived PEOs, and its color shows statistical significance based on 10,000 permutations. The results were obtained with SVA-log-TMM-FPKM, and an asterisk (*) indicates the statistical significance supported also by SVA-log-TMM-based analysis (Supplementary Fig. 11). **c** The global polarity of PEO shifts. The global polarity is defined by the scaled sum of differences between two opposite PEO shifts. Boxplots show the distribution estimated by 1000 bootstrap resampling. The *D* statistics and *P* values of pairwise branch category comparisons were calculated with two-sided Kolmogorov–Smirnov tests. Boxplot elements are defined as follows: center line, median; box limits, upper and lower quartiles; whiskers, 1.5 × interquartile range.

Across vertebrates, switching from one PEO to another was detected in 6886, 3586, and 746 regime shifts in S, D, and R branches, respectively. The gain/loss ratios are heterogeneous among organs (Fig. 5a), suggesting that vertebrate organs serve as both sources and sinks in expression evolution, but that their relative contributions are organ-specific. Although S, D, and R branches shared a global trend of relatively abundant testis-related PEO shifts, their distributions are largely different (*P* = 1.52 × 10^−77^; *χ*^2^ = 531; *χ*^2^ test). D branches were moderately similar to both S and R branches (Spearman’s *ρ* ~ 0.6), but S and R branches were dissimilar (*ρ* = 0.28). This pattern, including the abundant shifts related to testis, was robust against the correction by the organ-wise numbers of expressed genes (Supplementary Fig. 10). This result suggests a role for gene duplication, including by retrotranspositions, in remodeling the among-organ flow of expressed genes.

Controlling the total number of shifts from and to each PEO, some PEO shifts are significantly different from the random expectation (Fig. 5b and Supplementary Data 5). There are clear patterns of evolutionary transitions that are statistically supported by independent OU modeling of the two expression metrics. In S branches, the pairs of brain–testis and testis–ovary showed strong connections, indicating a solid exchange module. Kidney and liver also donate genes to one another, forming a separate module from brain–testis–ovary. D and R branches showed a pronounced acceleration of PEO shifts between testis and ovary. PEO shifts in S and D branches were moderately symmetric in the flow between pairs of organs (Fig. 5c), meaning two organs tend to donate comparable numbers of expressed genes each other. In contrast, R branches were more asymmetric. The lower symmetry may have perturbed the evolutionary dynamics of gene expression.

To check the robustness of our analysis, we analyzed high-confidence subsets of expression regime shifts. The most drastic expression changes were characterized by introducing a cutoff of organ expression specificity (*τ* > 0.5) to define organ-specific genes. Although some previously significant trends were not recovered due to small sample sizes, the result was largely consistent with the broader analysis (Supplementary Fig. 11). Because tree inference errors can bias the downstream analysis including OU modeling, we also analyzed expression regime shifts found in clades which have a high support in tree inference (>99% bootstrap support). Again, the results were largely consistent (Supplementary Fig. 11). Especially, the brain–testis–ovary and kidney–liver modules in S branches and the testis–ovary connection in D and R branches were always reproduced in the analyses with the above thresholds in combinations with the two expression metrics (SVA-log-TMM-FPKM and SVA-log-TPM; Supplementary Fig. 11). The analysis of shifts in high-support branches also reproduced the other main results in this paper (Supplementary Dataset 10.17632/3vcstwdbrn.1), demonstrating the robustness of our conclusion.

The effect of gene trees was further examined by replicating the analysis with alternative tree topologies inferred by species tree reconciliation, which takes into account duplication–loss rates^50^. This reconciliation step is expected to correct erroneous tree topology, while possibly introducing another bias derived from over-correction of biological signals such as incomplete lineage sorting. With the reconciled trees, the OU modeling with SVA-log-TMM-FPKM values resulted in equivalent numbers of expression shifts in S and D branches compared with those with non-reconciled trees (97% [23,231/23,985] and 104% [9407/9018], respectively) (Supplementary Fig. 12a). In contrast, the phylogeny reconciliation substantially reduced the number of shifts in R branches (39% [481/1238]). This could be explained by the correction of erroneous tree topology caused by the fast-evolving retro-copies (Fig. 4a), although the differences in shift numbers did not correlate with the topological differences measured by the Robinson–Foulds distance^51^ (Supplementary Fig. 12b). Nevertheless, resulting PEO shift distributions were largely similar (Supplementary Fig. 12c), with the reproduced accelerations in the brain–testis–ovary and kidney–liver modules (Supplementary Fig. 12d) and the asymmetric PEO shifts in R branches (Supplementary Fig. 12e), suggesting the robustness of detected modules against gene tree topology.

To obtain insight into the biological relevance of the among-organ modules, we characterized human genes involved in PEO shifts from all branch categories using the Kyoto Encyclopedia of Genes and Genomes (KEGG) pathway enrichment analysis. The genes descended from the testis–ovary PEO shifts enriched only one KEGG pathway term “cell cycle” (Supplementary Data 6; adjusted *P* value < 0.05, Fisher’s exact test with the Benjamini–Hochberg correction), likely reflecting their function in meiosis. Although the adjusted *P* value was not statistically significant, it is noteworthy that the top-ranked term for the brain–testis connection was “endocrine and other factor-regulated calcium reabsorption” (unadjusted *P* value = 1.66 × 10^−3^; adjusted *P* value = 0.26) annotated to four genes including GNAQ, which has been implicated to tumor formation in neuronal tissues^52,53^. In the kidney–liver module, 15 terms were significantly enriched, many of which appear to be related to the functions and diseases in those organs, for example, “bile secretion,” “phagosome,” “lysosome,” “ABC transporters,” “sphingolipid metabolism,” and “Type-I diabetes mellitus” (Supplementary Data 6). These results suggest that the among-organ modules in the PEO shifts played a role in supplying functionally important genes.

Because ancestral expression was shown to orient new expression by the analysis of PEO shifts, we concluded that there was prevalent organ-specific propensity, which supports the presence of preadaptation in gene expression.

## Discussion

Our results suggest that the landscape of expression evolution is strongly shaped by mechanisms of gene birth. Expression shifts are more pronounced following gene duplication in agreement with the results of pairwise gene expression analyses^22,24,43^, and shifts in patterns of PEOs strongly depend on the expression state in the ancestral organism. Thus, by analyzing such influences on a genome-wide scale for a moderately large number of species, the question whether long-term expression in one organ predisposes genes to be subsequently utilized in other organs has been answered in the affirmative. There are preadaptive propensities in the evolution of vertebrate gene expression, and the propensity varies with the presence and type of gene duplication. Furthermore, the approach developed in this study, using complex gene family phylogenies including gene duplications and losses that do not assume perfect match to the species phylogeny, and incorporating a curation pipeline to amalgamate large amounts of transcriptome data from many studies, was essential to obtain the necessary species density and phylogenetic resolution to answer this question. The extensibility of this method will allow for more species and more organs to be incorporated as further studies come into the literature from diverse laboratories.

The mechanisms responsible for the preadaptive propensities that influence expression shifts among organs are, however, unknown. A key question in understanding these shifts may be the role of adaptation in the shift, and in subsequent evolution. We have been careful so far to simply describe the shifts, but adaptive possibilities include subfunctionalization, escape from adaptive conflict (EAC), and neofunctionalization^54–56^. The increased number of shifts following duplication suggests that drift alone is not the explanation, but subfunctionalization easily could be. Subfunctionalization is the idea that, if a gene has multiple functions prior to duplication, they may be segregated among the duplicates following gene duplication. Thus, the expression shifts may be simply a shift in focus of a duplicated copy on a subset of the necessary expression profile needed at the organismal level. In this scenario, any accompanying acceleration of amino acid substitution would be caused by a loss of constraint and reduced purifying selection in one expression environment or the other.

EAC involves more adaptation by adding the simple idea that prior to duplication, the multiple functions and expression regimes were at least partially in conflict. Such conflicts could clearly occur at the amino acid level, but could also occur at the expression level. For example, if expression levels were focused on a most-important tissue or most-sensitive tissue prior to duplication, but after duplication could be more tailored to what is better for the new expression regime. Finally, neofunctionalization would occur at the sequence or expression level, if the loss of selection on a duplicate allowed mutations that were previously harmful to the old function, but now are not, and are able to carry out some novel functional aspect that was previously prohibited. Neofunctionalization is perhaps the most interesting and extraordinary possible cause for the expression regime shifts we see, but it requires strong evidence and it is not a necessary explanation for what we observe.

In this context, the patterns of expression regime shifts we observed may be explained at different levels of biological organization, from the tissues and cells that make up organs, to subcellular compartments, chromatin structure, promoter usage, and protein biochemistry. Part of the propensity shifts we observed can be explained by the out-of-the-testis hypothesis, which posits that accelerated gains of testis expression are based on the permissive chromatin state, abundant transcriptional machinery, relatively simple promoters required for the expression in spermatogenic cells, and following gains of new expression patterns^4,57^. This theory fits to the accelerated testis-related PEO shifts, and could fit with any of the adaptive scenarios discussed above, but the other detected patterns (e.g., kidney–liver module) require other explanations.

One potential mechanism of preferences in expression regime shifts is a cell-type or subcellular component mechanism. In such a mechanism, if two organs tend to share cell types or usage of subcellular components, they may be prone to expropriate genes between the two organs. It is known that gene expression levels in the kidney and liver tend to change jointly, possibly reflecting their similar physiology including detoxifications and waste excretion^19^. Such functional similarity may also explain the presence of the kidney–liver module of gene exchange.

Another possibility is a regulatory mechanism whereby frequent gene-exchanging organs use similar sets of regulatory elements. Altered expression between such organs could occur with relatively few mutations in regulatory sequences. Cis-regulation is indeed a major source of expression evolution, as it explains a certain fraction of expression variability, for example, in budding yeast (30% in duplicates and 19% in singletons) and undergoes a more rapid divergence than trans-regulation^58^.

Finally, another possible mechanism for expression regime shifts following gene duplication is at the protein level. If frequently interacting organs have similar environmental requirements for expressed proteins, a few amino acid substitutions may tend to be required to adjust biochemical properties. Protein reusability may be determined by cellular environments such as pH and temperature or by functional categories of proteins. Our analysis indicates that the regime shifts that drastically differentiate the expression tend to be coupled with accelerated protein evolution (Fig. 4b), and this result can be viewed as a support for a protein-level mechanism. In such a mechanism, synergistic resolution of EAC may be a driving force for changes in both amino acid composition and expression regimes. We note that these mechanistic hypotheses are not mutually exclusive, and varying combinations of factors may contribute to generate preadaptive patterns of gene expression.

In this study, we established a method to standardize RNA-seq data from disparate research projects and developed a pathway for data amalgamation. Thanks to multiple rounds of innovations in sequencing technology, transcriptome data are being produced at an unprecedented rate in a greater variety of organisms and samples, such as those for multispecies multi-organ developmental series^59^. The transcriptome amalgamation will expand the use of such resources to study gene expression evolution.

By reconstructing gene expression in gene family phylogenies, our analysis revealed nonrandomness and directionality of expression evolution. This suggests prevalent preadaptation in gene expression, and that adaptation to expression in certain organs is more conducive to future expression in other organs. This provides further details on how gene duplication has helped to reshape the dynamics of expression evolution that contributed to the vertebrate diversification.

## Methods

### Species selection

A total of 105 species included in the Ensembl release 91^60^ were searched for data availability in the NCBI SRA database^61^ (final search on May 1, 2018) and 22 species were found to have RNA-seq data for six organs: brain, heart, kidney, liver, ovary, and testis. *Lepisosteus oculatus* was excluded due to an insufficient quality of available expression data, and therefore remaining 21 species were selected for further analysis.

### Species tree

The dated species tree for the 21 species was retrieved from TimeTree^62^ (downloaded on March 15, 2018; Supplementary Dataset 10.17632/3vcstwdbrn.1). Some species were unavailable in the database and therefore they were temporarily replaced by closely related species to obtain the dated species tree.

### Gene sets

Coding sequences (CDS) were retrieved from the Ensembl database. The longest transcript was retained when multiple transcripts were annotated to the gene. The quality of gene sets was evaluated using BUSCO 4.0.5^63^ with the single-copy ortholog set vertebrata_odb10 (Supplementary Fig. 8).

### Transcriptome metadata curation

We developed an automated python program for SRA metadata curation (Supplementary Data 3 and Supplementary Dataset 10.17632/3vcstwdbrn.1). RNA-seq data were selected from the NCBI SRA database by keyword searches limited to the 21 species, the six organs, and Illumina sequencing platforms. Orthographical variants of annotations were standardized with keyword libraries created by manually checking the original annotations. Prenatal or unhealthy samples and small-scale sequencing samples (<5 million reads) were excluded. Data for non-messenger RNA sequencings were also removed. In treatment and control RNA-seq pairs, only control experiments were included.

### Transcriptome quantification

Fastq files were extracted from downloaded SRA files using parallel-fastq-dump 0.6.2 (https://github.com/rvalieris/parallel-fastq-dump) with the minimum read length of 25 bp and the quality filter (-E option)^61^. The fastq sequences were then subjected to a quality filtering by fastp 0.12.3^64^. The filtered reads were mapped to genomic features annotated as non-messenger RNAs in the Ensembl GTF files using bowtie2 2.3.4^65^ and resultant unmapped reads were used for expression level quantification using kallisto 0.43.1 with the sequence-based bias correction^66^. Samples were removed if 20% or smaller percentages of reads were mappable (Supplementary Fig. 2). Estimated mapped read counts and transcript lengths were used to calculate TPM and FPKM values. For the latter, the “TMM” normalization method was applied^33^. Sample-wise TMM scaling factors were obtained across all RNA-seq samples using the FPKM values of 1377 single-copy orthologs. Because the TMM normalization destroys the estimated relative abundance of TPM, in which, by definition, the total counts must be 10^6^, this scaling method was applied only to FPKM values, but not to TPM values. TPM and TMM-FPKM values were subsequently transformed to *log* (*N* + 1) values (log-TPM and log-TMM-FPKM, respectively). Paralogous genes that haven’t diverged in their nucleotide sequences could not be distinguished well in the quantification step. Although our scope is to characterize gene expression in the timescale of vertebrate evolution, this difficulty likely leads to an underestimation of expression regime shifts in young duplicates.

### Iterative anomalous sample removal followed by SVA

Anomalous RNA-seq samples were iteratively removed by a correlation analysis. Pearson’s correlation coefficients were calculated for every RNA-seq data against mean expression level in each organ generated by averaging all other data excluding those from the same BioProject (Supplementary Fig. 3). We assume that the sample’s correlation coefficient against the same organ is higher than any of the values against the other organs, and we removed all samples from the same BioProject when violations were found. These steps were repeated until no violations were left and SVA-corrected expression levels were finally reported (SVA-log-TMM-FPKM and SVA-log-TPM; with sva function in an R package sva)^34^. We assume that the sample’s correlation coefficient against the same organ is higher than any of the values against the other organs, and we removed all samples from the same BioProject when violations were found. These steps were repeated until no violations were left and SVA-corrected expression levels were finally reported (SVA-log-TMM-FPKM and SVA-log-TPM). The curation steps were skipped if multiple samples were unavailable in the species and hence SVA analysis was inapplicable. The final dataset was comprised of 1903 RNA-seq experiments from 182 BioProjects that cover six organs from 21 vertebrate species without missing data (Supplementary Data 1 and 2).

### Orthogroup classification

Orthogroups, which contain all genes descended from one gene in the common ancestor, were inferred from CDS of the 21 species using OrthoFinder 2.1.2^67^ guided by the species tree. In total, 17,896 orthogroups were generated. The largest orthogroup, which comprised 7893 olfactory receptor genes, was removed from the analysis because of computational burden. After sequence alignment processing (see “Multiple sequence alignment”), we removed small orthogroups, which retained less than four genes and orthogroups that showed no parsimony informative sites, because phylogenetic relationships cannot be inferred. As the result of filtering, 15,280 orthogroups were left for OU modeling, with the largest one containing 3796 zinc-finger proteins (Supplementary Data 4).

### Multiple sequence alignment

Multiple fasta files containing CDS were generated for each orthogroup. Stops and ambiguous codons were masked as gaps (for implementation, see https://github.com/kfuku52/cdskit). In-frame multiple codon sequence alignments were generated using MAFFT 7.394 with the auto option^68^ and tranalign in EMBOSS 6.5.7.0^69^. Anomalous genes were excluded by MaxAlign^70^, which decreased the largest orthogroup size from 3796 to 2382 genes. Spurious codons were removed in-frame using pgtrimal in Phylogears2-2.0.2016.09.06 (https://www.fifthdimension.jp/products/phylogears/) with the gappyout option^71^.

### Gene tree reconstruction

Maximum-likelihood trees were reconstructed using IQ-TREE 1.6.5^72^ with the best-fit nucleotide substitution models selected by ModelFinder with the Bayesian Information Criterion^73^. Larger orthogroups and longer genes tended to fit more complex substitution matrices and larger numbers of categories of rate heterogeneity (Supplementary Fig. 13a, b and Supplementary Data 4). Ultrafast bootstrapping with 1000 replicates was performed to evaluate the credibility of tree topology^74^ with a further optimization of each bootstrapping tree (-bnni option)^75^. To evaluate the effect of alternative gene tree topologies, we performed phylogeny reconciliation using GeneRax 1.0.0^50^ with the maximum-likelihood gene trees and the species tree as input. Because rooted trees were generated in this step, the tree rooting (described below) was skipped for the reconciled trees.

### Reconciliation-assisted gene tree rooting

Candidate rooting positions were inferred with different methods. Using the dated species tree, all rooting branches with the minimum duplication–loss score were identified using the rooting mode of NOTUNG 2.9 with the default parameters (duplication score = 1.5, loss score = 1.0)^76^. The midpoint of the longest path^77^ and the position with the minimal ancestor deviations^78^ were also considered as candidates. The final rooting position was reported based on overlaps among those rooting positions (Supplementary Fig. 13c, e and Supplementary Data 4).

### Reconciliation-assisted divergence time estimation

To prepare dated gene trees, we first matched species tree nodes with corresponding gene tree nodes using the reconciliation mode of NOTUNG 2.9^76^ and created time constraints of speciation nodes (Supplementary Fig. 13f). Duplication nodes were constrained with the upper and lower age limits derived from corresponding speciation nodes. If the root node is a duplication node and is not covered by the range of the species tree, the upper age limit was set to 1105 million years ago, which corresponds to the split of animals and fungi^62^. Divergence time was then estimated by a penalized likelihood method^79^ implemented in an R package ape (chronos function with discrete model)^80^ with time constraints on speciation, duplication, and root nodes. When reasonable initial parameters were not found after 1000 trials, the above constraints were partly relaxed (Supplementary Fig. 13d and Supplementary Data 4). The implementation is provided on GitHub (https://github.com/kfuku52/RADTE).

### Modeling and shift detection of expression evolution

Using the dated gene trees and organ-wise mean values of SVA-log-TMM-FPKM and SVA-log-TPM, regime shifts in gene expression were detected as shifts of optimal trait values in OU models determined by a Lasso-based model selection with AICc in an R package *l*1ou^37^. Because there is no available software to handle within-species variation in phylogenetic OU shift detection without predefined hypotheses on the number and place of regime shifts, we used mean expression level as the input. It is shown by simulation that the species mean and species variance models show comparable power in the regime shift detection^21^, suggesting that our species mean model is expected to perform as good as the species variance model. In the model, *α* and *σ*^2^ parameters were assumed unchanged in the tree^37^, and therefore only the global, rather than branch- or clade-wise, stationary variance (*γ*) were obtained. Expression levels in the six organs were treated as multivariate traits where *α* and *σ*^2^ were estimated for each organ but regime shifts were assumed to occur jointly in the same set of branches^37^. The phylogenetic mean (expression level at the root node) was estimated with the OUfixedRoot model. To handle gene trees recalcitrant to this analysis (especially those with a large number of genes), we skimmed gene trees by collapsing clades with small changes in expression level (Supplementary Fig. 14). Specifically, we first calculated all-vs.-all Pearson’s correlation coefficients of gene expression level among all genes that belong the clade. The clade was collapsed into a single tip if the expression patterns were almost identical (minimum correlation coefficient between genes > 0.99). Phylogenetic means of the collapsed clade were calculated by assuming the Brownian motion and were used as expression level at the new tip. The upper limit of regime shifts was set as max[min(*N*/2, 100), $\root {2} \of {N}$], except for the largest tree with 2382 genes where the upper limit was decreased to 10 to cope with an unrealistically large number of branch combinations to consider. The number of detected regime shifts was always smaller than the upper limit in the SVA-log-TMM-FPKM analysis, whereas three out of 15,820 trees reached the upper limit in the SVA-log-TPM analysis (Supplementary Fig. 6a).

### Analysis of expression pattern

We characterized expression patterns of extant and ancestral genes by calculating different metrics from fitted values (*μ*) in the OU models. Organ specificity was measured by *τ*^46^, which outperformed other methods in a benchmark for tissue specificity^81^. Expression complementarity between sister lineages was measured by the metrics called TEC^43^. Because *μ* was estimated from log-transformed expression levels, these expression metrics were calculated with unlog-transformed *μ* values. PEOs were defined as the organ in which the highest expression levels were observed among the six organs we analyzed.

### Estimation of protein evolution rate

Parameters for codon substitution matrix, shape parameter of discrete gamma distribution for rate heterogeneity (*α*), equilibrium transition/transversion rate ratio (*κ*), equilibrium nonsynonymous/synonymous substitution rate ratio (*ω*) were estimated using IQ-TREE 1.6.5^72^ by fitting GY+F3X4+G4 models to each gene tree. Equilibrium base composition (*θ*) was estimated from empirical codon state frequencies, which are calculated from the alignment by counting. To obtain *θ* at the root node, we calculated *θ* at subroot nodes by taking advantage of IQ-TREE’s empirical Bayesian method for ancestral sequence reconstruction. Considering subroot branch length, a weighted average of the subroot *θ* values were calculated as the *θ* value at the root node. Using all those parameters, branch-wise nonsynonymous/synonymous substitution rate ratios were estimated by stochastic substitution mapping (*mapdNdS*)^82^ using bio++ library^83^. To examine the robustness of the *mapdNdS*-based *ω* estimation, we compared the results with those obtained by maximum-likelihood *ω* estimation by fitting MG94W9 models in HyPhy 2.3.11^84^. The two methods yielded consistent results on the effect of branching events and expression shifts (Fig. 4a and Supplementary Fig. 9a), suggesting methodological robustness. We reported *mapdNdS*-based results in the main text.

### Analysis of gene structure and location

The number of introns and chromosomal location were obtained for each gene from the Ensembl gene models (GFF3 files). The intron numbers were subsequently converted to binary values that represent intronless and intron-containing states. Chromosomal locations were categorized into autosome, X chromosome, and Y chromosome. Genes from non-therian species were treated as missing data because mechanisms of their sex determination are not homologous to the mammalian XY system^85^. Genes from *Chinchilla lanigera* were also treated as missing data because their sequenced genomes are not anchored to chromosomes in the Ensembl release 91. The posterior probabilities of ancestral character states were inferred by the stochastic character mapping of discrete traits^86^ implemented in an R package phytools^87^. Because functional retrotranspositions^44,88^ and interchromosomal duplications^89^ are rare events relative to the timescale of the vertebrate evolution on the per-gene basis, we set the transition rate parameters to a sufficiently small value (1 × 10^−3^ per gene per million years). Since intron gain occurs few orders of magnitude less frequently than its loss caused by retrotransposition and others^90^, the rate of intron gain is set to be lower (1 × 10^−4^ per gene per million years).

### Analysis of branching events

Speciation and duplication nodes (S and D/R nodes, respectively) were classified by a species-overlap method^91^ and were mapped to the species tree on the basis of species coverages of the gene tree clades. A transition from intron-containing to intronless states with a posterior probability >0.5 was classified as a retrotransposition event (R node). The branches that correspond to the original copy of a retrotransposition event were not included in R branches. Although our classification cannot detect retrotranspositions from originally intronless genes, we expect such situations would be rare because most vertebrate genes contain at least one intron (e.g., 20,160/21,242 human genes). Interchromosomal translocation was detected by considering chromosomal locations with the highest posterior probability as the ancestral states. Because of the difficulty in determining rooting positions of deep phylogenies, gene tree nodes older than the root node of the species tree were removed from the analysis.

### KEGG pathway enrichment analysis

Human genes that descend from the shift branches were pooled for each specific PEO shift. The gene lists were examined for enrichment against an Enrichr library KEGG_2019_Human^92^ using a python package GSEApy (https://github.com/zqfang/GSEApy). Statistically significant (adjusted *P* value < 0.05) KEGG pathway terms were reported in Supplementary Data 6.

### Data visualization

Phylogenetic trees were visualized using a python package ETE 3^93^ and an R package ggtree^94^. A part of animal silhouettes in Fig. 3a and Supplementary Fig. 4 were obtained from PhyloPic (http://phylopic.org). The silhouettes of *Astyanax mexicanus* and *Oreochromis niloticus* are licensed under CC BY-NC-SA 3.0 (https://creativecommons.org/licenses/by-nc-sa/3.0/) by Milton Tan (reproduced with permission), and those of *Anolis carolinensis* (by Sarah Werning), *Ornithorhynchus anatinus* (by Sarah Werning), and *Rattus norvegicus* (by Rebecca Groom; with modification) are licensed under CC BY 3.0 (https://creativecommons.org/licenses/by/3.0/). Boxplot elements of all figures are defined as follows: center line, median; box limits, upper and lower quartiles; whiskers, 1.5 × interquartile range; points, outliers. Boxplot outliers are suppressed in Figs. 3 and 4 and Supplementary Figs. 9, 11, and 12.

### Reporting summary

Further information on research design is available in the Nature Research Reporting Summary linked to this article.

## Supplementary information

Supplementary Information

Peer Review

Reporting Summary

Description of Additional Supplementary Files

Supplementary Data 1-6

## Data Availability

Gene expression values including SVA-log-TMM-FPKM and SVA-log-TPM and other data used in this study are available as Supplementary Data 1–6 and Supplementary Dataset (10.17632/3vcstwdbrn.1). NCBI SRA accessions for the RNA-seq datasets analyzed in this study are available in Supplementary Data 1.

## Code availability

All codes used in this study are available from the following link: 10.17632/3vcstwdbrn.1.
