# Author Correction: Amalgamated cross-species transcriptomes reveal organ-specific propensity in gene expression evolution

**DOI:** 10.1038/s41467-021-25658-5

**Published:** 2021-09-02

**Authors:** Kenji Fukushima, David D. Pollock

**Affiliations:** 1 Department of Biochemistry and Molecular Genetics, University of Colorado School of Medicine, Aurora, CO 80045 USA; 2 Present Address: Institute for Molecular Plant Physiology and Biophysics, University of Würzburg, Würzburg, Germany

**Keywords:** Gene regulatory networks, Molecular evolution, Evolutionary biology, Gene expression

Correction to: *Nature Communications* 10.1038/s41467-020-18090-8, published online 8 September 2020.

The original version of this Article contained an error in “Methods”, which incorrectly read

‘**Iterative anomalous sample removal coupled with SVA**

Anomalous RNA-seq samples were iteratively removed by a correlation analysis coupled with an expression bias correction. In each iteration, unwanted biases in expression level were removed by SVA (sva function in an R package sva)^34^. SVA analysis was applied at the beginning of each iteration so that it is not influenced by anomalous samples removed in previous iterations. Subsequently, Pearson’s correlation coefficients were calculated for every RNA-seq data against mean expression level in each organ generated by averaging all other data excluding those from the same BioProject (Supplementary Fig. 3).’

The correct version replaces these sentences with

‘**Iterative anomalous sample removal followed by SVA**

Anomalous RNA-seq samples were iteratively removed by a correlation analysis. Pearson’s correlation coefficients were calculated for every RNA-seq data against mean expression level in each organ generated by averaging all other data excluding those from the same BioProject (Supplementary Fig. 3). We assume that the sample’s correlation coefficient against the same organ is higher than any of the values against the other organs, and we removed all samples from the same BioProject when violations were found. These steps were repeated until no violations were left and SVA-corrected expression levels were finally reported (SVA-log-TMM-FPKM and SVA-log-TPM; with sva function in an R package sva)^34^.’

This has been corrected in both the PDF and HTML versions of the Article.

