# Supplementary material for: Amalgamated cross-species transcriptomes reveal organ-specific propensity in gene expression evolution
Source: Nat Commun. 2020 Sep 8;11:4459. doi: 10.1038/s41467-020-18090-8 (PMC7479108; doi:10.1038/s41467-020-18090-8)
Supplement: Supplementary file 1 — Supplementary Information [file 41467_2020_18090_MOESM1_ESM.pdf]

Supplementary Materials for

Amalgamated cross-species transcriptomes reveal organ-specific propensity in gene expression evolution

Kenji Fukushima and David D. Pollock

List of Supplementary Materials:

Supplementary Figs. 1–14

Supplementary References

Supplementary Data 1–6 (separate file)

Supplementary Dataset (separate files available at http://dx.doi.org/10.17632/3vcstwdbrn.1)

Table of Contents:

Supplementary Fig. 1. A flow-chart of transcriptome amalgamation, gene tree inference, and evolutionary characterization in this study. 2

Supplementary Fig. 2. Changes in number of reads, total nucleotide length, mean read length, and mapping rate by RNA-seq read filtering. 3

Supplementary Fig. 3. A correlation analysis for the detection and removal of anomalous RNA-seq samples. 4

Supplementary Fig. 4. Characteristics of amalgamated transcriptome. 5

Supplementary Fig. 5. Expression of organ-specific marker genes in human and mouse. 14

Supplementary Fig. 6. Comparison of SVA-log-FPKM and SVA-log-TPM in expression regime shift detection. 15

Supplementary Fig. 7. The relationships between expression shifts and chromosomal location. 18

Supplementary Fig. 8. Evaluation of gene set completeness. 19

Supplementary Fig. 9. Alternative analysis supporting non-linear change in protein evolution rate in correlation with expression regime shifts. 20

Supplementary Fig. 10. The number of expressed genes does not explain the organ-wise abundance of PEO shifts. 21

Supplementary Fig. 11. Evolutionary dynamics of gene expression analyzed with conservative datasets. 22

Supplementary Fig. 12. Effects of phylogeny reconciliation. 23

Supplementary Fig. 13. Gene tree reconstruction. 25

Supplementary Fig. 14. Gene tree skimming in phylogenetic comparative analysis. 27

Supplementary References 28

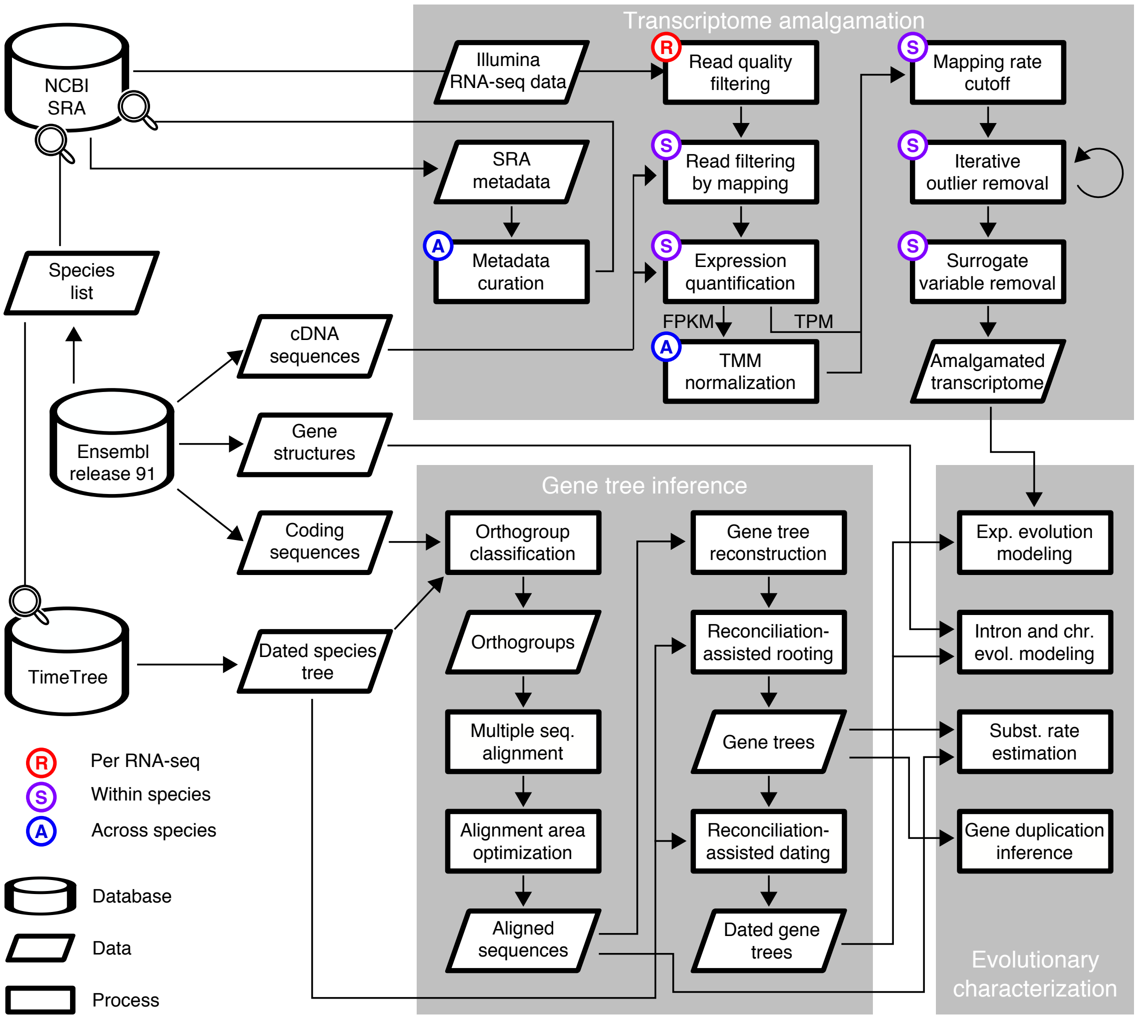

Supplementary Fig. 1. A flow-chart of transcriptome amalgamation, gene tree inference, and evolutionary characterization in this study.

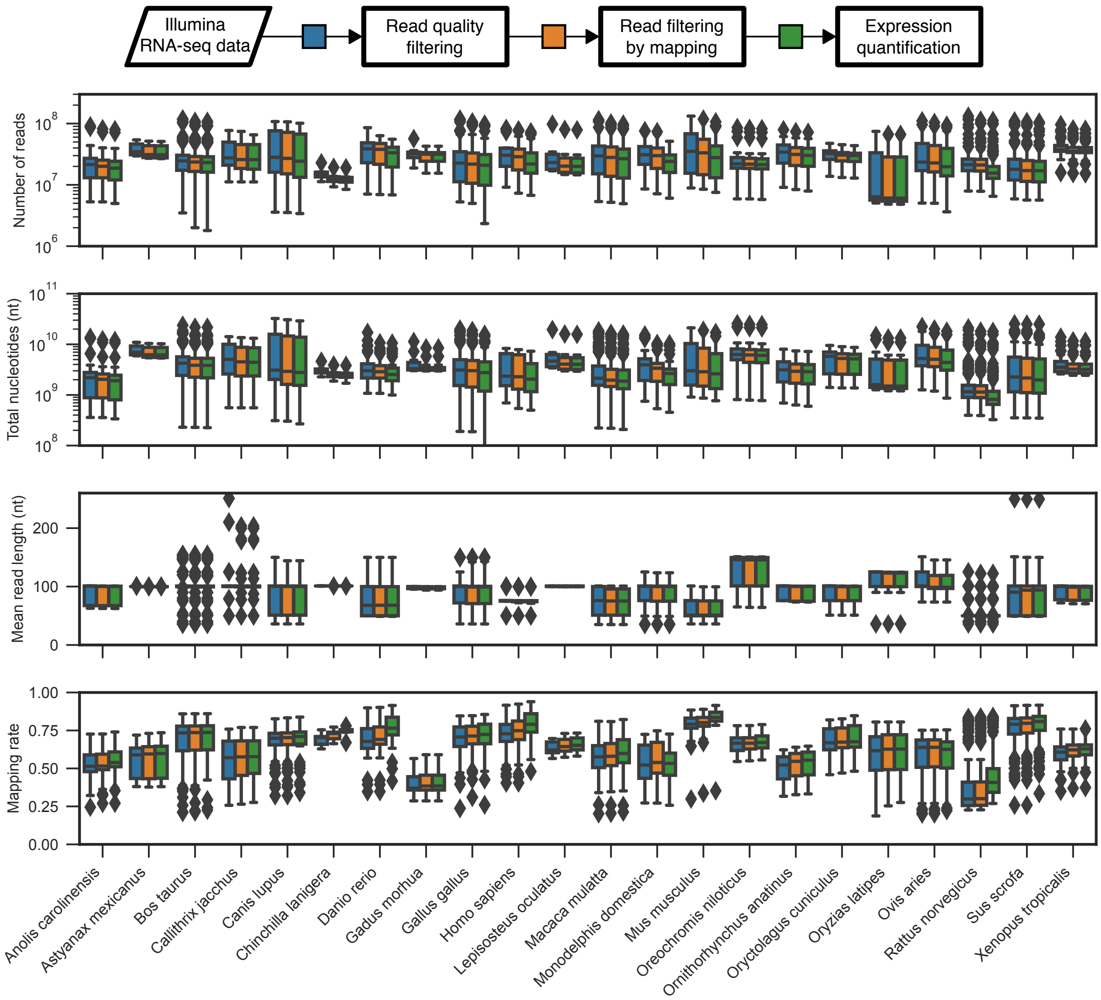

Supplementary Fig. 2. Changes in number of reads, total nucleotide length, mean read length, and mapping rate by RNA-seq read filtering. Mapping rates were, in almost every case, improved by both read quality filtering and read filtering by mapping to miscellaneous genomic features. Box plot elements are defined as follows: center line, median; box limits, upper and lower quartiles; whiskers, 1.5 × interquartile range; points, outliers.

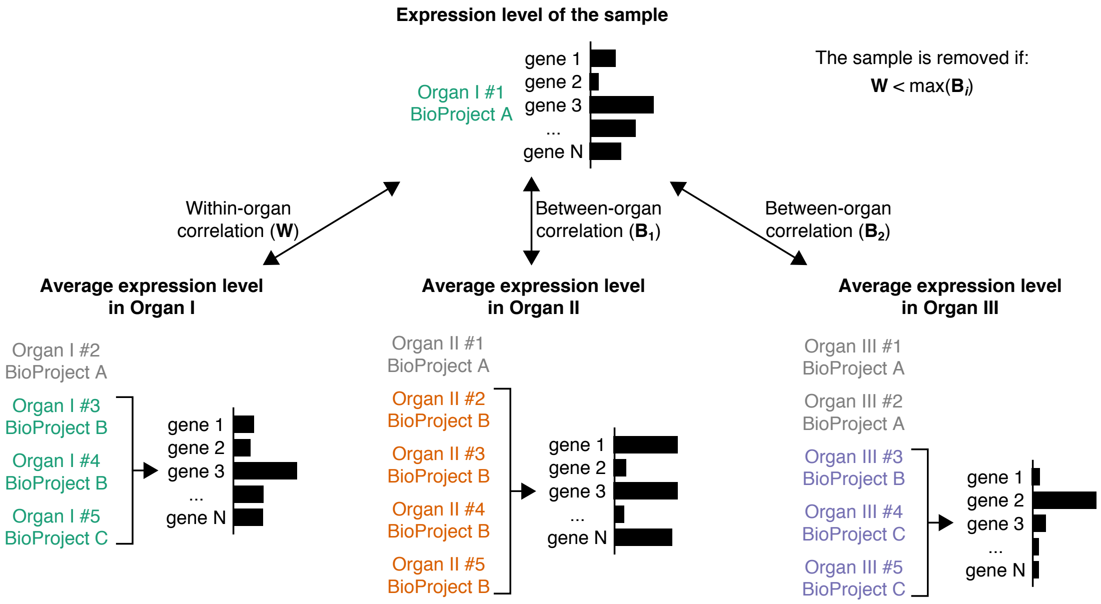

Supplementary Fig. 3. A correlation analysis for the detection and removal of anomalous RNA-seq samples. Expression levels of all genes were compared between the sample and the organ averages. A sample was removed if any between-organ comparisons yielded a correlation coefficient higher than the within-organ comparison.

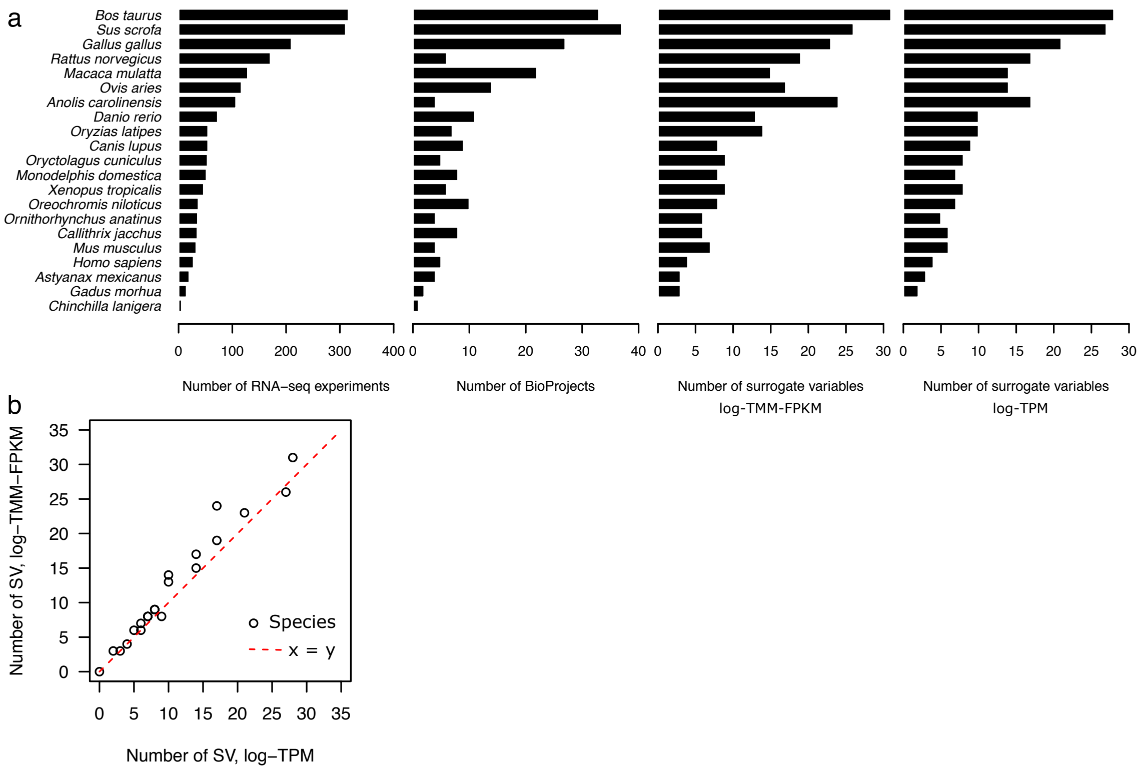

Supplementary Fig. 4. Characteristics of amalgamated transcriptome. (a) The number of RNA-seq experiments, BioProjects, and detected surrogate variables in each species. The counts were derived from the final dataset. The numbers of surrogate variables (SV) are correlated with the numbers of RNA-seq samples and BioProjects. (**b**) Relationships of the number of SVs with log-TPM and log-TMM-FPKM. Points correspond to species. (**c–d**) Correlation heatmaps of corrected transcriptomes. See Supplementary Data 1 for full descriptions including RNA-seq sample IDs and BioProject IDs. (**e–f**) Distinct distributions of Pearson's correlation coefficients depending on whether a pair of RNA-seq samples are the same organ or whether they are from the same research project. (**g–h**) Predictor analysis of detected surrogate variables. The predictive power was analyzed by linear regression using different properties of RNA-seq experiments: organ (brain, heart, kidney, liver, ovary, and testis), BioProject (e.g., PRJNA176589), library selection (e.g., cDNA and polyA), library layout (single and paired), instrument (e.g., Illumina HiSeq 2500 and NextSeq 550), number of read (e.g., 91,641,467 reads), % lost, fastp (percentage of reads that are removed by fastp; e.g., 5%), % lost, misc feature (percentage of reads that are mapped to non-nuclear-mRNA features and are removed from the analysis; e.g., 5%), minimum read length (e.g., 25 nt), average read length (e.g., 70 nt), maximum read length (e.g., 75 nt), and mapping rate (e.g., 80%). The predictors are summarized in Supplementary Data 1. (**i–j**) Multispecies correlation analysis of averaged organ expression. Corrected expression levels of 1,377 single-copy orthologs were used to calculate pairwise Pearson's correlation coefficients. (**k**) A principal component analysis using expression levels of 1,377 single-copy orthologs from 21 species. Points correspond to RNA-seq samples. Curves show the estimated kernel density. Explained variations in percentages are indicated in each axis. (**l**) Estimated organ-wise expression levels of a housekeeping gene. Since data from relatively many BioProjects are available, glyceraldehyde-3-phosphate dehydrogenase gene (GAPDH, ENSGALG00000014442) in *Gallus gallus* is shown. Points correspond to the average expression level calculated by a random subsampling. All data points and the median value (bar), rather than a box plot, are shown if the number of subsampled BioProject combinations is less than 10. Box plot elements are defined as follows: center line, median; box limits, upper and lower quartiles; whiskers, 1.5 × interquartile range; points, outliers. A part of animal silhouettes were obtained from PhyloPic (http://phylopic.org). The silhouettes of *Astyanax mexicanus* and *Oreochromis niloticus* are licensed under CC BY-NC-SA 3.0 (https://creativecommons.org/licenses/by-nc-sa/3.0/) by Milton Tan (reproduced with permission), and those of *Anolis carolinensis* (by Sarah Werning), *Ornithorhynchus anatinus* (by Sarah Werning), and *Rattus norvegicus* (by Rebecca Groom; with modification) are licensed under CC BY 3.0 (https://creativecommons.org/licenses/by/3.0/).

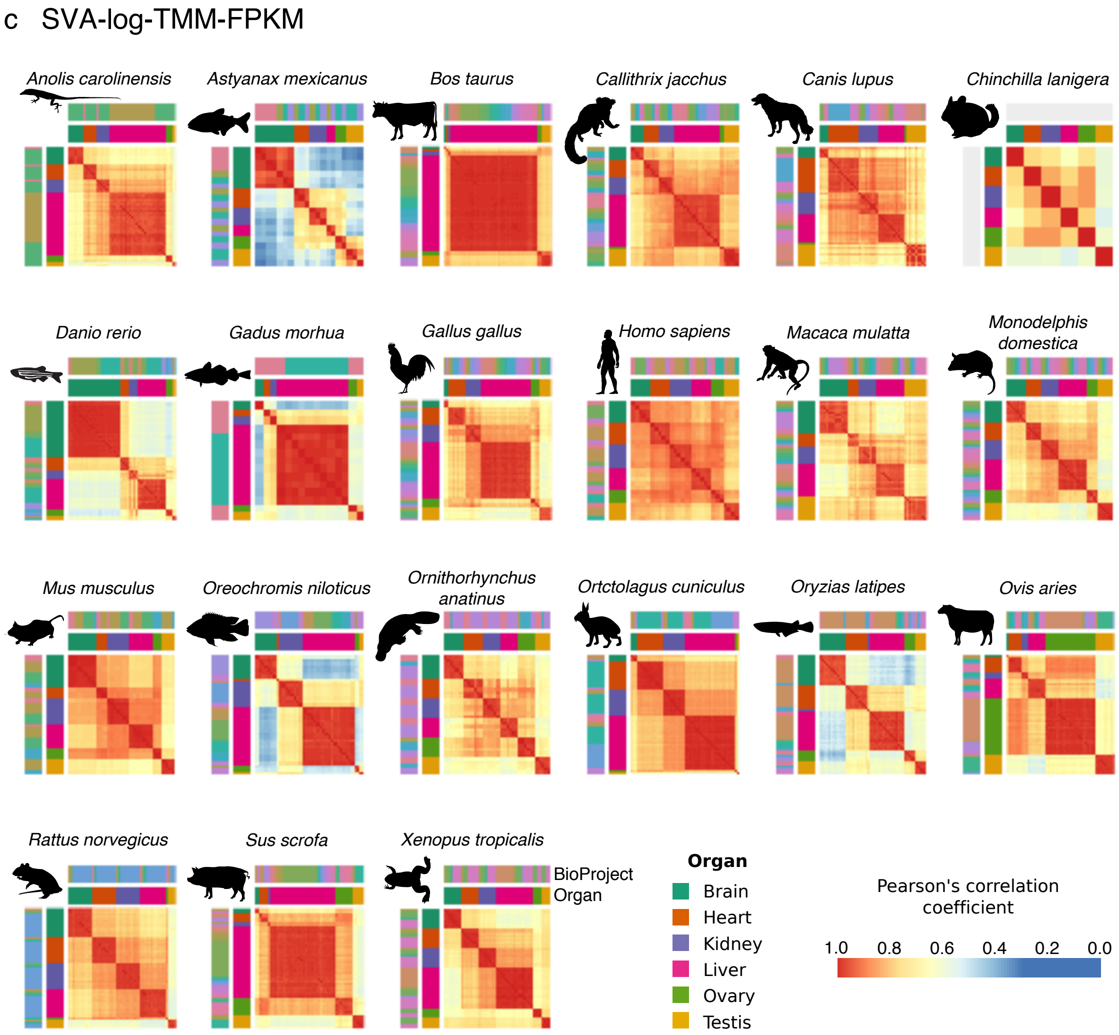

Supplementary Fig. 4 (continued)

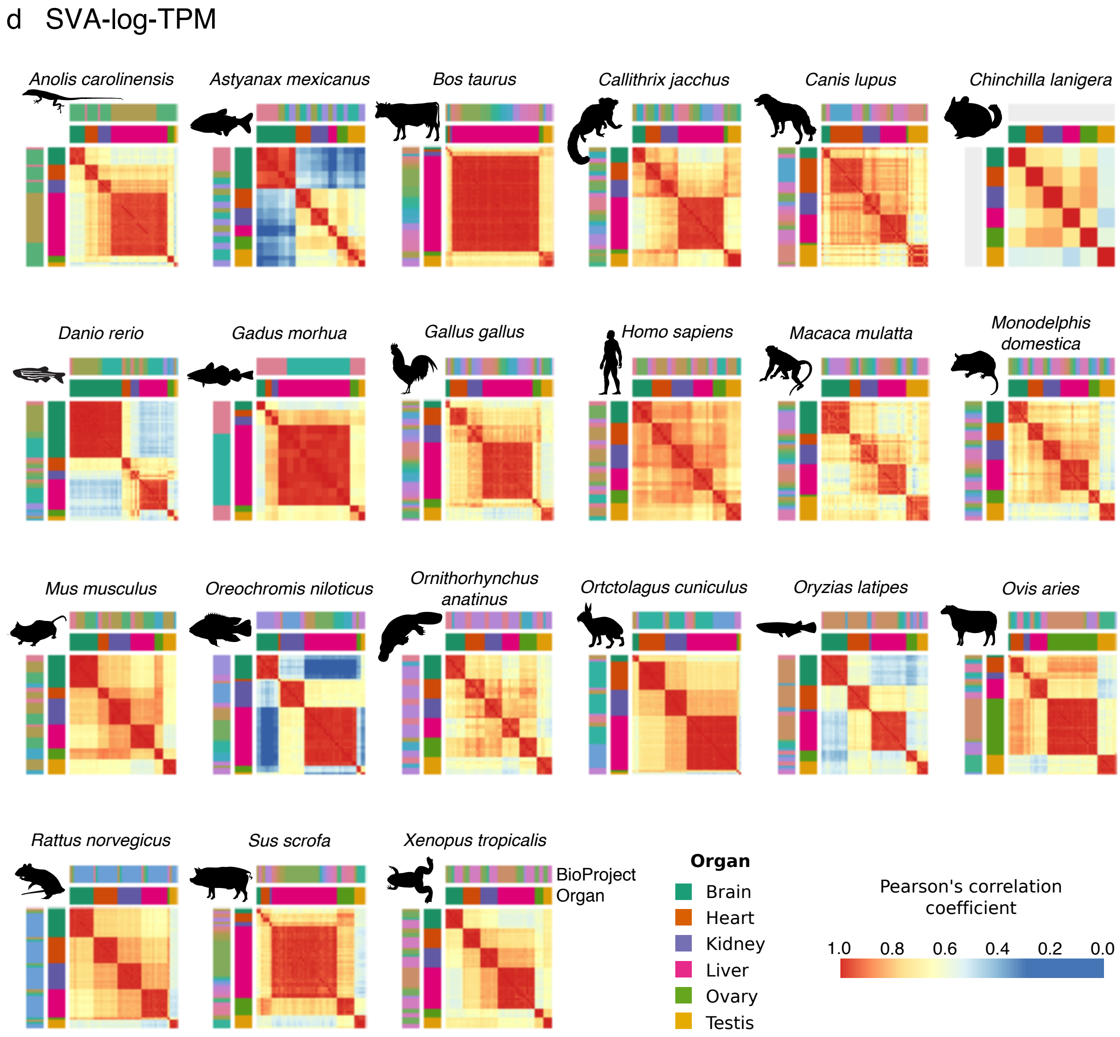

Supplementary Fig. 4 (continued)

e SVA-log-TMM-FPKM

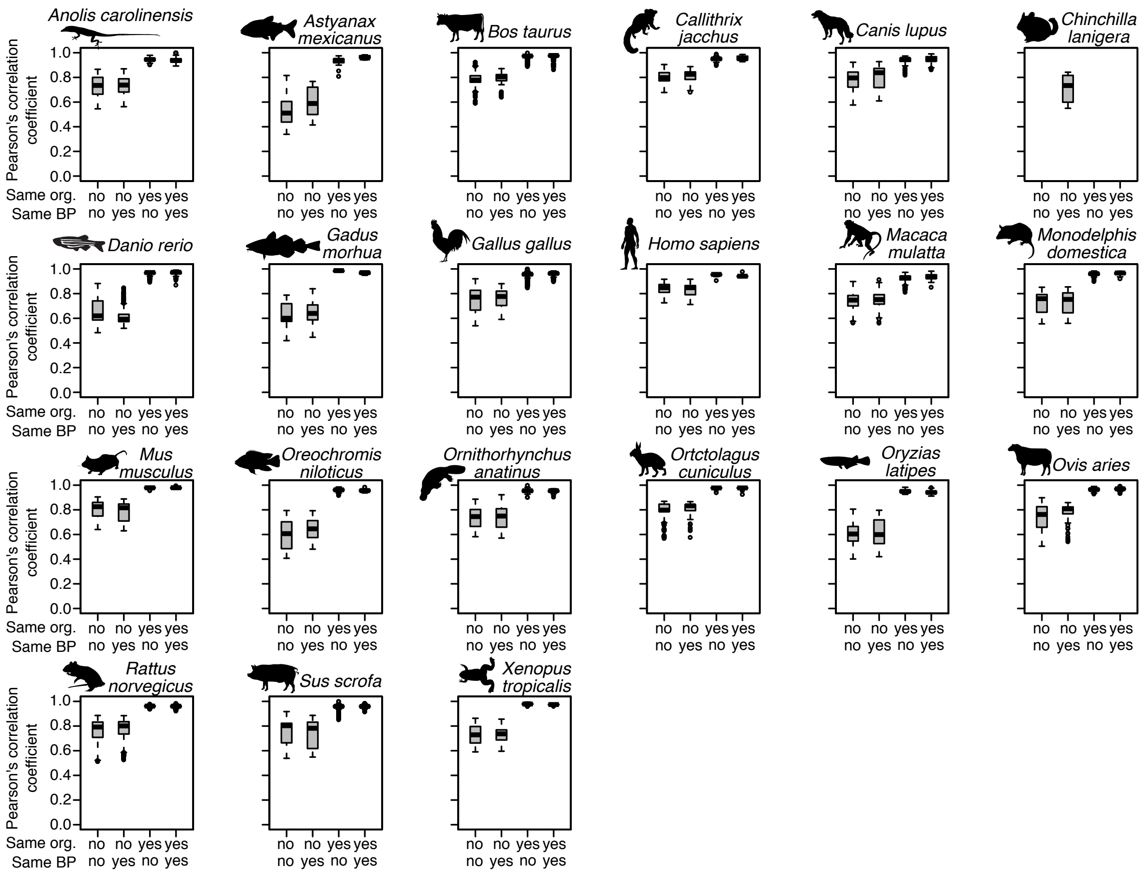

Supplementary Fig. 4 (continued)

f SVA-log-TPM

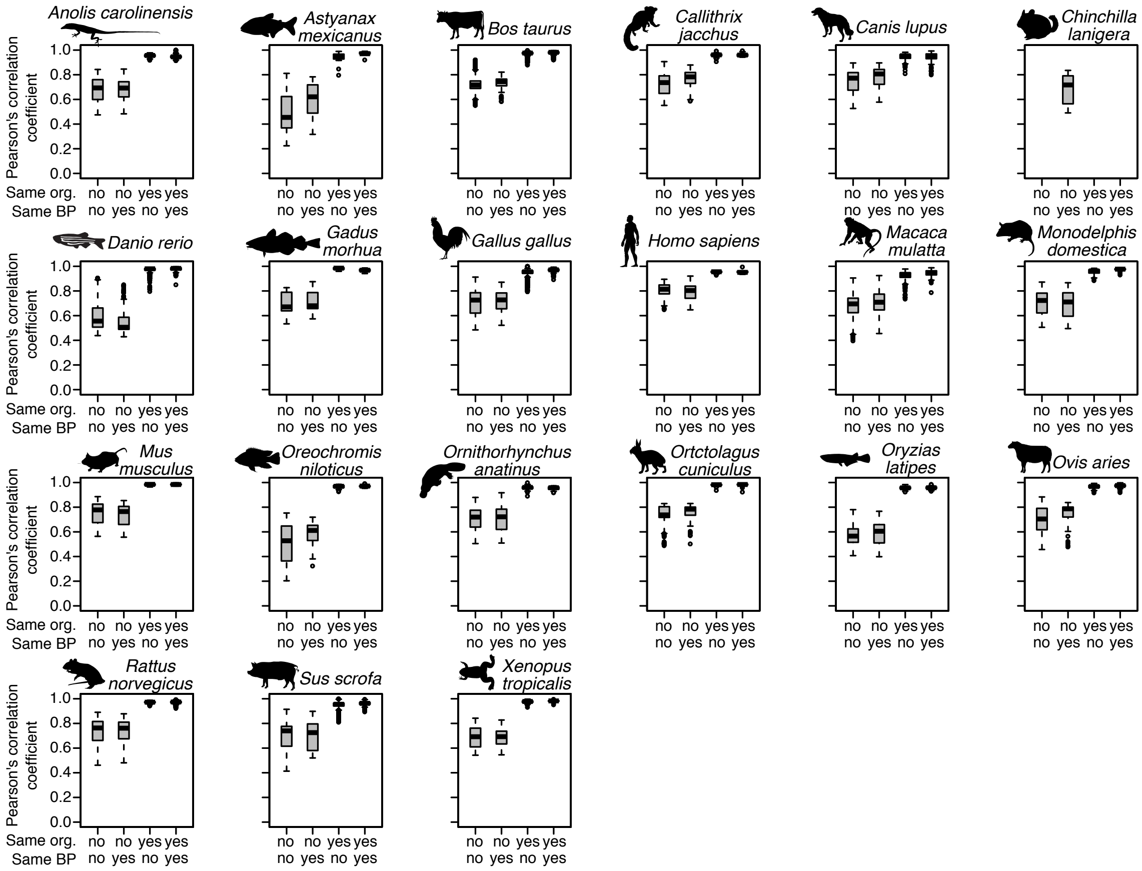

Supplementary Fig. 4 (continued)

g SVA-log-TMM-FPKM

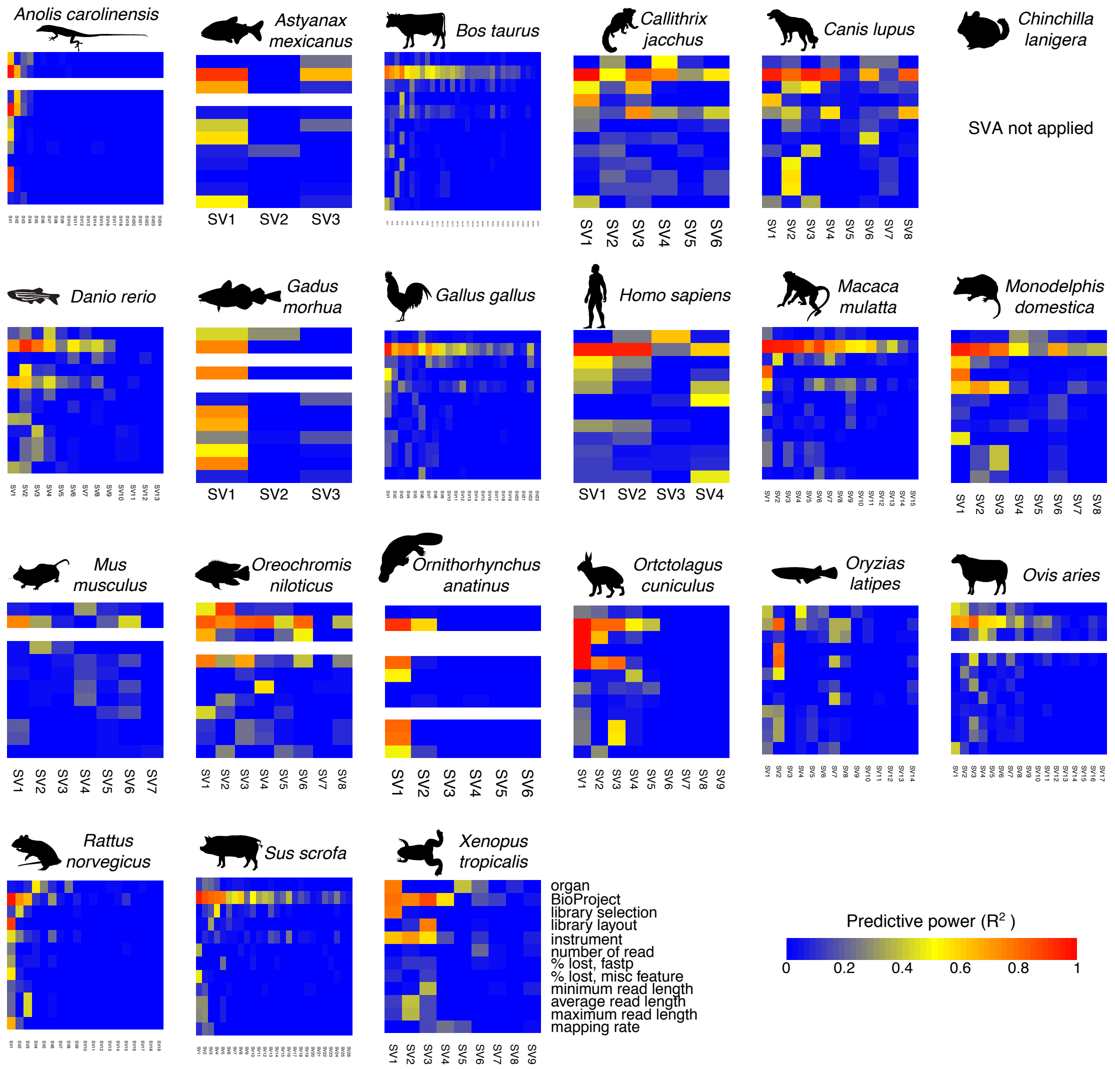

Supplementary Fig. 4 (continued)

h SVA-log-TPM

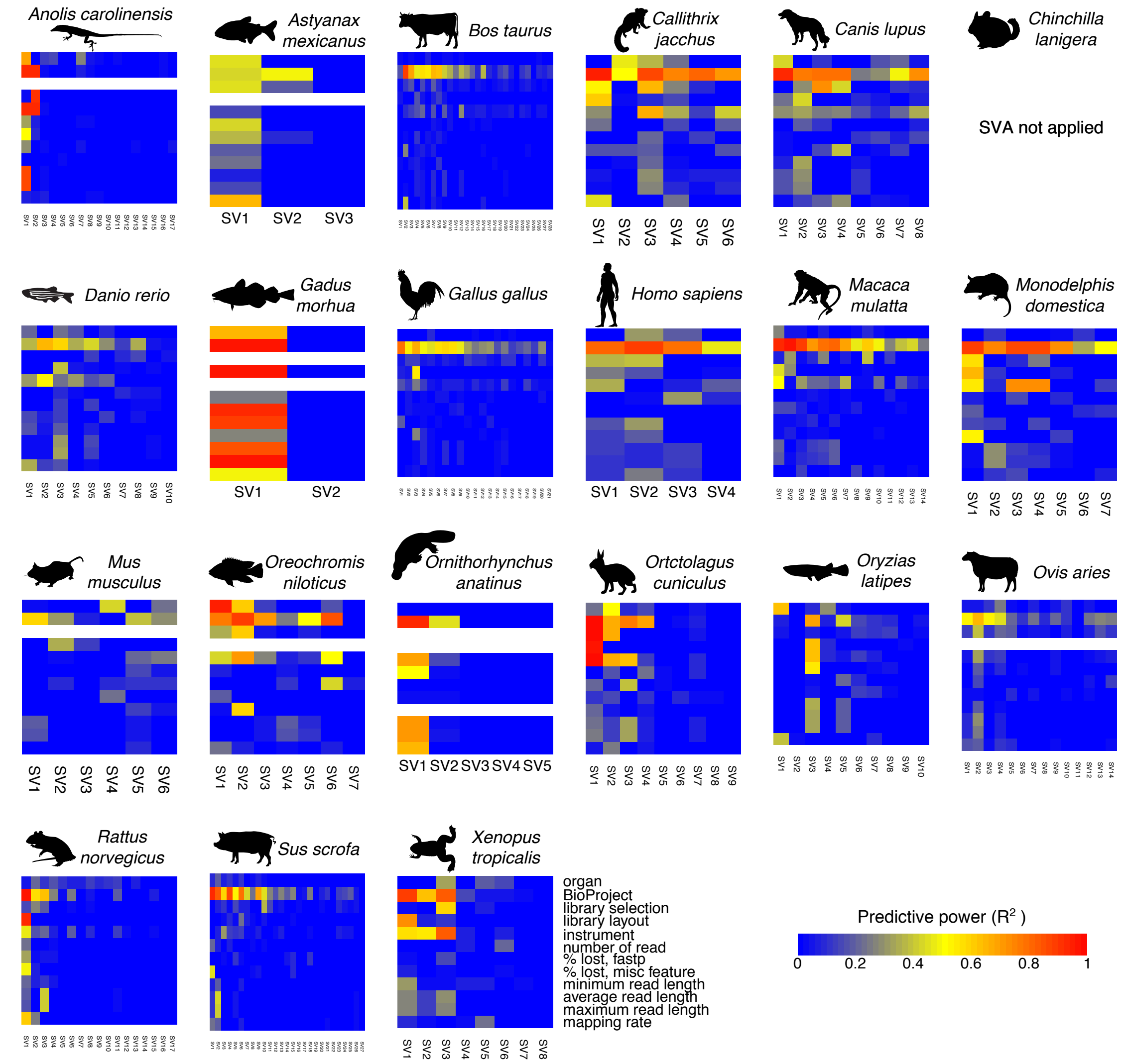

Supplementary Fig. 4 (continued)

i SVA-log-TMM-FPKM

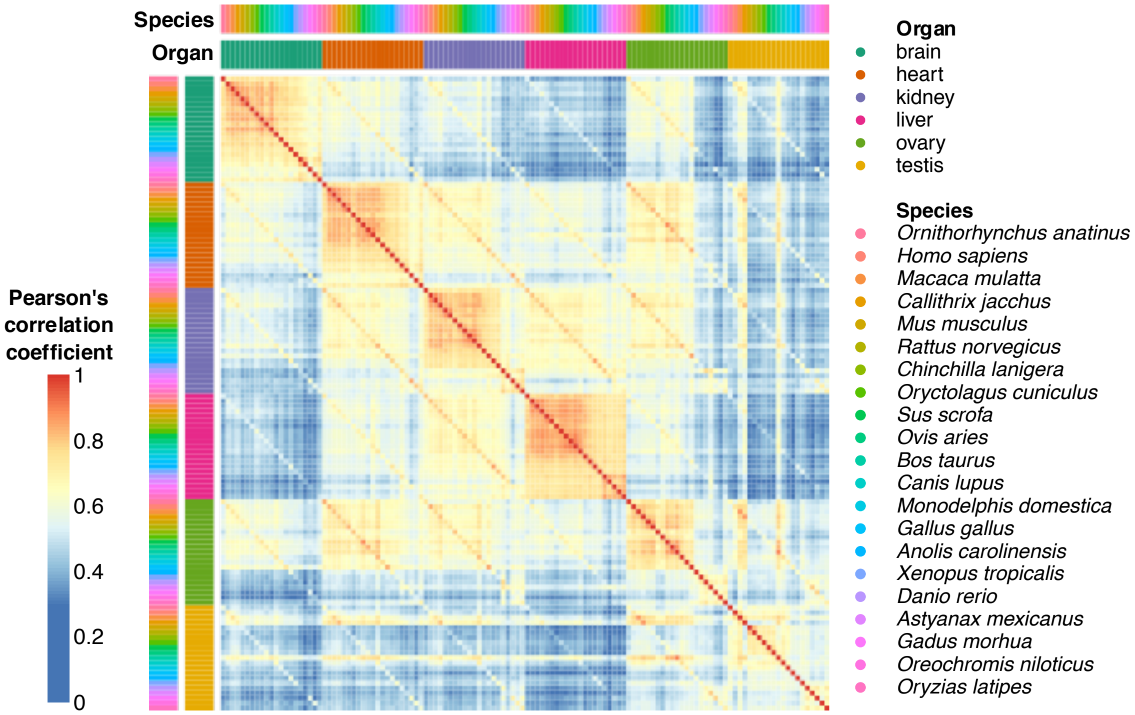

j SVA-log-TPM

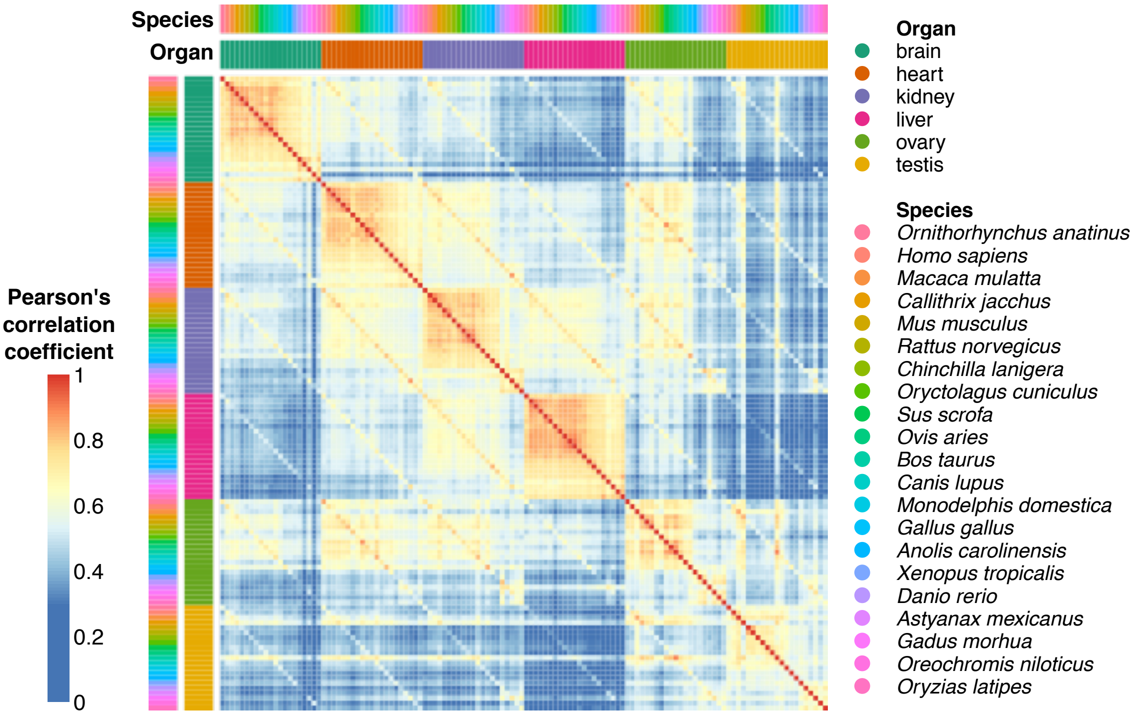

Supplementary Fig. 4 (continued)

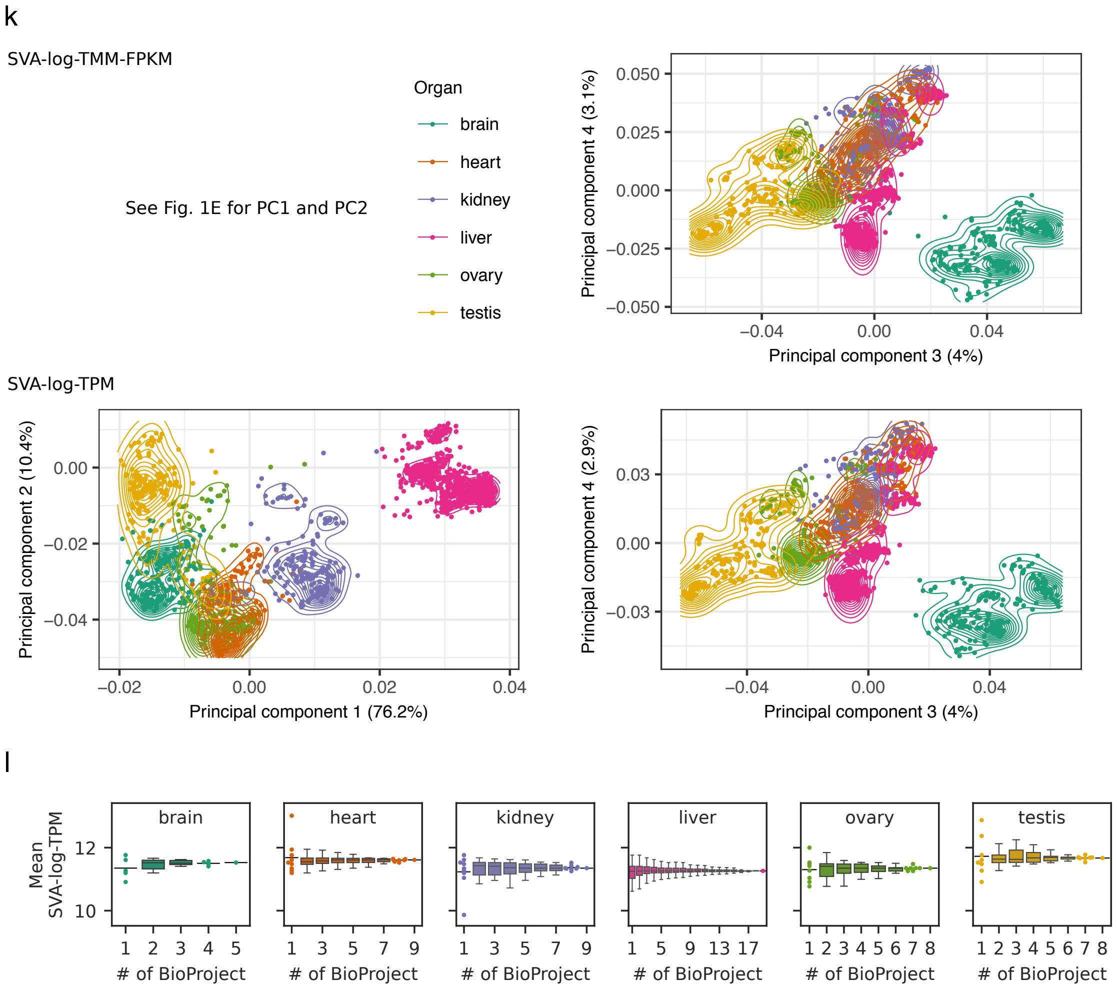

Supplementary Fig. 4 (continued)

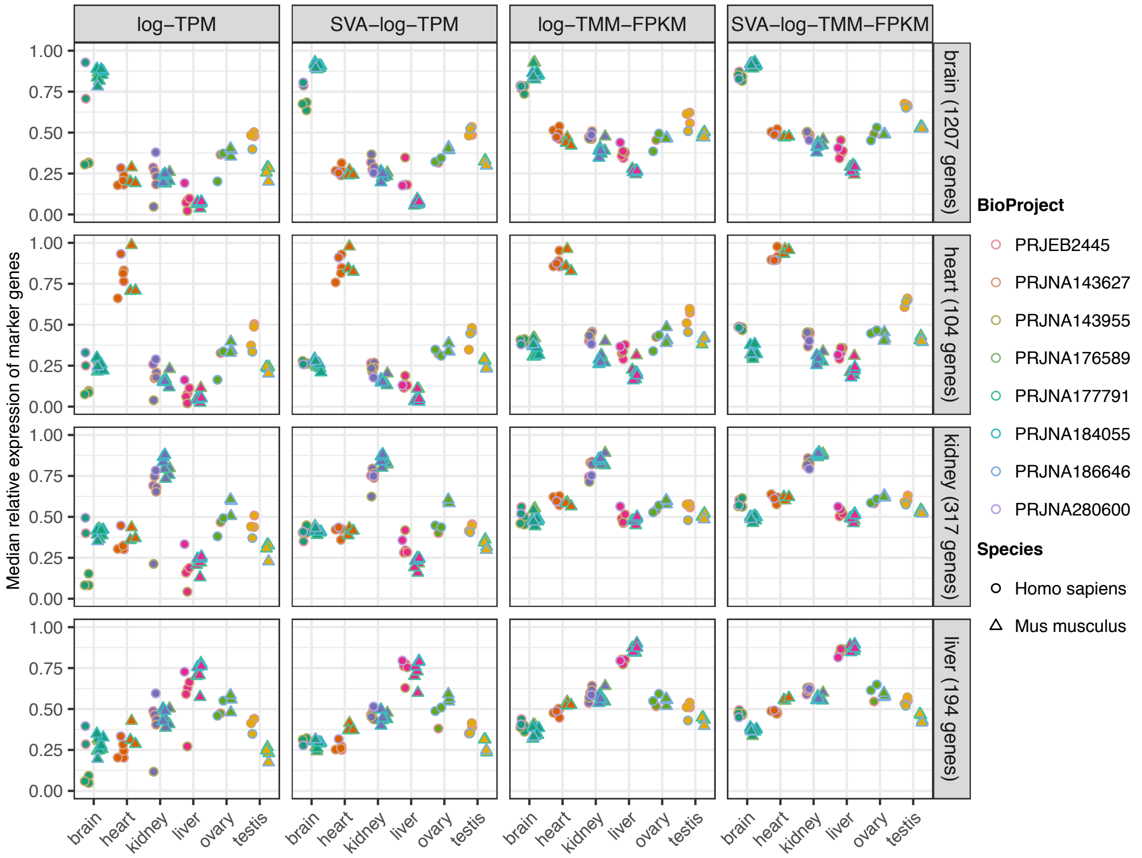

Supplementary Fig. 5. Expression of organ-specific marker genes in human and mouse. Marker genes were retrieved from PanglaoDB[1], and its median expression values were obtained for each RNA-seq sample. A cell-type-wise analysis is provided in Supplementary Dataset[2]. Cell types in ovary and testis were not included in PanglaoDB (access date: April 1, 2020).

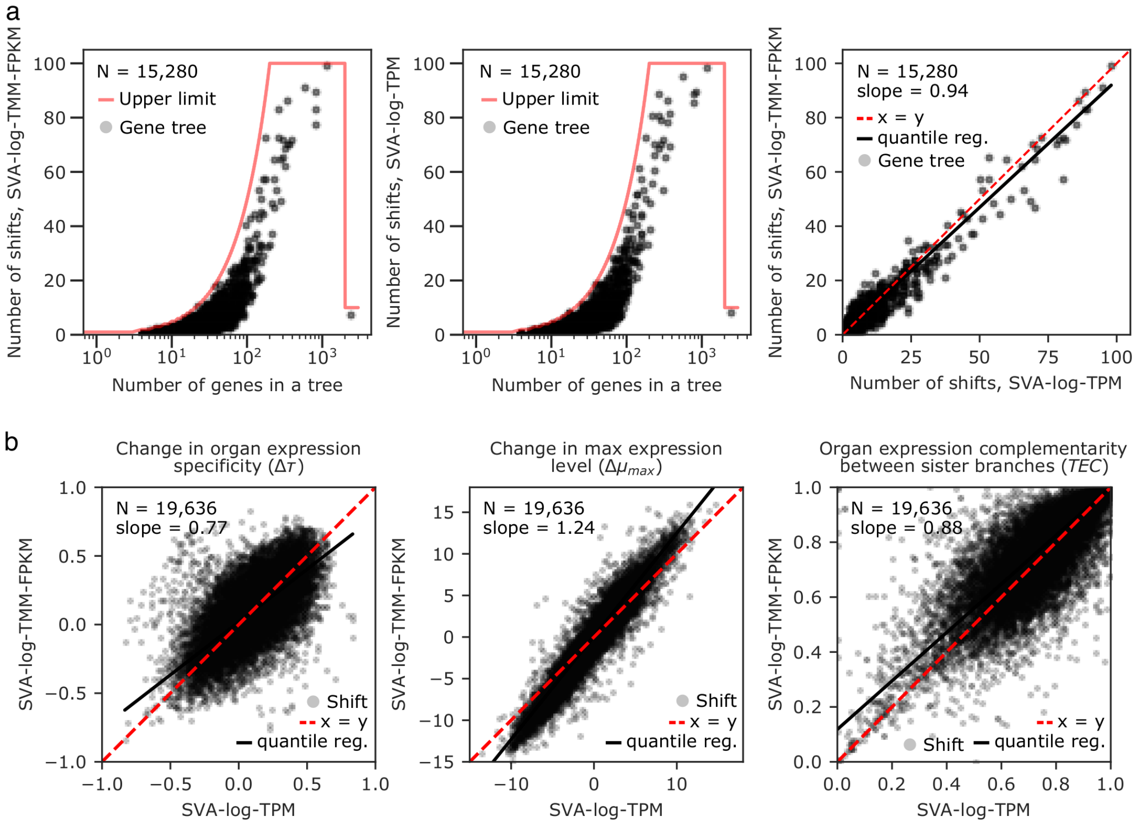

Supplementary Fig. 6. Comparison of SVA-log-FPKM and SVA-log-TPM in expression regime shift detection. (**a**) The numbers of expression regime shifts. Each point corresponds to a gene tree. The solid red lines show the upper limits in the regime shift search. The numbers of shifts detected with SVA-log-TMM-FPKM (left) and SVA-log-TMM (center) are well correlated (right). The black line is a quantile regression, and the dashed red line shows a slope of 1. (**b**) Expression properties. Each point corresponds to an expression regime shift which is consistently detected by SVA-log-TMM-FPKM and SVA-log-TPM. (**c–d**) The branch-wise numbers of detected shifts in the species tree. The shifts were categorized by primary-expressed organs (PEOs) in ancestral and derived states. The order of the species tree branches (y axis) corresponds to the total number of corresponding gene tree branches in the dataset. See Fig. 3a for branch IDs.

c SVA-log-TMM-FPKM

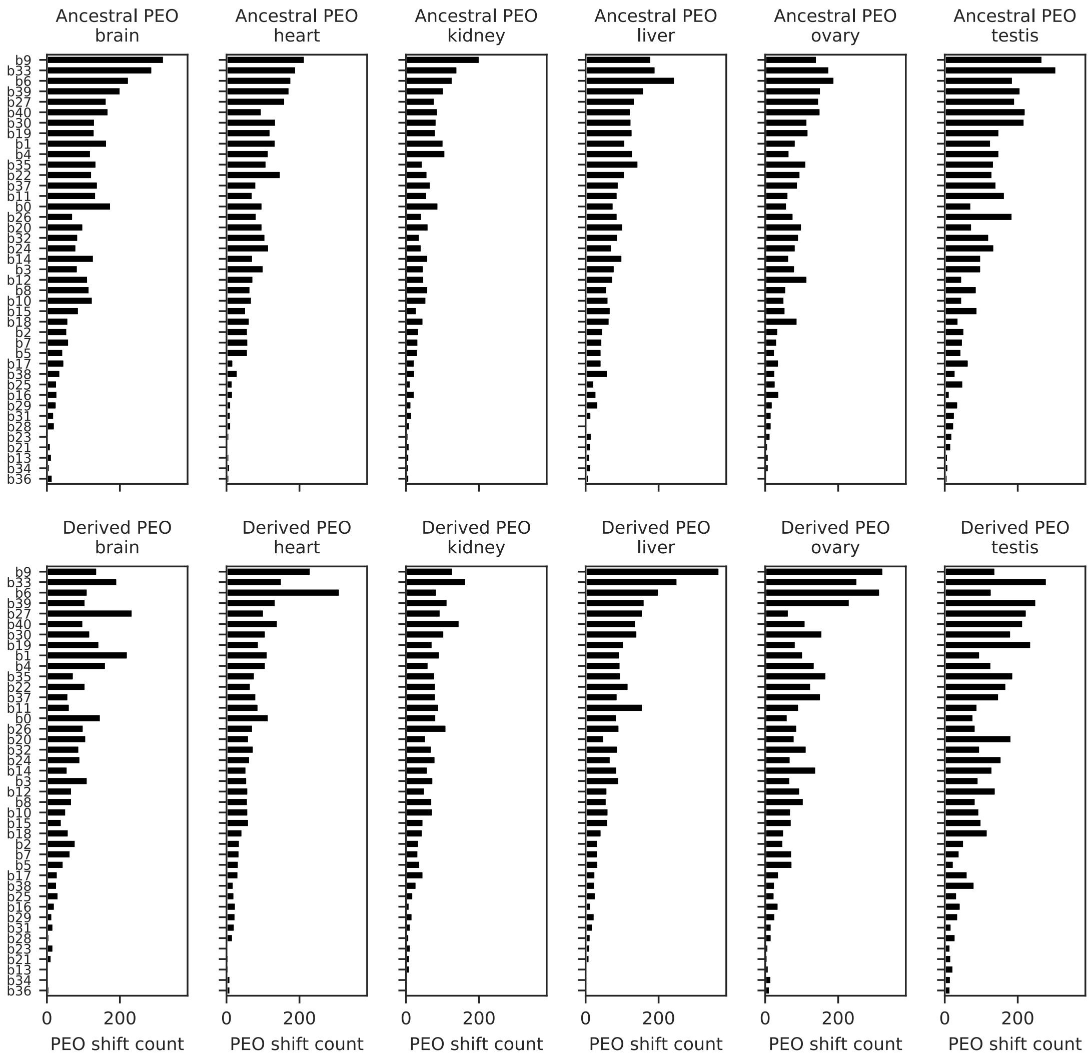

Supplementary Fig. 6 (continued)

d SVA-log-TPM

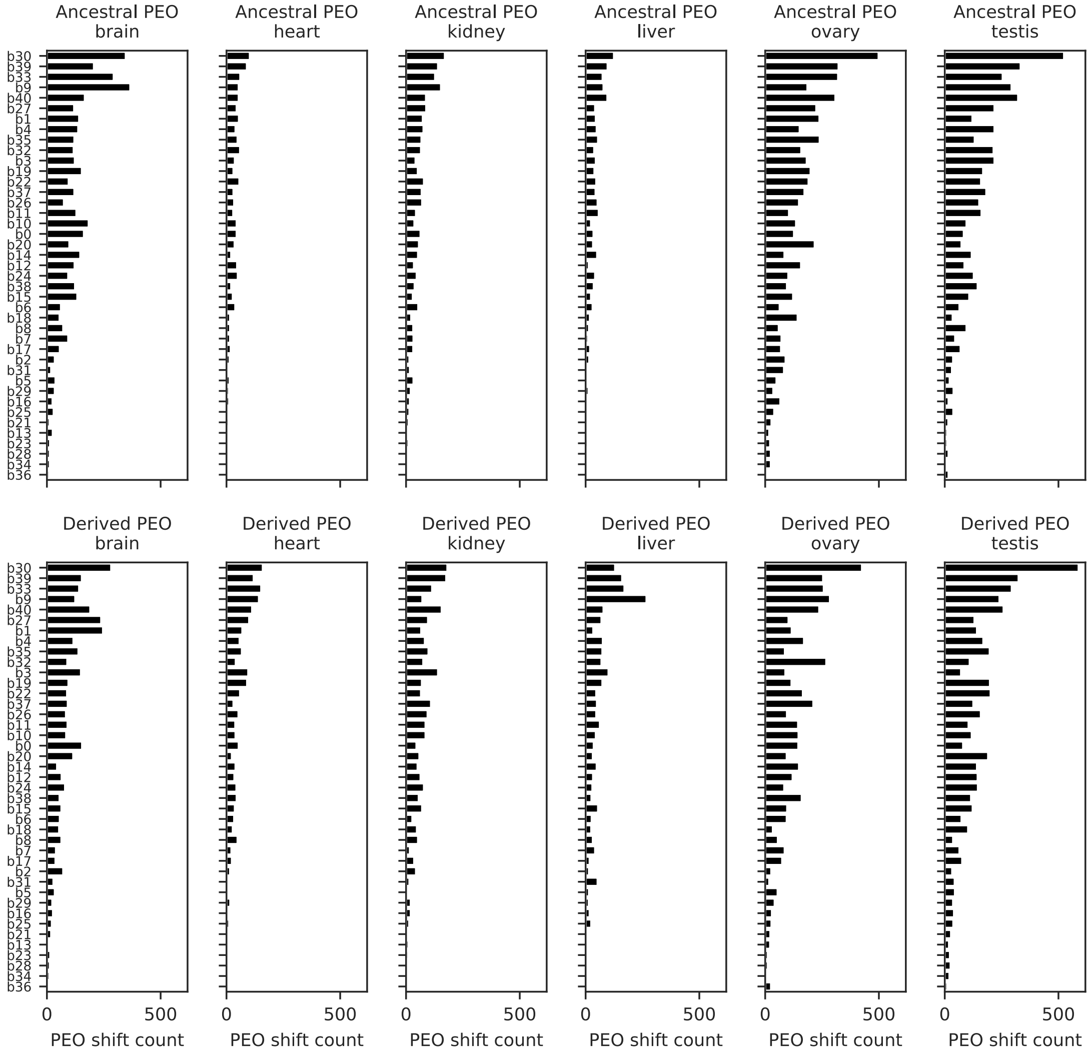

Supplementary Fig. 6 (continued)

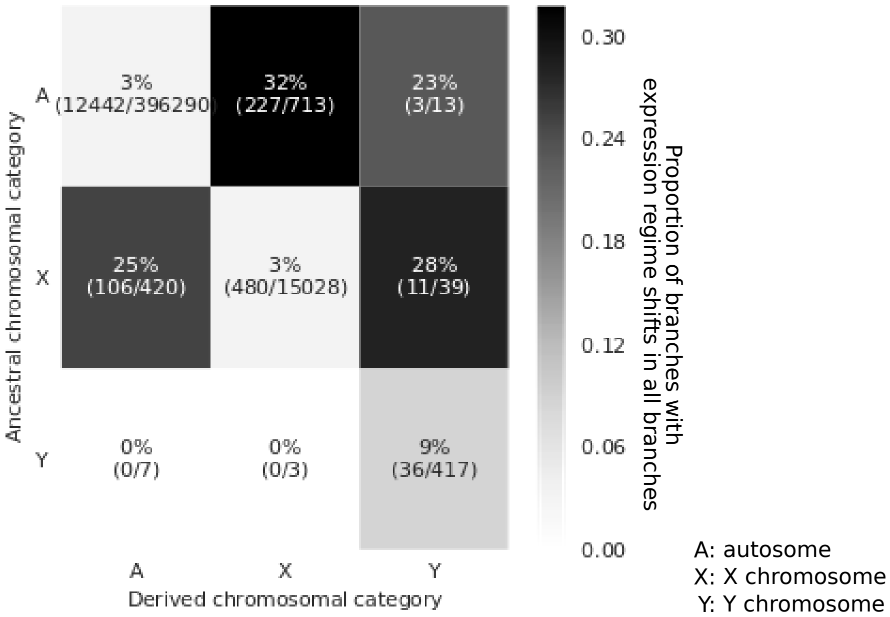

Supplementary Fig. 7. The relationships between expression shifts and chromosomal location. The heatmap shows the frequency of expression shifts observed among the branches with or without a change in the chromosomal category (non-diagonal or diagonal, respectively). Chromosomal locations were categorized into autosomes (A), X chromosome (X) and Y chromosome (Y), and the ancestral locations were inferred by stochastic character mapping.

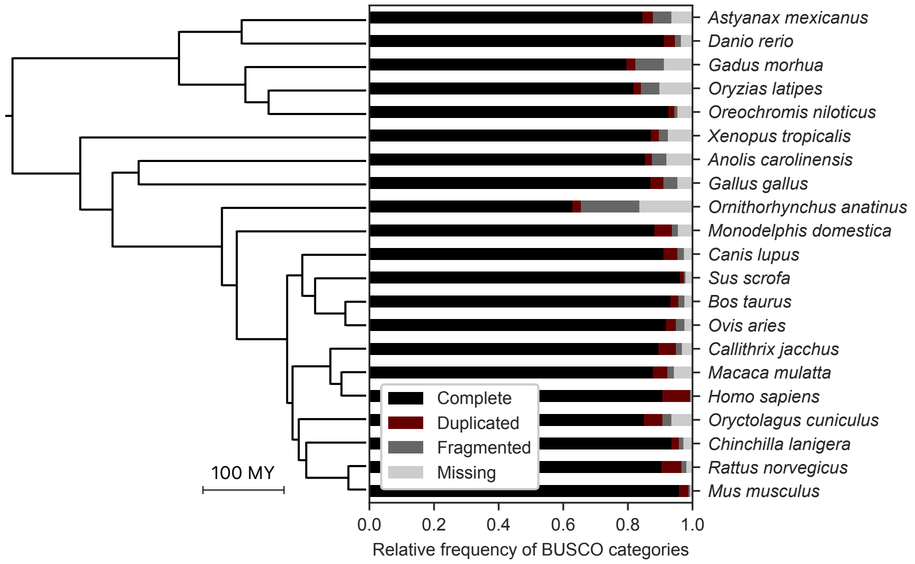

Supplementary Fig. 8. Evaluation of gene set completeness. BUSCO analysis was performed for gene sets from 21 species using 3,407 single-copy orthologs in the dataset "vertebrata_odb10". The species tree is shown to visualize lineage-specific trends.

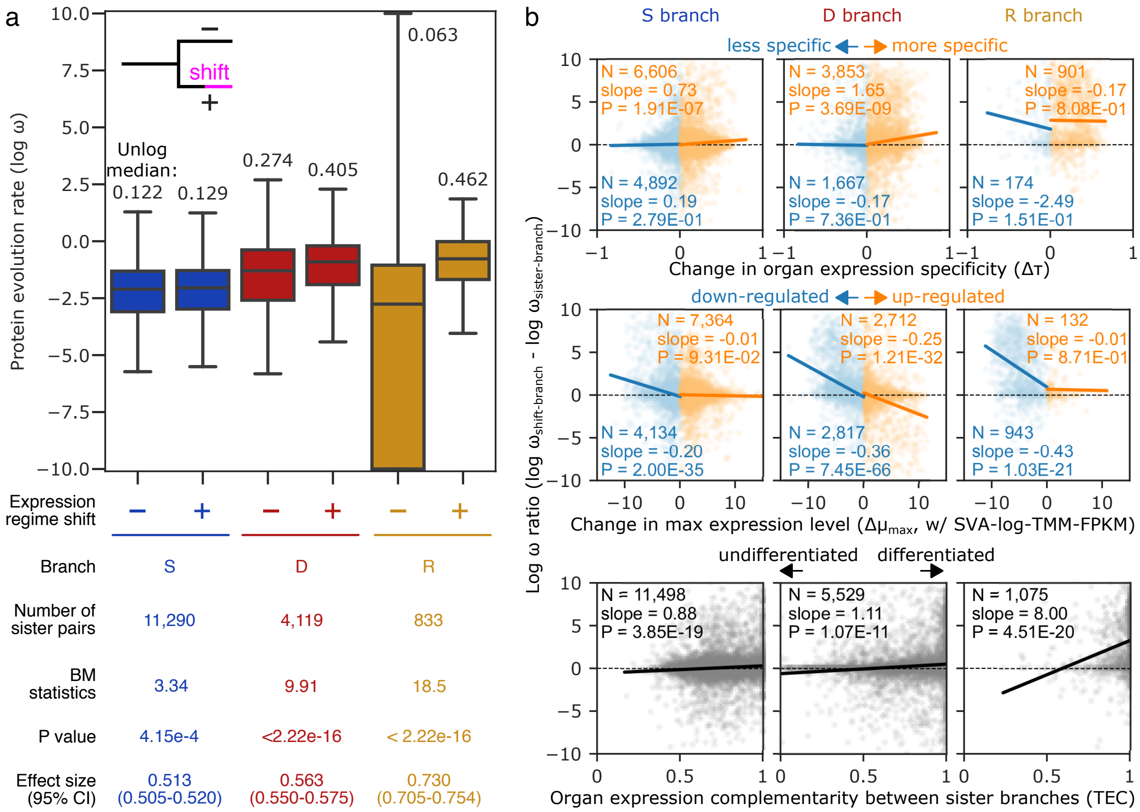

Supplementary Fig. 9. Alternative analysis supporting non-linear change in protein evolution rate in correlation with expression regime shifts. (**a**) Distribution of ω values. While the results from stochastic character mapping (map*dNdS*) are reported in the main text, results from maximum-likelihood estimation (HyPhy) are shown here. A plus (+) indicates branches with expression shifts, whereas minus (−) branches are sisters to the 'plus' branches. Statistical differences between pairs of distributions were tested using a two-sided Brunner–Munzel test[3]. Non-log-transformed median values are shown above the boxplots. For visualization purposes, extreme values exceeding ±10 were clipped. Box plot elements are defined as follows: center line, median; box limits, upper and lower quartiles; whiskers, 1.5 × interquartile range. (**b**) Relationships between protein evolution rate and change in expression properties. While SVA-log-TMM-FPKM-based analysis is reported in the main text, results from SVA-lot-TPM-based analysis is shown here. Stochastic character mapping was used to obtain branch-wise ω values. Points correspond to expression regime shifts (log ω ratio = 0). Dashed lines indicate no between-branch difference in protein evolution rate. Solid lines show a linear regression. Its slope and number of regime shifts are also provided. Regime shifts with negative and positive changes were separately analyzed for organ specificity (upper) and expression level (middle). *P* values indicate whether the slopes were significantly different from zero (two-sided *t* tests).

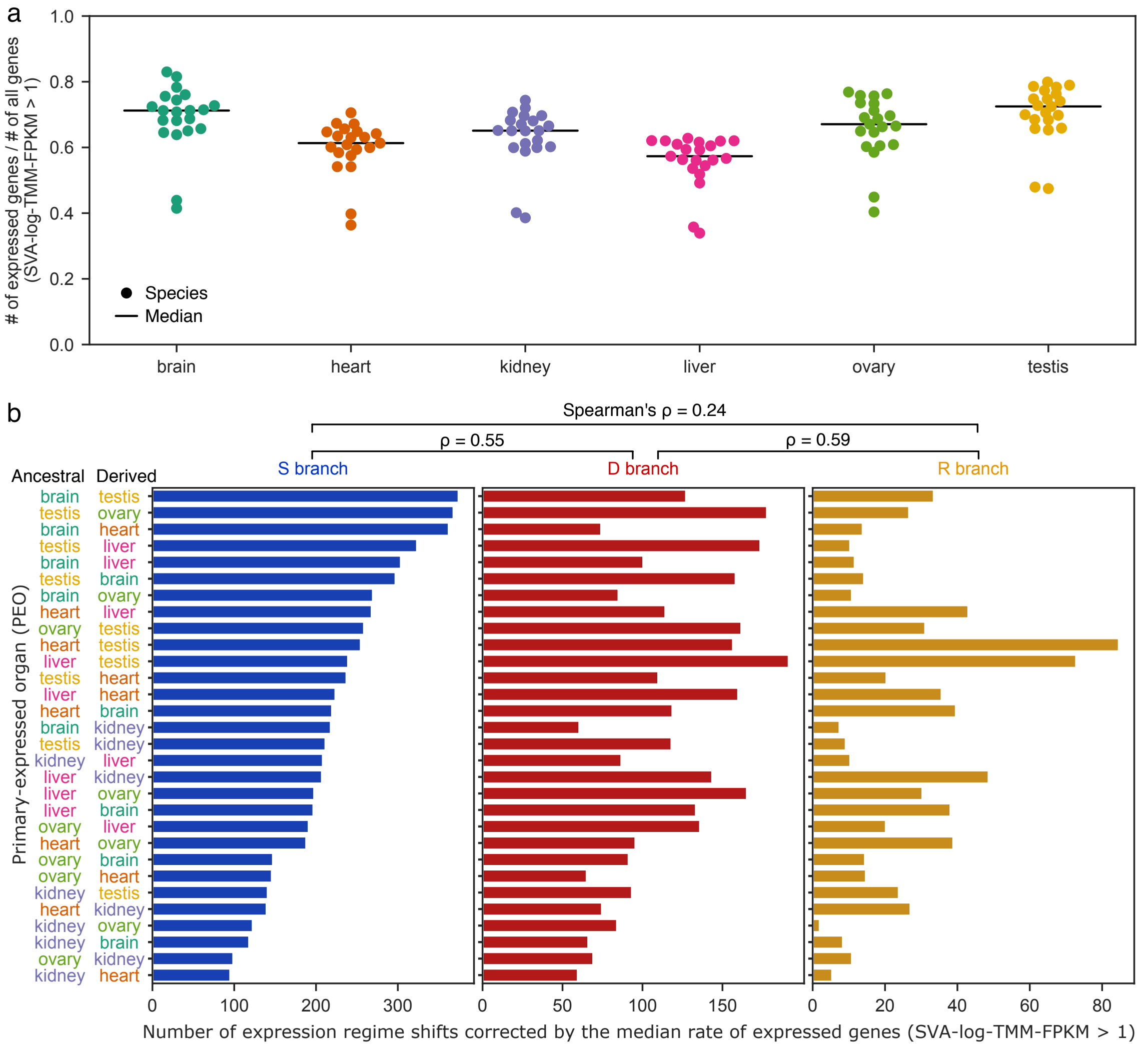

Supplementary Fig. 10. The number of expressed genes does not explain the organ-wise abundance of PEO shifts. (**a**) The organ-wise numbers of expressed genes in the 21 species. In this analysis, expressed genes are defined as genes with >1 SVA-log-TMM-FPKM. (**b**) The PEO shift distributions corrected by the median numbers of expressed genes. The data in Fig. 5a were corrected as follows: $S_{\mathrm{corrected}} = S_{\mathrm{original}} \div (M_{\mathrm{ancestral}}/\bar{M}) \div (M_{\mathrm{derived}}/\bar{M})$. S_{original} corresponds to the numbers shown in Fig. 5a. $M_{\mathrm{ancestral}}$ and M_{derived} are median numbers of expressed genes in ancestral and derived PEOs, respectively. $\bar{M}$ indicates the across-organ average of expressed gene numbers.

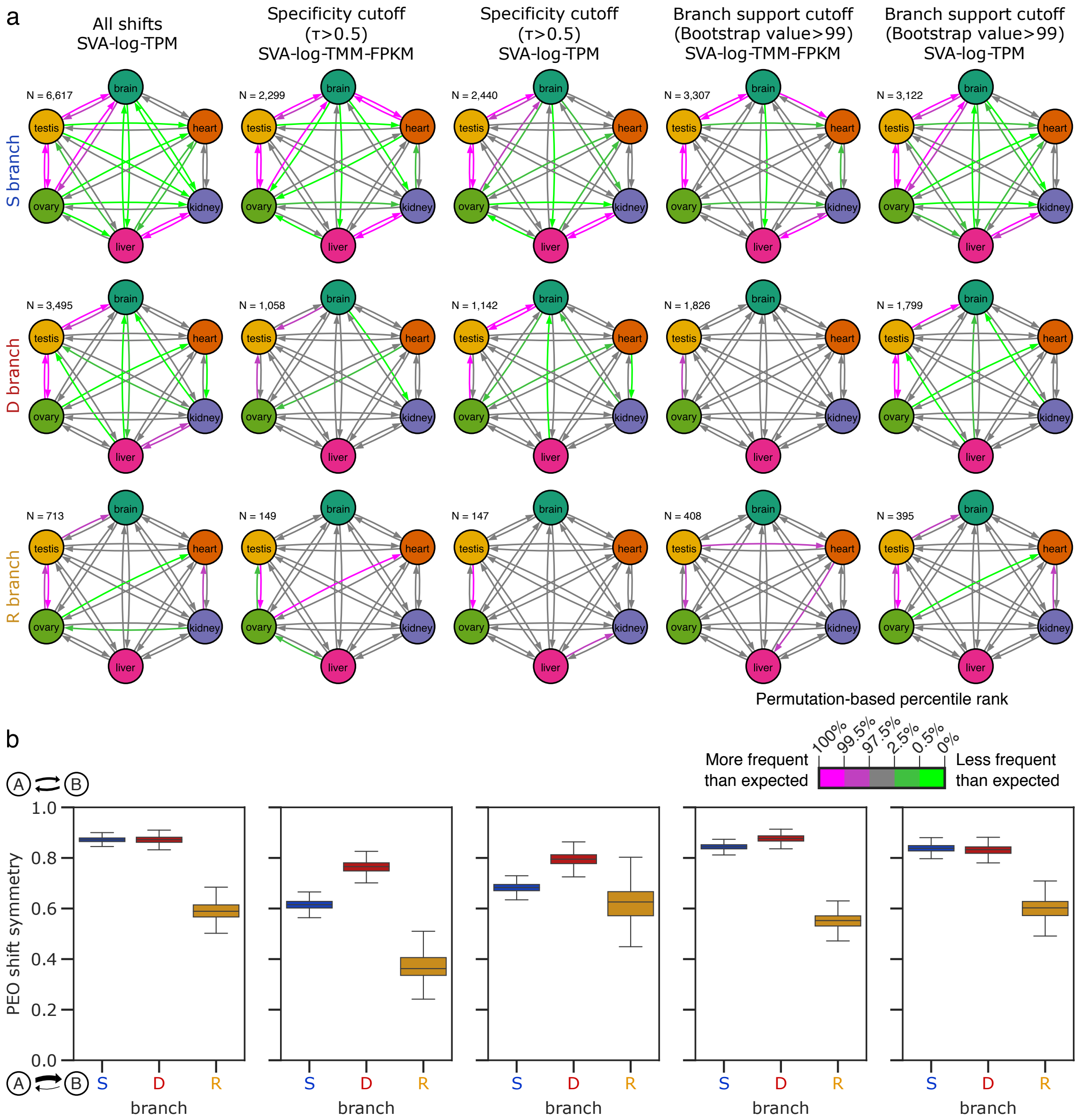

Supplementary Fig. 11. Evolutionary dynamics of gene expression analyzed with conservative datasets. Both SVA-log-TPM and SVA-log-TMM FPKM values were analyzed. Organ-specific genes were examined by selecting PEO shifts with high ancestral and derived organ expression specificity ($\tau > 0.5$). To examine the effect of inference errors in gene tree reconstruction, branches with high support values (ultrafast bootstrap percentage > 99) were analyzed. The panels **a** and **b** correspond to Fig. 5b and c, respectively. Box plot elements are defined as follows: center line, median; box limits, upper and lower quartiles; whiskers, 1.5 × interquartile range.

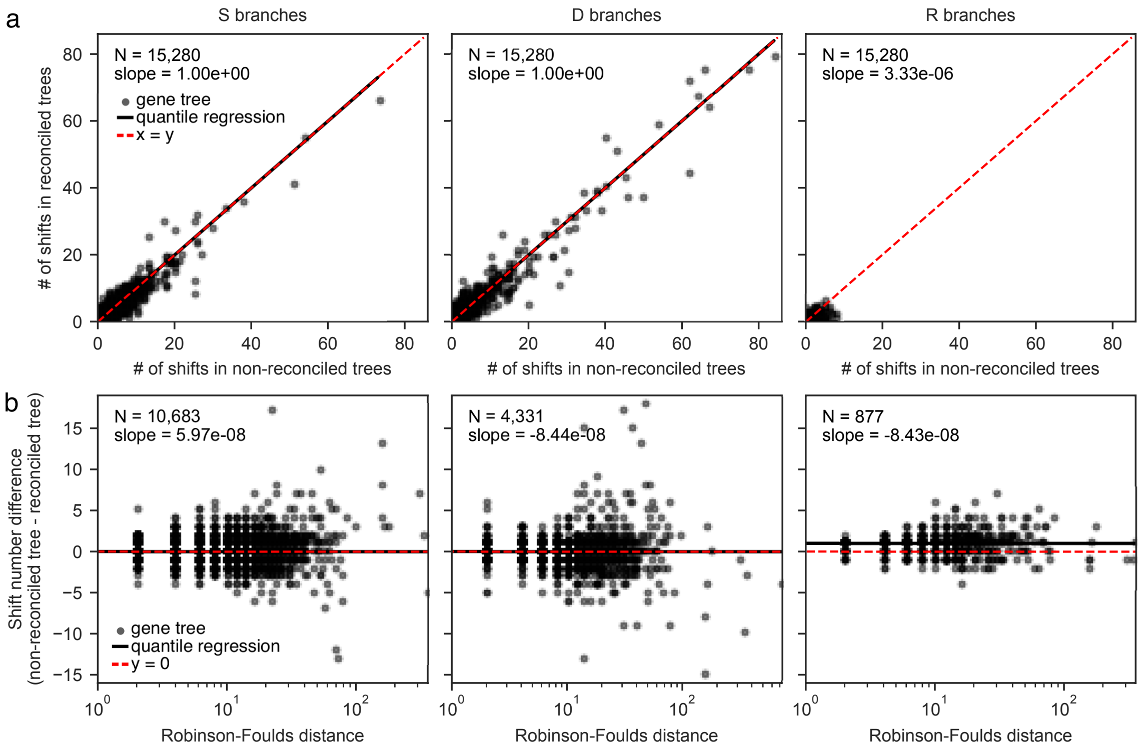

Supplementary Fig. 12. Effects of phylogeny reconciliation. (**a**) The numbers of detected shifts in non-reconciled and reconciled maximum-likelihood gene trees. (**b**) The relationship between shift number difference and tree topology difference measured by Robinson-Foulds distance [51]. Trees with no detected shifts were removed from the analysis. (**c–e**) Reproduction of PEO shift distributions using reconciled gene trees. The panels **c**, **d**, and **e** correspond to Fig. 5a, Fig. 5b, and Fig. 5c, respectively. Box plot elements are defined as follows: center line, median; box limits, upper and lower quartiles; whiskers, 1.5 × interquartile range.

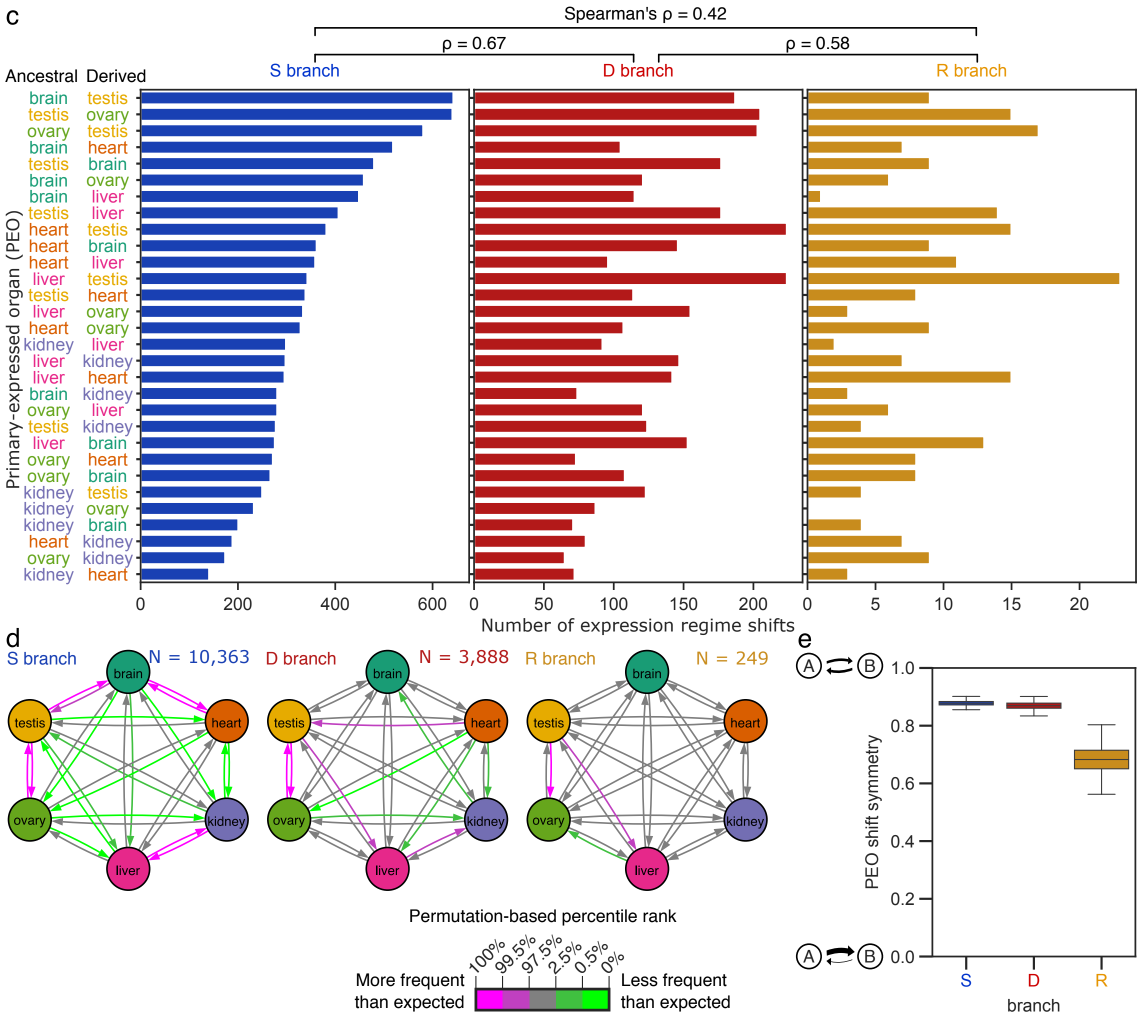

Supplementary Fig. 12 (continued)

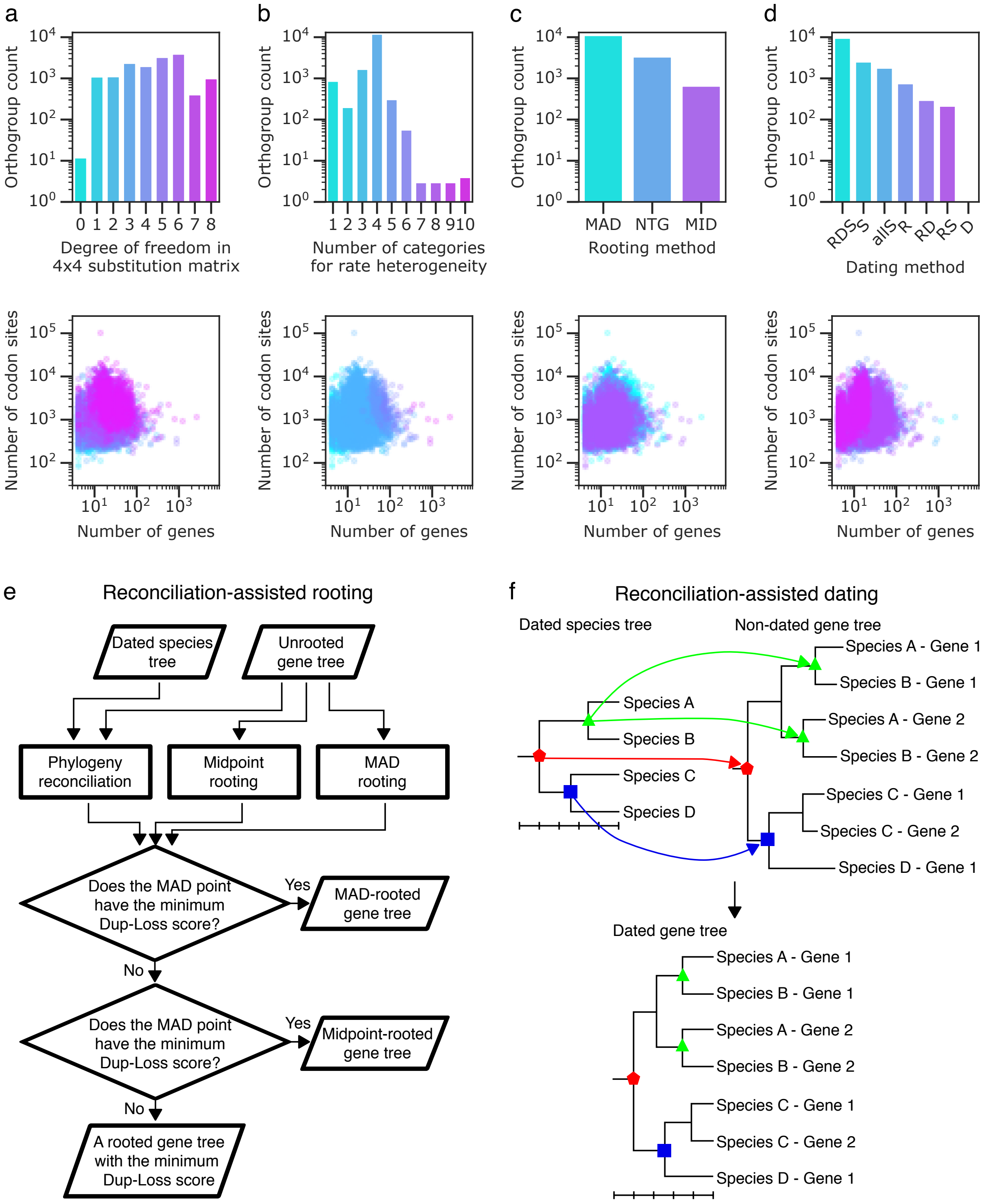

Supplementary Fig. 13. Gene tree reconstruction. (**a**) Complexity of best-fit nucleotide substitution matrices. (**b**) Complexity of rate heterogeneity among nucleotide sites. Both discrete Gamma models[4] and FreeRate models[5,6] were included to count the number of categories for rate heterogeneity. (**c**) Selected rooting positions in reconciliation-assisted gene tree rooting. MAD, minimal ancestor deviation; NTG, 'rooting mode' of NOTUNG; MID, midpoint between the longest path. (**d**) Time-constrained nodes in tree dating. R, root node; S, speciation node; D, duplication node. All available constraints (RDS) are used in the first trial and then successively relaxed if

estimation fails. In the category 'allS', all nodes are speciation nodes and therefore no divergence time estimation was performed for the trees. (**e**) Reconciliation-assisted gene tree rooting. Rooting points were estimated with two different methods: the minimum ancestor deviation (MAD) method and midpoint rooting. If they were compatible with the event parsimony involving gene duplication and loss, the MAD- or midpoint-rooted tree was reported in sequence. If not, one of event parsimony trees was reported. (**f**) Reconciliation-assisted gene tree dating. Speciation nodes in the dated species tree were mapped onto the non-dated gene tree by phylogeny reconciliation. By using those nodes as calibration points, the other node ages were estimated using the penalized likelihood method.

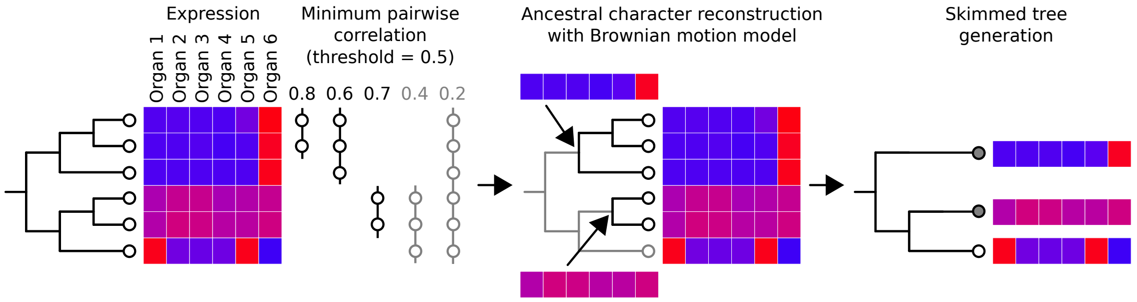

Supplementary Fig. 14. Gene tree skimming in phylogenetic comparative analysis. Gene tree clades are collapsed if character states are highly correlated. Resultant trees contain a smaller number of leaves than the original trees while preserving drastic changes in character evolution. Note that, unlike this example, we used extremely stringent threshold in the analysis (Pearson's correlation coefficient > 0.99).

Supplementary References

1. Franzén, O., Gan, L.-M. & Björkegren, J. L. M. PanglaoDB: a web server for exploration of mouse and human single-cell RNA sequencing data. *Database* **2019**, (2019).
2. Supplementary Dataset. doi:10.17632/3vcstwdbrn.1.
3. Brunner, E. & Munzel, U. The nonparametric Behrens-Fisher problem: asymptotic theory and a small-sample approximation. *Biom. J.* **42**, 17–25 (2000).
4. Yang, Z. Maximum likelihood phylogenetic estimation from DNA sequences with variable rates over sites: Approximate methods. *J. Mol. Evol.* **39**, 306–314 (1994).
5. Yang, Z. A space-time process model for the evolution of DNA sequences. *Genetics* **139**, 993–1005 (1995).
6. Soubrier, J. *et al.* The influence of rate heterogeneity among sites on the time dependence of molecular rates. *Mol. Biol. Evol.* **29**, 3345–3358 (2012).
