# Supplementary material for: Amalgamated cross-species transcriptomes reveal organ-specific propensity in gene expression evolution
Source: Nat Commun. 2020 Sep 8;11:4459. doi: 10.1038/s41467-020-18090-8 (PMC7479108; doi:10.1038/s41467-020-18090-8)
Supplement: Supplementary file 4 — Description of Additional Supplementary Files [file 41467_2020_18090_MOESM4_ESM.pdf]

Description of Additional Supplementary Files

Supplementary Data 1.
Curated metadata and characteristics of RNA-seq experiments.
Supplementary Data 2.
The numbers of RNA-seq experiments included in the final dataset with SVA-log-TMM-FPKM metrics.
Supplementary Data 3.
RNA-seq samples excluded by transcriptome amalgamation.
Supplementary Data 4.
Orthogroup statistics.
Supplementary Data 5.
The numbers and statistical significance of of PEO shifts.
Supplementary Data 6.
Significantly enriched terms in KEGG pathway enrichmnt analysis of genes involved in PEO shifts (adjusted P value < 0.05).
