# Supplementary material for: Amalgamated cross-species transcriptomes reveal organ-specific propensity in gene expression evolution
Source: Nat Commun. 2020 Sep 8;11:4459. doi: 10.1038/s41467-020-18090-8 (PMC7479108; doi:10.1038/s41467-020-18090-8)
Supplement: Supplementary file 2 — Peer Review [file 41467_2020_18090_MOESM2_ESM.pdf]

Reviewers' comments:

Reviewer #1 (Remarks to the Author):

This manuscript presents an analysis of gene expression shifts, in large gene families including orthologues and paralogues across 21 species. The authors develop an approach to amalgamate RNA-seq data from public resources and they use it to extract comparable samples from 6 major organs. Using this data, they analyze expression shifts in gene trees using phylogenetic Ornstein-Uhlenbeck models. They interpret their results in terms of "pre-adaptation" events, focusing on the connection between the ancestral gene expression pattern and its newly-acquired pattern.

The methodology presented by this manuscript is complex, and I can only assess parts of it. My first major concern is related to the validity of the transcriptome amalgamation process. The authors propose to iteratively remove RNA-seq samples (and corresponding BioProjects) if they present weaker expression correlation within/organs than between-organs. It is not clear whether this is done within species or across all species; given the supplementary figure S4 presenting each species separately, I'm assuming the former. While it is indeed expected to have stronger correlations within organ than between organs (for these major organs, at least), this is not a sufficient criterion. Combining data from so many public datasets can introduce many biases, in terms of dissection decisions, downstream treatment, RNA degradation, etc. Additional controls are needed. For example, I would suggest analyzing the expression patterns of cell-type specific markers, derived for example from single cell experiments, to evaluate whether the samples are comparable. In the end, given that this complex procedure only allowed analyzing 6 organs, I'm not sure it brings much compared to analyzing large datasets produced by a single research group (see for example Cardoso-Moreira et al., Nature 2019).

My second concern with the methodology is the application of the Ornstein-Uhlenbeck models. The authors say that "there is no available software to handle within-species variation in phylogenetic OU shift detection", but they do cite the manuscript by Rohlfs and co-authors, "Modeling Gene Expression Evolution with an Extended Ornstein–Uhlenbeck Process Accounting for Within-Species Variation", Mol Biol Evol, 2014. These methods had been implemented in R; I'm not sure if they are as computationally efficient as the l1ou package, but it would be good to test if the conclusions remain the same, at least for the smaller gene trees. Furthermore, with any of the methods, the validity of the conclusions will likely depend on the accuracy of the input gene trees. The authors mention only briefly that they redid the analyses by focusing only on "regime shifts found in clades which have a high support in tree inference" (page 12), but this is not sufficient. For all of these species, gene families and gene trees were constructed by Ensembl. How do the gene trees and inference of speciation/duplication nodes presented in this manuscript compare with the ones in Ensembl? Are the conclusions of the gene expression shifts analyses the same using these phylogenies as an input?

I am furthermore concerned with the interpretation of the results. The authors interpret all their findings in adaptive terms. There is however no evidence that the expression shifts are adaptive. In particular for duplicate genes, many of the observed expression changes (and omega shifts) may be signs of relaxation of purifying selection. The authors interpret the presence of preferred expression shift directions from pairs of organs (kidne/liver or brain/ovary/testis) as evidence for preadaptation. No other hypotheses are presented or discussed. There is no discussion of the cell type composition of the organs, of their developmental origins or of their shared physiological functions. The results are not compared with previous analyses of expression evolution after gene duplication - see for example the manuscript by Guschanski and co-authors, Genome Research, 2017.

The manuscript is very technical, and the results are not sufficiently discussed from a biological point of view. Paradoxically, the presentation of the results is also not quantitative: the authors should give actual quantitative assessments of their data, rather than using vague phrasing such as "often had more complementarity expression" or "nearly all ..." etc. Overall, I believe that this manuscript could

be of value (though perhaps would be better presented in a more specialized journal, such as Mol Biol Evol), if the biological interpretations and discussions of the results were extended and more carefully presented.

Reviewer #2 (Remarks to the Author):

Organ specific propensity drives patterns of gene expression evolution from Fukushima et al describe an exhaustive study of gene expression evolution in vertebrates. The authors processed an impressive amount of published RNA-seq data (1903 in total from 182 research projects) to study the gene expression evolution in 6 organs and 21 vertebrates' species. Fukushima et al implemented a quality control pipeline to exclude project specific biased and analysed the gene expression changes in gene trees. The gene expression shifts were classified by the authors in three different classes: S (if it was in a specification event in gene tree), D (a DNA duplication in gene tree) and R (a retro transposition related duplication in the gene tree).
This study has shown that (i) gene duplication lead to more shift in gene expression, (ii) gene expression change is correlated with protein sequence change, (iii) there is bias in gene expression shift form one organ to another. The results provided by this study contribute to a significant advance in the field

My major remark is that a major value of this study for the community is the processing and integration of an important dataset of gene expression assay from six tissues and 21 vertebrate species. However, the supplemental material provided with the manuscript has currently a limited reusability for the community. Additional information needs to be provided and especially the raw read count and the normalised one for each gene and each sample in each species. In addition, the supplemental material will need a mapping information between each gene for each species and the orthogroup it belongs. At the moment the information provided are very limited and it would be even difficult to recapitulate the results with the current supplementary material.

I have other major issues that will need to be addressed before it can be published.

1- The authors applied a careful methodology to minimise bias caused by the heterogeneity of data quality between projects. However, they did not talk about the difference in gene annotation between species that could potentially cause some bias at branch level. The authors need to explain how this issue could affect their result.

2- The authors find a relative abundant PEO shift related to testis. Can this be explained by the fact that testis is one of the tissues with the more expressed genes? If the analysis is done by normalising the number of PEO shift by the number of expressed genes in the donor tissues do the trends stay the same?

3- P8l75 : The authors need to explicit in the text that the R class are only intron-less genes if this is the case (or using the term retrocopy). If this is not only intron less, then they should explain more precisely their methodology to distinguish the D and R classes. I find this confusing the part of the sentences 'depending on intron losses'

We thank the reviewers for their thorough and thoughtful reviews. All criticisms are addressed in point-by-point fashion below. We believe this has resulted in an improved manuscript that is appropriate for a *Nature Communications* audience.

## Reviewer #1 (Remarks to the Author):

**This manuscript presents an analysis of gene expression shifts, in large gene families including orthologues and paralogues across 21 species. The authors develop an approach to amalgamate RNA-seq data from public resources and they use it to extract comparable samples from 6 major organs. Using this data, they analyze expression shifts in gene trees using phylogenetic Ornstein-Uhlenbeck models. They interpret their results in terms of "pre-adaptation" events, focusing on the connection between the ancestral gene expression pattern and its newly-acquired pattern. The methodology presented by this manuscript is complex, and I can only assess parts of it.**

We thank the reviewer for their helpful comments.

**My first major concern is related to the validity of the transcriptome amalgamation process. The authors propose to iteratively remove RNA-seq samples (and corresponding BioProjects) if they present weaker expression correlation within/organs than between-organs. It is not clear whether this is done within species or across all species; given the supplementary figure S4 presenting each species separately, I'm assuming the former.**

The iterative removals were performed within species. We revised Fig. S1 to better clarify the scope of this and other steps.

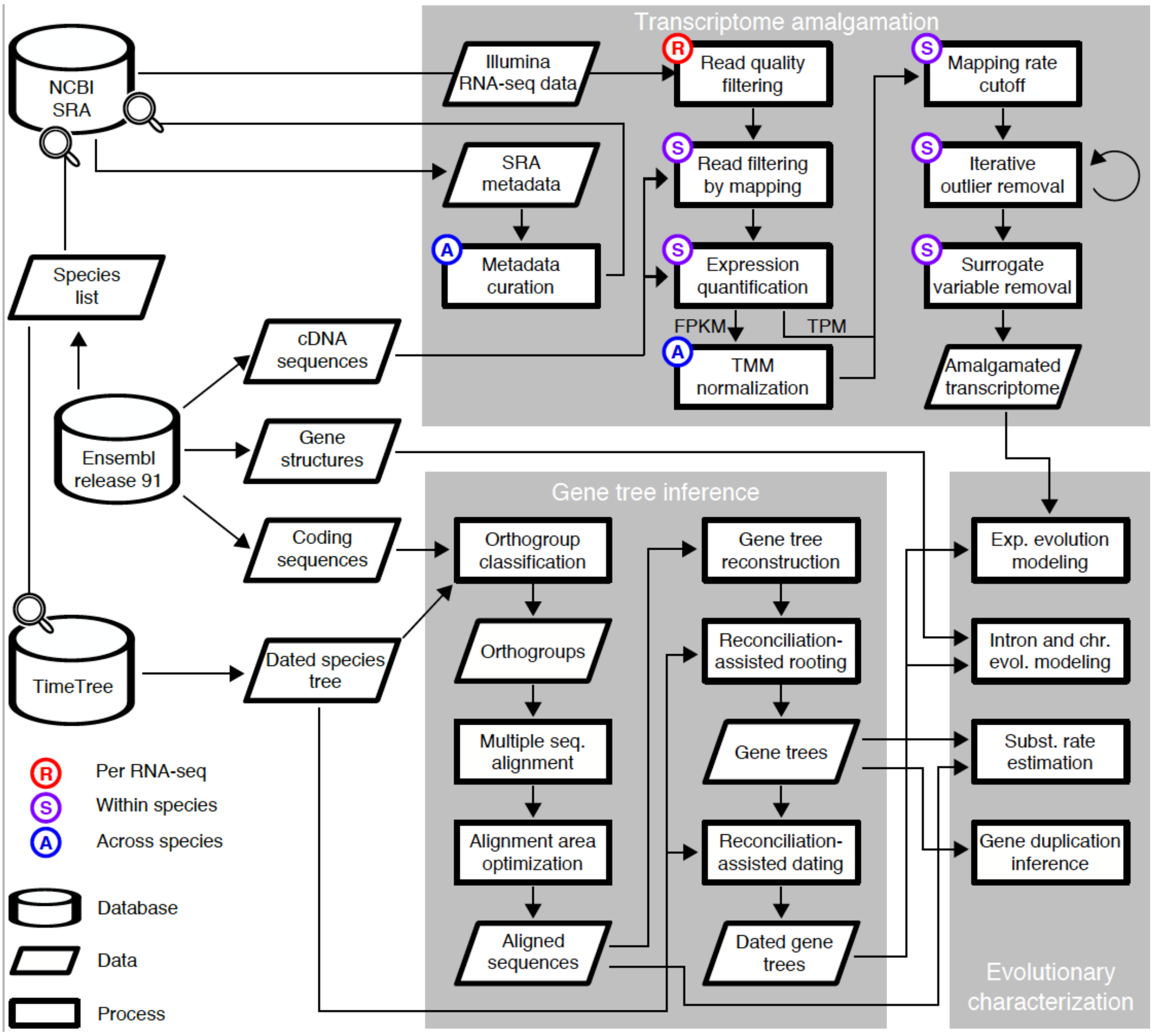

Fig. S1. A flow-chart of transcriptome amalgamation, gene tree inference, and evolutionary characterization in this study.

**While it is indeed expected to have stronger correlations within organ than between organs (for these major organs, at least), this is not a sufficient criterion. Combining data from so many public datasets can introduce many biases, in terms of dissection decisions, downstream treatment, RNA degradation, etc. Additional controls are needed. For example, I would suggest analyzing the expression patterns of cell-type specific markers, derived for example from single cell experiments, to evaluate whether the samples are comparable.**

We obtained curated cell-type-specific markers from PanglaoDB (https://panglaodb.se/markers.html), which exploits a number of scRNA-seq data in human and mouse, and checked its expression in our amalgamated transcriptomes. The results are presented in new Fig. S5. We found that the SVA correction improved the median values of relative marker gene expression; After correction, all RNA-seq data showed the corresponding marker expression values higher than those from the other organs, suggesting our amalgamated transcriptomes preserve organ-specific gene expression. This result was discussed in the main text as follows.

“To further evaluate the validity of amalgamated transcriptomes, we analyzed the expression of community-curated cell-type-specific marker genes associated with organs in PanglaoDB 35, which organizes a number of single-cell RNA-seq experiments in human and mouse. We compared the median values of log-transformed expression levels of >100 marker genes in each organ (Fig. S5). After SVA correction, all RNA-seq samples in both species showed the corresponding marker expression values higher than those from the other organs, suggesting our amalgamated transcriptomes preserve the organ-specific gene expression. In the cell-type-wise analysis, a few cases, such as juxtaglomerular cells in the kidney and hepatic stellate cells in the liver, could not resolve our organ-wise transcriptomes (Supplementary Data). Such low performance was seen in all samples rather than particular subsets, suggesting that the dissection decisions have negligible effects on cell type compositions in the organs.”

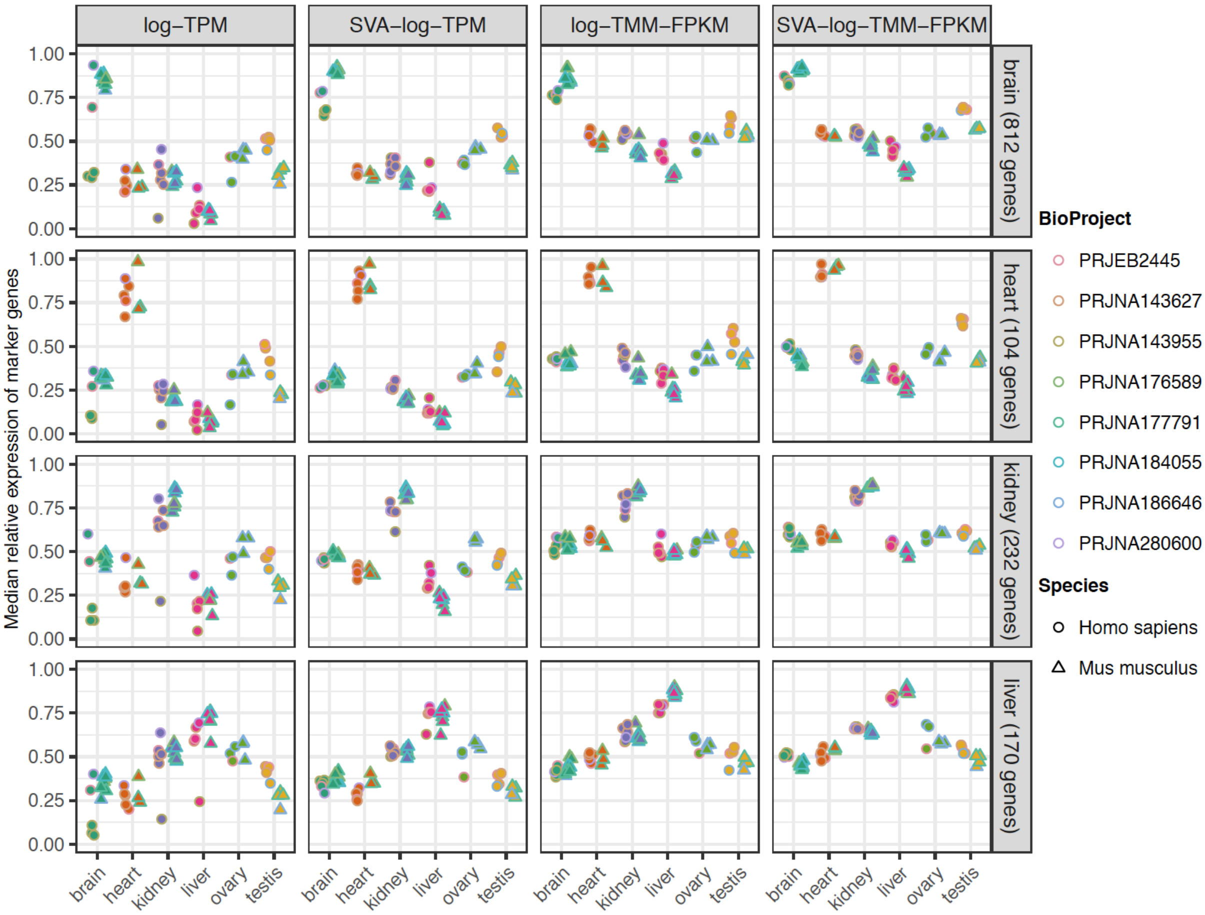


Fig. S5. Expression of organ-specific marker genes in human and mouse. Marker genes were retrieved from PanglaoDB 35, and its median expression values were obtained for each RNA-seq sample. A cell-type-wise analysis is provided in Supplementary Data. Cell types in ovary and testis were not included in PanglaoDB (access date: April 1, 2020).

**In the end, given that this complex procedure only allowed analyzing 6 organs, I'm not sure it brings much compared to analyzing large datasets produced by a single research group (see for example Cardoso-Moreira et al., Nature 2019).**

We thank the reviewer for pointing out this important viewpoint. Cardoso-Moreira et al. (2019) analyzed seven species for a developmental series of seven organs, whereas our analysis included 21 species. Greater species density (here, three-fold more species) allows

for better phylogenetic resolution of gene duplication events and identification of altered expression on lineages, especially after gene duplication events. A key advantage of our method is its extensibility. More species density for more organs will become available mostly from the contributions of multiple laboratories, not a single laboratory, and we need a means to integrate this multi-laboratory data sensibly. In this manuscript, we analyzed only six organs because it allowed us to include 21 species, but this is not a limitation of the method itself. The amount of available data in the NCBI SRA database is the limitation, and the procedure will be used for more organs as the species-diverse data becomes available. The database is rapidly growing by incorporating many new studies, including Cardoso-Moreira et al. (2019), so our method will open a venue for a larger scale of analysis without relying on a dataset from a single research group. An important question in any scientific research is whether results are replicable across study designs and research protocols. Our analysis gets at that question directly in a way that products of a single research group cannot. Furthermore, increasing species diversity is an essential aspect of improving comparative analysis, and this requires a community effort and the ability to integrate studies across research groups. We note the supporting comment of reviewer #2 that “a major value of this study for the community is the processing and integration of an important dataset of gene expression assay from six tissues and 21 vertebrate species.” This point is now included in the Discussion.

**My second concern with the methodology is the application of the Ornstein-Uhlenbeck models. The authors say that "there is no available software to handle within-species variation in phylogenetic OU shift detection", but they do cite the manuscript by Rohlfs and co-authors, "Modeling Gene Expression Evolution with an Extended Ornstein–Uhlenbeck Process Accounting for Within-Species Variation", Mol Biol Evol, 2014. These methods had been implemented in R; I'm not sure if they are as computationally efficient as the l1ou package, but it would be good to test if the conclusions remain the same, at least for the smaller gene trees.**

Rohlfs et al. (2014) describes phylogenetic multi-optima Ornstein-Uhlenbeck models. Their method requires *a priori* branch specification to detect lineage-specific regime shifts, unlike l1ou, which integrates a phylogenetic lasso combined with an information criterion to discover a set of regime shifts among the number of branch combinations which grows exponentially with the size of trees. Therefore, without modifications, Rohlfs et al. (2014)’s method *is not applicable for our analysis*, in which the place and number of regime shifts cannot be informed before analysis. PhylogeneticEM and SURFACE can handle an exploratory analysis like l1ou, but neither of them takes into account within-species variation. As pointed out by the reviewer, Rohlfs et al. (2014)’s method takes advantage of within-species variation. They pointed out the importance of within-species variation to accurately detect purifying selection over neutral drift. This is not relevant to our manuscript because we did not examine whether genes evolved under stabilizing selection or neutral drift, a problem well-studied in previous work (Chen et al., 2019, Genome Res 29: 53-63, and references therein). Rohlfs et al. (2014) also mentioned that the expression shift detection showed similar high power between species mean and species variance models, suggesting that our species mean model is expected to perform as well as the species variance model in terms of shift detection. In the previous version of manuscript, our phrasing was not specific enough, so we edited the sentence the reviewer pointed out as follows.

"Because there is no available software to handle within-species variation in phylogenetic OU shift detection without predefined hypotheses on the number and place of regime shifts, we used mean expression level as the input. It is shown by simulation that the species mean and species variance models show comparable power for the regime shift detection (Rohlfs et al., 2014), suggesting that our species mean model is expected to perform as well as the species variance model."

**Furthermore, with any of the methods, the validity of the conclusions will likely depend on the accuracy of the input gene trees. The authors mention only briefly that they redid the analyses by focusing only on "regime shifts found in clades which have a high support in tree inference" (page 12), but this is not sufficient. For all of these species, gene families and gene trees were constructed by Ensembl. How do the gene trees and inference of speciation/duplication nodes presented in this manuscript compare with the ones in Ensembl? Are the conclusions of the gene expression shifts analyses the same using these phylogenies as an input?**

The Ensembl Compara's algorithm for speciation/duplication inference (https://m.ensembl.org/info/genome/compara/homology_types.html) is a species overlap method, which we used in this paper. We first calculated the "duplication confidence score", and nodes were considered duplication if the score is greater than 0 as in the Ensembl dataset. Therefore, the node classification should be quite similar between Ensembl and our datasets if tree topology is the same. Because gene family classification in Ensembl Compara is different from our OrthoFinder-based classification, the use of the Ensembl trees will not yield results directly comparable to our original analysis. A key feature of the tree reconstruction in Ensembl Compara is the use of phylogeny reconciliation, which takes into account duplication-loss parsimony (https://www.ensembl.org/info/genome/compara/homology_method.html). To address the reviewer's question and mimic this procedure without changing the orthogroup classification, we obtained reconciled gene trees using GeneRax and reproduced downstream analyses. Overall, the results are similar (new Fig. S12). The difference in regime shift numbers did not correlate to the topological differences between the original and reconciled trees, suggesting that our results are robust against errors in tree topology. The new data are presented in the new Fig. S12, and discussed in the main text as follows.

"The effect of gene trees was further examined by replicating the analysis with alternative tree topologies inferred by species tree reconciliation, which takes into account duplication–loss rates 50. This reconciliation step is expected to correct erroneous tree topology, while possibly introducing another bias derived from over-correction of biological signals such as incomplete lineage sorting. With the reconciled trees, the OU modeling with SVA-log-TMM-FPKM values resulted in equivalent numbers of expression shifts in S and D branches compared with those with non-reconciled trees (97% [23,231/23,985] and 104% [9,407/9,018], respectively) (Fig. S12A). In contrast, the phylogeny reconciliation substantially reduced the number of shifts in R branches (39% [481/1,238]). This could be explained by the correction of erroneous tree topology caused by the fast-evolving retro-copies (Fig. 4A), although the differences in shift numbers did not correlate with the topological differences measured by Robinson-Foulds distance 51 (Fig. S12B). Nevertheless, resulting PEO shift distributions were largely similar (Fig. S12C), with the reproduced accelerations in the brain–testis–ovary and kidney–liver modules (Fig. S12D)

and the asymmetric PEO shifts in R branches (Fig. S12E), suggesting the robustness of detected modules against gene tree topology.”

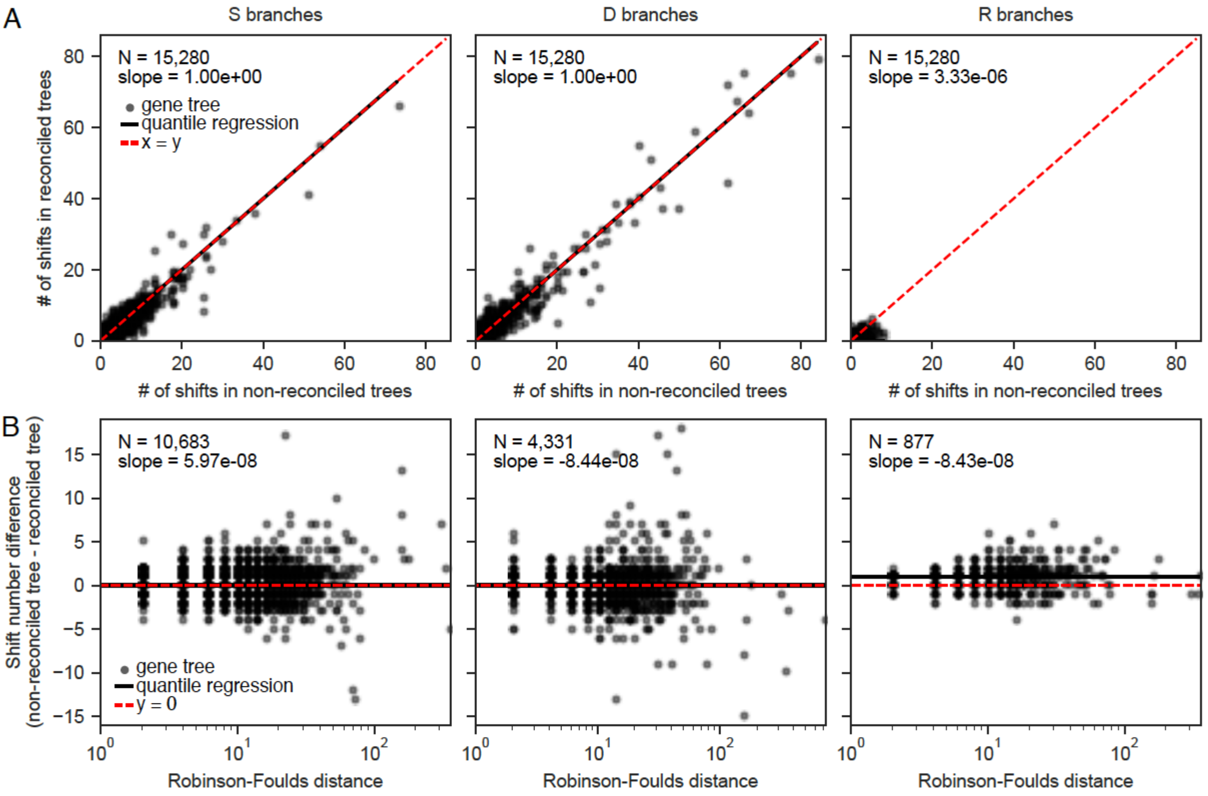

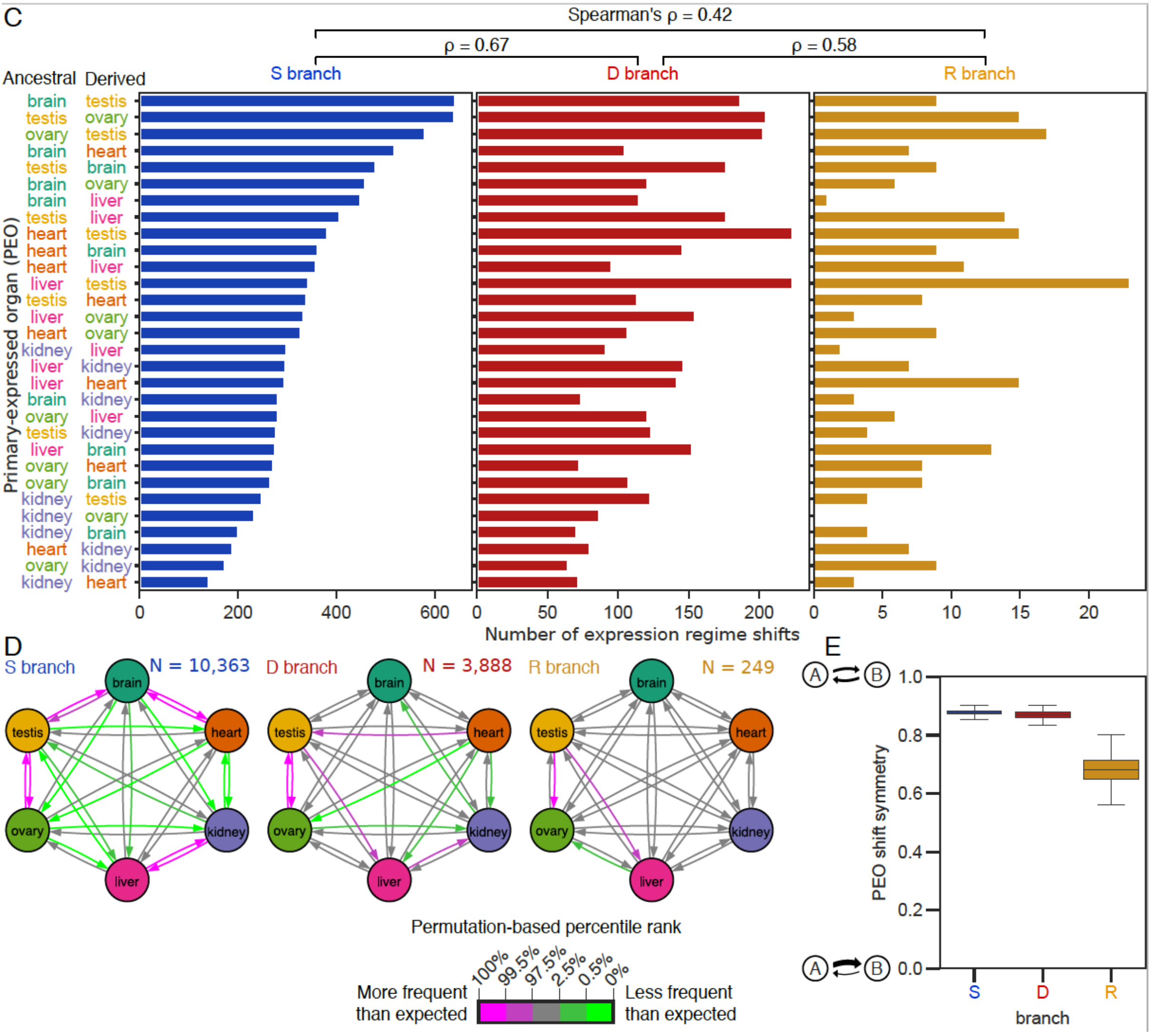


Fig. S12. Effects of phylogeny reconciliation. (A) The numbers of detected shifts in non-reconciled and reconciled maximum-likelihood gene trees. (B) The relationship between shift number difference and tree topology difference measured by Robinson-Foulds distance 51. Trees with no detected shifts were removed from the analysis. (C–E) Reproduction of PEO shift distributions using reconciled gene trees. C, D, and E correspond to Fig. 5A, Fig. 5B, and Fig. 5C, respectively.

**I am furthermore concerned with the interpretation of the results. The authors interpret all their findings in adaptive terms. There is however no evidence that the expression shifts are adaptive. In particular for duplicate genes, many of the observed expression changes (and omega shifts) may be signs of relaxation of purifying selection.**

The reviewer may have misunderstood our use of the term “pre-adaptive” to imply that the expression shifts themselves are due to adaptive forces. This is not the case, and we agree that it is a good idea to clarify the possible role (or lack of a role) for adaptation for both the expression shifts and amino acid acceleration. We have expanded our discussion of the issues around what can happen after gene duplication, including relaxation of purifying selection, neofunctionalization, subfunctionalization, and escape from adaptive conflict. Please see below.

**The authors interpret the presence of preferred expression shift directions from pairs of organs (kidne/liver or brain/ovary/testis) as evidence for preadaptation. No other hypotheses are presented or discussed.**

The hypothesis testing around this issue contrasts a null model of random movement of genes among organs (controlled for the number of genes with major expression in each organ) to the hypothesis of preadaptation, which is the idea that adaptation for dominant expression in one particular organ predisposes a gene to be amenable to dominant expression in another particular organ. In response to this and other comments, we have added further discussion on the ways that such preadaptation might happen, including common cell type, developmental origins, shared physiology, and shared biochemical environments.

"Our results suggest that the landscape of expression evolution is strongly shaped by mechanisms of gene birth. Expression shifts are more pronounced following gene duplication in agreement with the results of pairwise gene expression analyses [22,24,43], and shifts in patterns of primary-expressed organs strongly depend on the expression state in the ancestral organism. Thus, by analyzing such influences on a genome-wide scale for a moderately large number of species, the question whether long-term expression in one organ predisposes genes to be subsequently utilized in other organs has been answered in the affirmative. There are preadaptive propensities in the evolution of vertebrate gene expression, and the propensity varies with the presence and type of gene duplication. Furthermore, the approach developed in this study, using complex gene family phylogenies including gene duplications and losses that do not assume perfect match to the species phylogeny, and incorporating a curation pipeline to amalgamate large amounts of transcriptome data from many studies, was essential to obtain the necessary species density and phylogenetic resolution to answer this question. The extensibility of this method will allow for more species and more organs to be incorporated as further studies come into the literature from diverse laboratories.

The mechanisms responsible for the preadaptive propensities that influence expression shifts among organs are, however, unknown. A key question in understanding these shifts may be the role of adaptation in the shift, and in subsequent evolution. We have been careful so far to simply describe the shifts, but adaptive possibilities include subfunctionalization, escape from adaptive conflict (EAC), and neofunctionalization [54–56]. The increased number of shifts following duplication suggests that drift alone is not the explanation, but subfunctionalization easily could be. Subfunctionalization is the idea that, if a gene has multiple functions prior to duplication, they may be segregated among the duplicates following gene duplication. Thus, the expression shifts may be simply a shift in focus of a duplicated copy on a subset of the necessary expression profile needed at the organismal level. In this scenario, any accompanying acceleration of amino acid substitution would be caused by a loss of constraint and reduced purifying selection in one expression environment or the other.

EAC involves more adaptation by adding the simple idea that prior to duplication, the multiple functions and expression regimes were at least partially in conflict. Such conflicts could clearly occur at the amino acid level, but could also occur at the expression level. For example, if expression levels were focused on a most-important tissue or most-sensitive tissue prior to duplication, but after duplication could be more tailored to what is better for the new expression regime. Finally, neofunctionalization would occur at the sequence or expression level, if the loss of selection on a duplicate allowed mutations that were

previously harmful to the old function, but now are not, and are able to carry out some novel functional aspect that was previously prohibited. Neofunctionalization is perhaps the most interesting and extraordinary possible cause for the expression regime shifts we see, but it requires strong evidence and it is not a necessary explanation for what we observe.

In this context, the patterns of expression regime shifts we observed may be explained at different levels of biological organization, from the tissues and cells that make up organs, to subcellular compartments, chromatin structure, promoter usage, and protein biochemistry. Part of the propensity shifts we observed can be explained by the "out of the testis" hypothesis, which posits that accelerated gains of testis expression are based on the permissive chromatin state, abundant transcriptional machinery, relatively simple promoters required for the expression in spermatogenic cells and following gains of new expression patterns [4,57]. This theory fits to the accelerated testis-related PEO shifts, and could fit with any of the adaptive scenarios discussed above, but the other detected patterns (e.g., kidney–liver module) require other explanations.

One potential mechanism of preferences in expression regime shifts is a cell-type or sub-cellular component mechanism. In such a mechanism, if two organs tend to share cell types or usage of sub-cellular components, they may be prone to expropriate genes between the two organs. It is known that gene expression levels in the kidney and liver tend to change jointly, possibly reflecting their similar physiology including detoxifications and waste excretion [19]. Such functional similarity may also explain the presence of the kidney–liver module of gene exchange."

**There is no discussion of the cell type composition of the organs, of their developmental origins or of their shared physiological functions. The results are not compared with previous analyses of expression evolution after gene duplication - see for example the manuscript by Guschanski and co-authors, Genome Research, 2017.**

In response to this comment, we have added a section in the discussion about this topic. Please see above.

**The manuscript is very technical, and the results are not sufficiently discussed from a biological point of view. Paradoxically, the presentation of the results is also not quantitative: the authors should give actual quantitative assessments of their data, rather than using vague phrasing such as "often had more complementarity expression" or "nearly all ..." etc.**

We include a great deal of supplementary data that provides quantification of our results in addition to what is in the main paper. In addition, we have gone through the descriptive text and pulled quantitative data into it, or reiterated where the quantitative data is to be found in cases where there is too much of it or it would destroy the flow of the explanation.

**Overall, I believe that this manuscript could be of value (though perhaps would be better presented in a more specialized journal, such as Mol Biol Evol), if the biological interpretations and discussions of the results were extended and more carefully presented.**

As mentioned above, we have added considerable discussion of the details of selective shifts following gene duplication, as well as discussion on the mechanistic possibilities for generating preferred flux of expression among specific tissue types. We believe that the nature of our findings impacting our understanding of evolution of expression patterns, and

providing a validated, quality-controlled, and improved methodology for integrating evolution of expression data across datasets, which is key for the future of comparative analysis in this area, make our paper of considerable interest to a broad audience such as that of Nature Communications. To quote reviewer #2, "*The results provided by this study contribute to a significant advance in the field.*"

## Reviewer #2 (Remarks to the Author):

**Organ specific propensity drives patterns of gene expression evolution from Fukushima et al describe an exhaustive study of gene expression evolution in vertebrates. The authors processed an impressive amount of published RNA-seq data (1903 in total from 182 research projects) to study the gene expression evolution in 6 organs and 21 vertebrates' species. Fukushima et al implemented a quality control pipeline to exclude project specific biased and analysed the gene expression changes in gene trees. The gene expression shifts were classified by the authors in three different classes: S (if it was in a specification event in gene tree), D (a DNA duplication in gene tree) and R (a retro transposition related duplication in the gene tree). This study has shown that (i) gene duplication lead to more shift in gene expression, (ii) gene expression change is correlated with protein sequence change, (iii) there is bias in gene expression shift form one organ to another. The results provided by this study contribute to a significant advance in the field**

We thank the reviewer for their kind comments and appreciation of the magnitude of effort required for this work.

**My major remark is that a major value of this study for the community is the processing and integration of an important dataset of gene expression assay from six tissues and 21 vertebrate species.**

We agree that this is a major value of this study.

**However, the supplemental material provided with the manuscript has currently a limited reusability for the community. Additional information needs to be provided and especially the raw read count and the normalised one for each gene and each sample in each species. In addition, the supplemental material will need a mapping information between each gene for each species and the orthogroup it belongs. At the moment the information provided are very limited and it would be even difficult to recapitulate the results with the current supplementary material.**

As requested, we have deposited all data required to reproduce this study in Mendeley Data where the preview link is already available (https://data.mendeley.com/datasets/3vcstwdbrn/draft?a=ff803d44-48cd-482a-a4eb-fe78703f211f). Available files include orthogroup mappings, amalgamated transcriptomes, gene trees, OU models, and visualization of all trees similar to Fig. 2D. In response to this comment, we have added further details in the "Code and data availability".

"**Code and data availability.** Scripts, parameter values, gene expression values including SVA-log-TMM-FPKM and SVA-log-TPM, and other data used in this study are available as Supplementary Data (Mendeley Data ID available on publication, preview link:

https://data.mendeley.com/datasets/3vcstwdbrn/draft?a=ff803d44-48cd-482a-a4eb-fe78703f211f).”

**I have other major issues that will need to be addressed before it can be published. 1- The authors applied a careful methodology to minimise bias caused by the heterogeneity of data quality between projects. However, they did not talk about the difference in gene annotation between species that could potentially cause some bias at branch level. The authors need to explain how this issue could affect their result.**

We added the analysis of the gene annotation completeness using BUSCO 4.0.5, which resulted in a slightly lower scores in early-diverging taxa (new Fig. S8). Because this pattern matched to the rate of expression shifts in R branches, we discussed the gene completeness as a confounding factor as follows.

“Among-species heterogeneity in gene prediction quality may also be attributed to this pattern because early-diverging species tended to show higher percentages of missing single-copy orthologs than those in mammalian species (Fig. S8; but see Danio rerio, Oreochromis niloticus, and Oryctolagus cuniculus as counterexamples).”

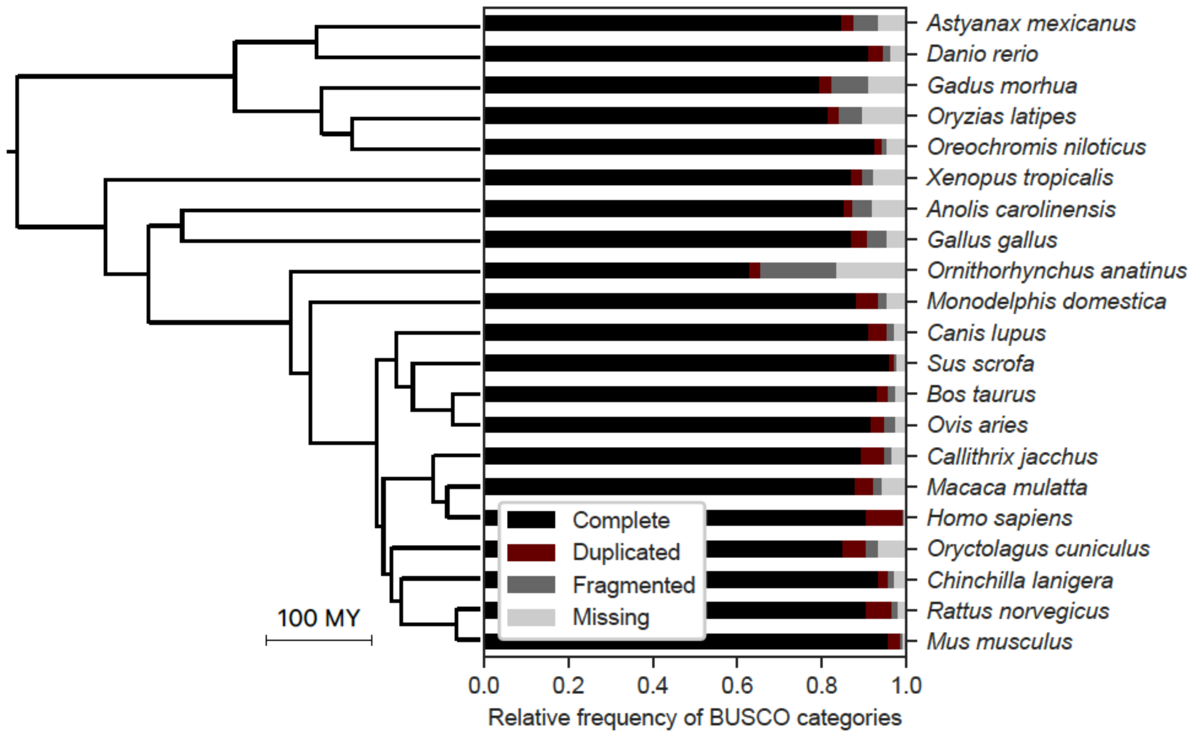


Fig. S8. Evaluation of gene set completeness. BUSCO analysis was performed for gene sets from 21 species using 3,407 single-copy orthologs in the dataset “vertebrata_odb10”. The species tree is shown to visualize lineage-specific trends.

**2- The authors find a relative abundant PEO shift related to testis. Can this be explained by the fact that testis is one of the tissues with the more expressed genes? If the analysis is done by normalising the number of PEO shift by the number of expressed genes in the donor tissues do the trends stay the same?**

To test the effect of the number of expressed genes, we took into account its heterogeneity and performed an analysis similar to Fig. 5A. The result is presented in new Fig. S10. The

bar chart is sorted by the numbers of baseline shifts (on S branches). For consistency, Fig. 5A is now sorted by shifts on S branches. The corrected data showed a similar pattern with abundant testis-related PEO shifts. We discussed this point as follows.

“D branches were moderately similar to both S and R branches (Spearman’s ρ ~ 0.6), but S and R branches were dissimilar (ρ = 0.28). This pattern, including the abundant shifts related to testis, was robust against the correction by the organ-wise numbers of expressed genes (Fig. S10). This result suggests a role for gene duplication, including by retrotranspositions, in remodeling the among-organ flow of expressed genes.”

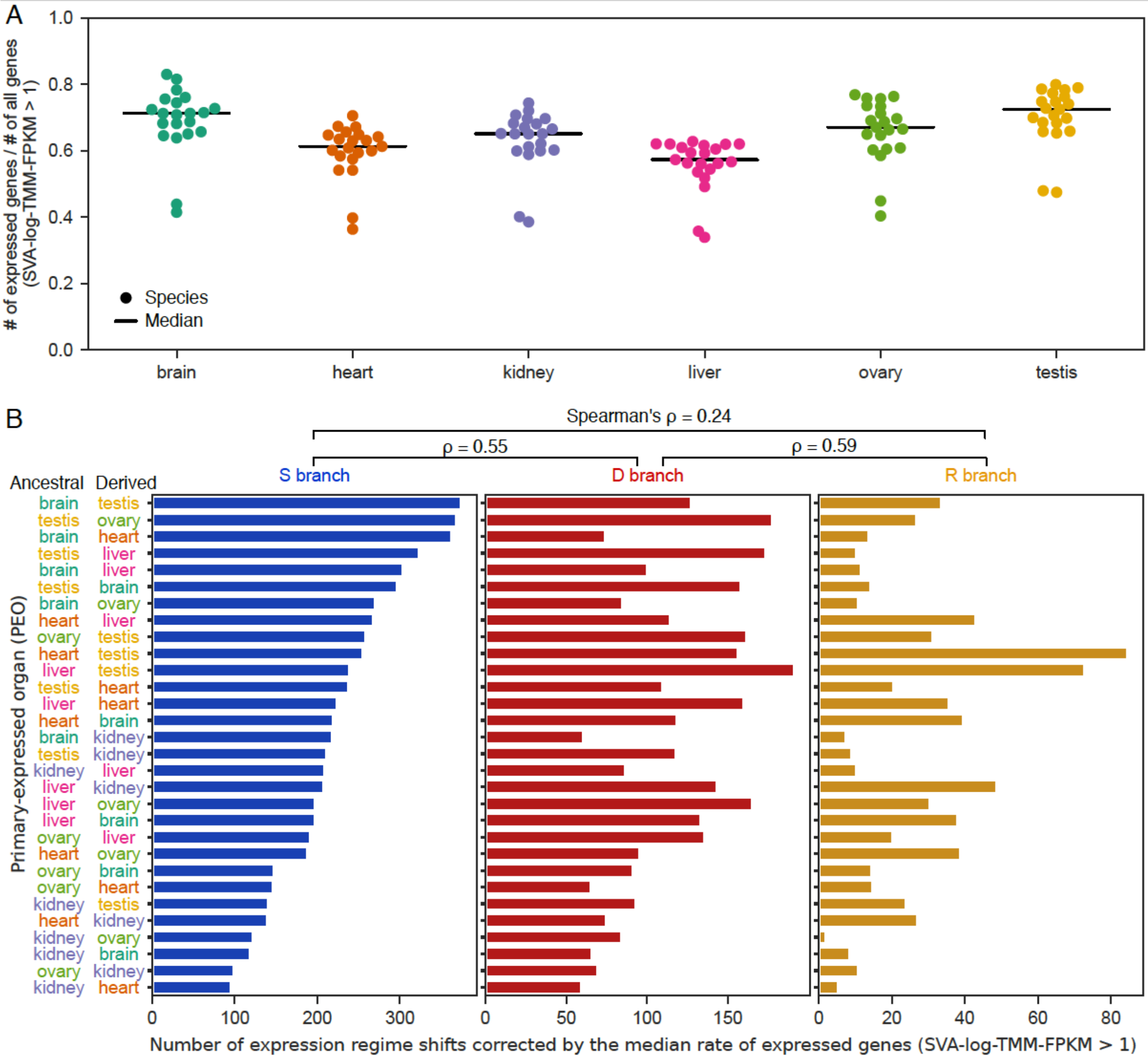


Fig. S10. The number of expressed genes does not explain the organ-wise abundance of PEO shifts. (A) The organ-wise numbers of expressed genes in the 21 species. In this analysis, expressed genes are defined as genes with >1 SVA-log-TMM-FPKM. (B) The PEO shift distributions corrected by the median numbers of expressed genes. The data in Fig. 5A were corrected as follows: …(equation)... The former corresponds to the numbers shown in Fig. 5A. M_ancestral and M_derived are median numbers of expressed genes in ancestral and derived PEOs, respectively. M¯indicates the across-organ average of expressed gene numbers.

**3- P8l75 : The authors need to explicit in the text that the R class are only intron-less genes if this is the case (or using the term retrocopy). If this is not only intron less, then they should explain more precisely their methodology to distinguish the D and R classes. I find this confusing the part of the sentences 'depending on intron losses'**

We annotated R branches if introns are completely lost (e.g., 5 introns -> 0 introns). Partial loss (e.g., 5 introns -> 3 introns) did not interfere with the results because we modelled the intron evolution only with the intron-containing and intron-less states as explained in Materials and Methods. We changed the phrasing of the sentence in the main text to improve communication of this point as follows.

"Gene tree nodes associated with preceding duplication events were categorized as DNA-based duplication or retrotransposition (D or R nodes, respectively) depending on complete intron losses (Fig. 2B). "

Reviewer #1 (Remarks to the Author):

The authors have addressed most of my initial comments appropriately. I do not have any more technical comments. However, I am still troubled by the use of the "pre-adaptation" term, which does indeed strongly suggest that the expression shifts analyzed by the authors are adaptive. This is addressed in the discussion, but it should come up earlier, already in the abstract, as soon as the "preadaptive" term is used.

Reviewer #3 (Remarks to the Author):

Fukushima and Pollock present a comprehensive analysis of how gene expression evolves following gene duplication events (across 21 vertebrate species). They make the following claims:

- Duplicated genes, especially from RNA-based duplications, are more likely to shift in expression
- Changes in proteins tend to accompany changes in expression
- Expression shifts between certain "modules" of organs (e.g. kidney/liver) are more likely than others

This analysis is interesting and comprehensive in its scope and would be of interest to evolutionary biologists, particularly those interested in molecular/gene expression evolution.

Before publication, I have two major comments and a few minor comments:

The first comment that I feel must absolutely be addressed prior to publication is that there is a lack of statistical testing for claims through out the manuscript. Here I have listed the claims that not have been thoroughly tested with suggestions of appropriate tests in parentheses:
- difference frequency of regime shifts in S/D/R branches after accounting for different number of branches (Pearson's chi-sq test)
- all claims associated with figures 3D, 3E, 3F (KS test for difference in distribution)
- all claims associated with figure 4B (could permute data many times to calculate empirical p-value of slopes)
- all claims associated with figure 5B (Pearson's chi-sq test), 5C (KS test)

My second comment is regarding the robustness of the author's results. Making gene trees, accurately quantifying gene expression, especially of paralogs, and accurately fitting an OU model are all difficult analyses that are highly subjected to technical confounders. For the last analysis looking at organ-shift patterns in expression regime changes, the authors re-do the analysis on regime shifts found in clades which have a high support in tree inference but I would like to see if all the analyses hold up when re-done on this set of high-confidence regime shifts. I am particularly wondering if the analyses related to protein changes/expression changes also hold up when using this more confident set of regime shifts.

Minor comments:

1) I am confused by the use of both TPM and FPKM throughout this paper. TPM is a more mathematically correct unit of RNA abundance and is preferred (https://pubmed.ncbi.nlm.nih.gov/22872506/). Furthermore, presenting both sets of data is confusing and renders the paper difficult to read. I would advocate to presenting only TPM results throughout the paper. Furthermore, the authors say that TMM normalization is not suitable for TPM but I'm unclear as to why this is? In either case, I don't know if using TMM normalized TPM would change the results in any major way, but they may want to check the normalization factors when using TPM to confirm this is true.

2) Figure 1F seems unnecessary and a statistical truism - variance around the mean will necessarily decrease as sample size increases.

3) Line 377 - "Although the adjusted P value was not statistically significant, it is noteworthy that the top-ranked term for the brain–testis connection was “Endocrine and other factor-regulated calcium reabsorption” annotated to four genes including GNAQ, which has been implicated to tumor formation in neuronal tissues. " - what is the adjusted p-value here? If very high (>0.3), I do not think this result should be reported.

We thank the reviewers for their thorough and thoughtful reviews. All criticisms are addressed in point-by-point fashion below. We believe this has resulted in an improved manuscript that is appropriate for a *Nature Communications* audience.

## Reviewer #1

**The authors have addressed most of my initial comments appropriately. I do not have any more technical comments. However, I am still troubled by the use of the "pre-adaptation" term, which does indeed strongly suggest that the expression shifts analyzed by the authors are adaptive. This is addressed in the discussion, but it should come up earlier, already in the abstract, as soon as the "preadaptive" term is used.**

**Response:** In response to this comment, we added a phrase in the abstract to draw the reader's attention to this point.

**Change:** Thus, if expression shifted, ancestral expression in some organs induces a strong propensity for expression in particular organs in descendants. Regardless of whether the shifts are adaptive or not, this supports a major role for what might be termed "preadaptive" pathways of gene expression evolution.

## Reviewer #3

**Fukushima and Pollock present a comprehensive analysis of how gene expression evolves following gene duplication events (across 21 vertebrate species). They make the following claims:**

**- Duplicated genes, especially from RNA-based duplications, are more likely to shift in expression**
**- Changes in proteins tend to accompany changes in expression**
**- Expression shifts between certain "modules" of organs (e.g. kidney/liver) are more likely than others**

**This analysis is interesting and comprehensive in its scope and would be of interest to evolutionary biologists, particularly those interested in molecular/gene expression evolution.**

**Before publication, I have two major comments and a few minor comments:**

**Response:** We thank the reviewer for their kind comments.

**The first comment that I feel must absolutely be addressed prior to publication is that there is a lack of statistical testing for claims throughout the manuscript.**

**Response:** In large-scale genomics studies such as this, it is common to not always use statistical testing in cases where differences are obvious and the p-values absurdly low. We have tried to focus instead on the magnitude and biological relevance of the effects, which we think is appropriate. Nevertheless, we performed all tests as requested (with the exception of figure 5B, where we had already presented permutation-based percentiles, or p-value categories) and added them to the manuscript, as described in detail below.

**Here I have listed the claims that not have been thoroughly tested with suggestions of appropriate tests in parentheses:**
**- difference frequency of regime shifts in S/D/R branches after accounting for different number of branches (Pearson's chi-sq test)**

**Response:** In response to this comment, we performed a chi-square test and presented the result in the main text.

**Change:** Across gene trees, per-branch frequencies of expression regime shifts were significantly different among S, D, and R branches (P ≈ 0; $\chi^2$ = 2.11×$10^4$; $\chi^2$ test).

**- all claims associated with figures 3D, 3E, 3F (KS test for difference in distribution)**

**Response:** In response to this comment, we performed KS tests for pairwise comparisons among S, D, and R branches in Fig. 3D-F, and presented the results in the figure.

**Change:**

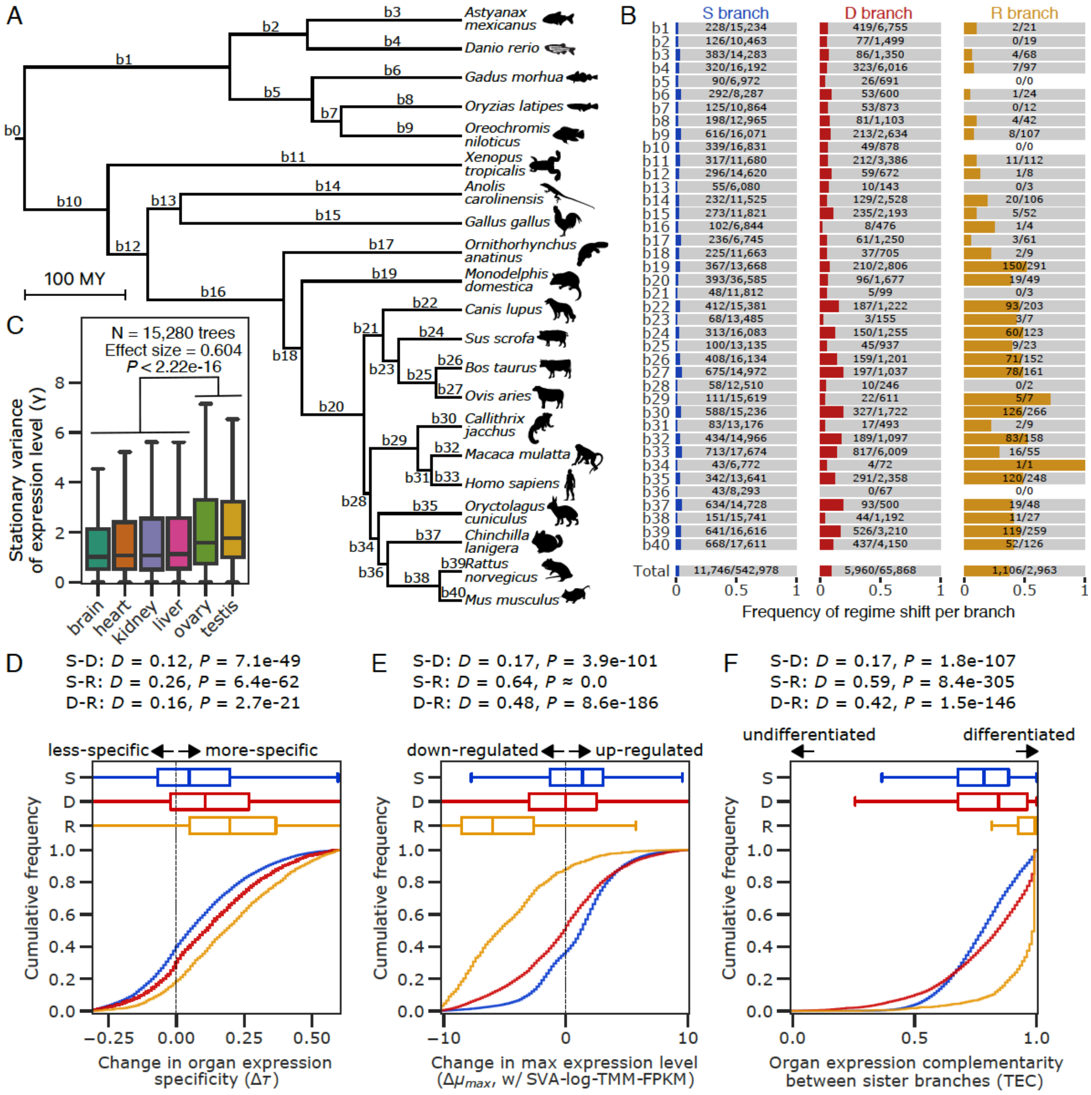


**Figure 3. Characteristics of expression shifts in 15,280 gene trees.** (**A**) The species tree showing analyzed genomes and their divergence time. (**B**) Mapping of 18,812 expression

shifts in the species tree. The number and proportion of expression regime shifts in S, D, and R branches are shown. Corresponding branches in the species tree are indicated in **A**. (**C**) Organ-specific stationary variances (γ) of expression level evolution in vertebrates. The distribution of γ between reproductive and non-reproductive organs were compared by a Brunner-Munzel test [95]. (**D–F**) Cumulative frequency of change in organ expression specificity (**D**), change in maximum expression level (**E**), and expression complementarity between sister lineages (**F**) among detected expression shifts. Number of analyzed regime shifts are shown in **B**. The *D* statistics and *P* values of pairwise branch category comparisons were calculated with two-sided Kolmogorov–Smirnov tests.

**- all claims associated with figure 4B (could permute data many times to calculate empirical p-value of slopes)**

**Response:** In response to this comment, in Fig. 4B, we added P values indicating whether the slopes were significantly different from zero. We applied the same change to Fig. S9.

**Change:**

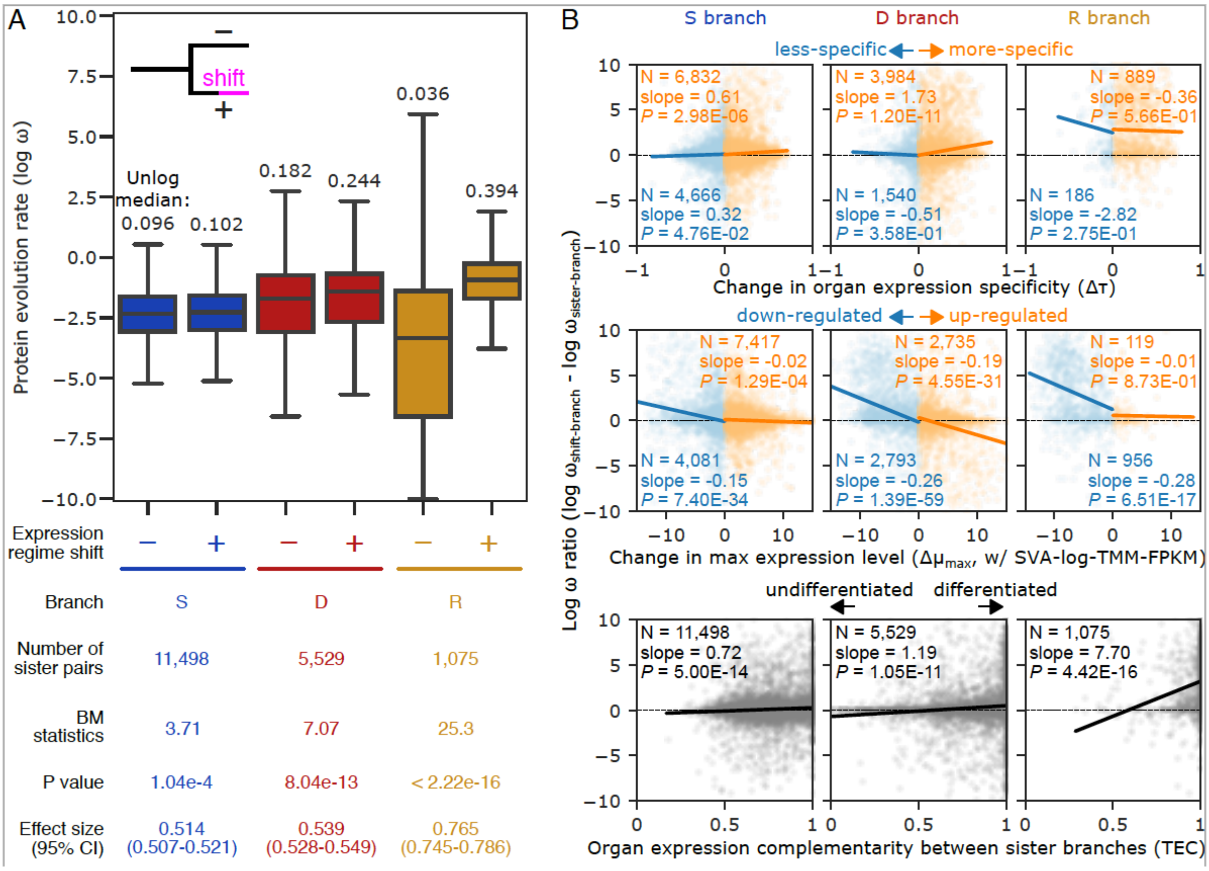


**Figure 4. Context-dependent changes of protein evolution rate coupled with expression regime shifts.** (**A**) Distribution of ω values. A plus (+) indicates branches with expression shifts, whereas minus (−) branches are sisters to the 'plus' branches. Statistical differences between pairs of distributions were tested using a two-sided Brunner–Munzel test [95]. Non-log-transformed median values are shown above the boxplots. For visualization purposes, extreme values exceeding ±10 were clipped. (**B**) Relationships between protein evolution rate

and change in expression properties. Points correspond to expression regime shifts. Dashed lines indicate no between-branch difference in ω. Solid lines show a linear regression. Its slope and number of regime shifts are provided in the plot. Regime shifts with negative and positive changes were separately analyzed for organ expression specificity (upper) and maximum expression level (middle). *P* values indicate whether the slopes were significantly different from zero (two-sided *t* tests).

## - all claims associated with figure 5B (Pearson's chi-sq test), 5C (KS test)

**Response:** Because permutation-based percentile ranks are already available, we did not change Fig. 5B, but raw data and test statistics are now included as new Table S5. For Fig. 5C, we performed KS tests and presented the results in the figure.

**Change:** Controlling the total number of shifts from and to each PEO, some PEO shifts are significantly different from the random expectation (Fig. 5B; Table S5).

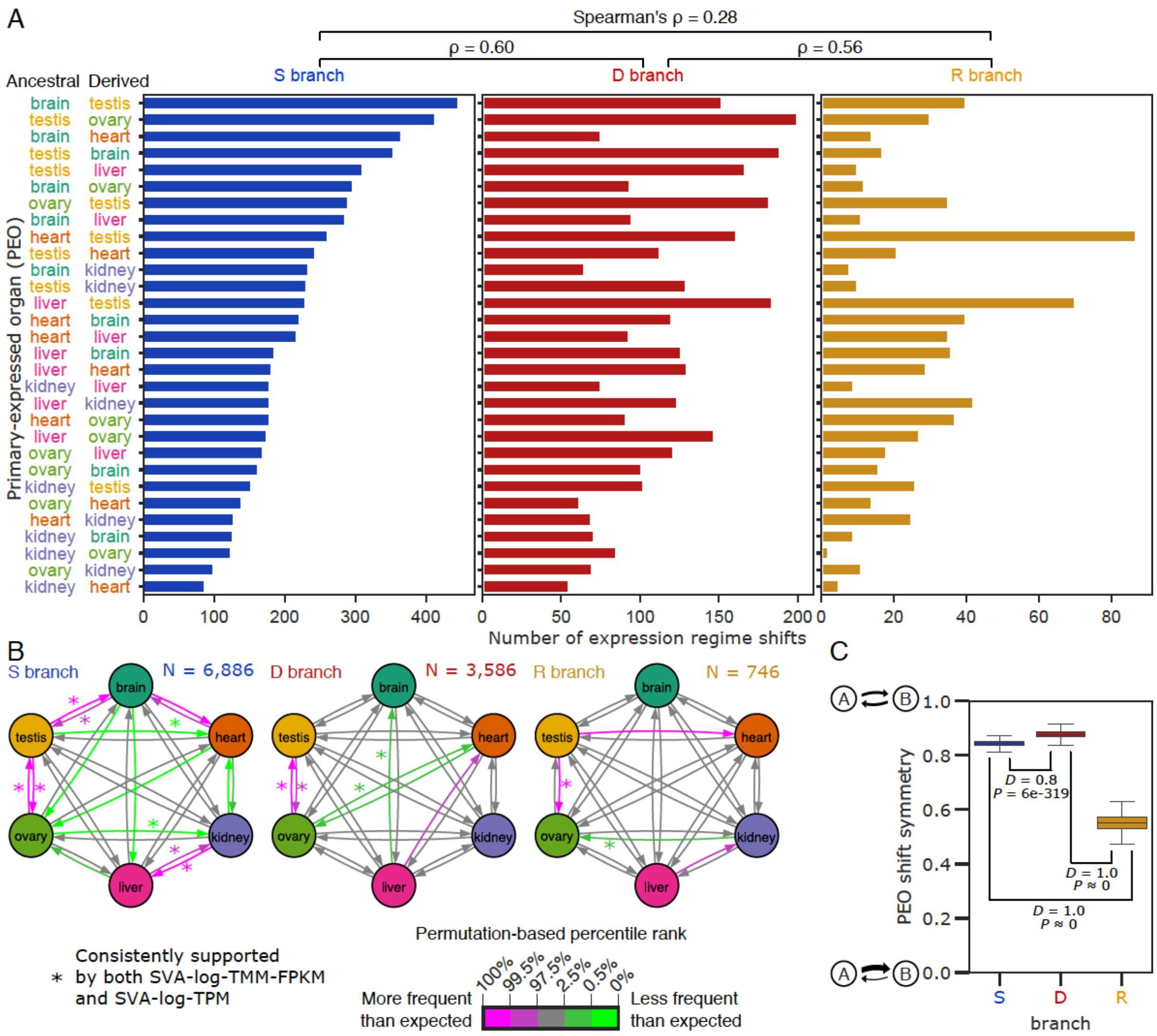


**Figure 5. Evolutionary dynamics of gene expression.** (**A**) Shift distributions of primary-expressed organs (PEOs). Y-axis was sorted by abundance in S branches. Spearman's correlation coefficients among S, D, and R branches are shown above the plots. (**B**) Preadaptation networks in organ expression. Arrows represent transitions from ancestral

PEOs to derived PEOs, and its color shows statistical significance based on 10,000 permutations. The results were obtained with SVA-log-TMM-FPKM, and an asterisk (*) indicates the statistical significance supported also by SVA-log-TMM-based analysis (Fig. S11). (**C**) The global polarity of PEO shifts. The global polarity is defined by the scaled sum of differences between two opposite PEO shifts. Boxplots show the distribution estimated by 1,000 bootstrap resampling. The *D* statistics and *P* values of pairwise branch category comparisons were calculated with two-sided Kolmogorov–Smirnov tests.

**My second comment is regarding the robustness of the author's results. Making gene trees, accurately quantifying gene expression, especially of paralogs, and accurately fitting an OU model are all difficult analyses that are highly subjected to technical confounders. For the last analysis looking at organ-shift patterns in expression regime changes, the authors re-do the analysis on regime shifts found in clades which have a high support in tree inference but I would like to see if all the analyses hold up when re-done on this set of high-confidence regime shifts. I am particularly wondering if the analyses related to protein changes/expression changes also hold up when using this more confident set of regime shifts.**

**Response:** In response to this comment, we reproduced all major figures by masking the expression regime shifts in less-confident branches (bootstrap support < 99). The figures are included in Supplementary Data.

**Change:** Especially, the brain–testis–ovary and kidney–liver modules in S branches and the testis–ovary connection in D and R branches were always reproduced in the analyses with the above thresholds in combinations with the two expression metrics (SVA-log-TMM-FPKM and SVA-log-TPM; Fig. S11). The analysis of shifts in high-support branches also reproduced the other main results in this paper (Supplementary Data), demonstrating the robustness of our conclusion.

(There are many more figures in Supplementary Data, but below we will show those corresponding to the main figures.)

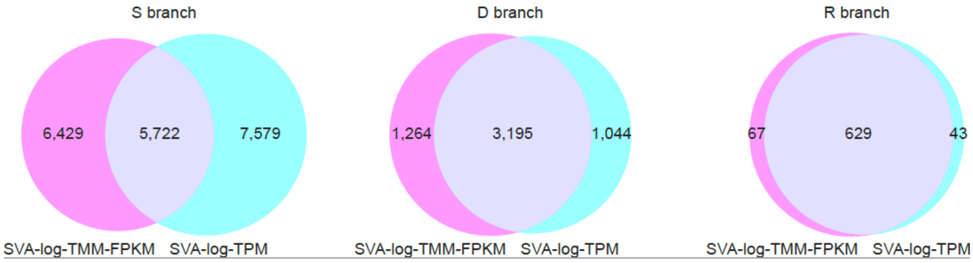


**Analysis of expression regime shifts in branches with >99 bootstrap supports, corresponding to Fig. 2C**

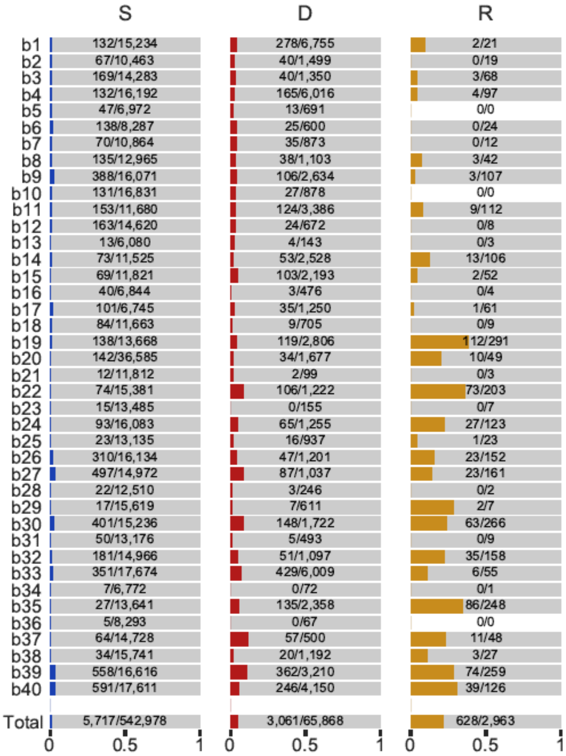


**Analysis of expression regime shifts in branches with >99 bootstrap supports, corresponding to Fig. 3B**

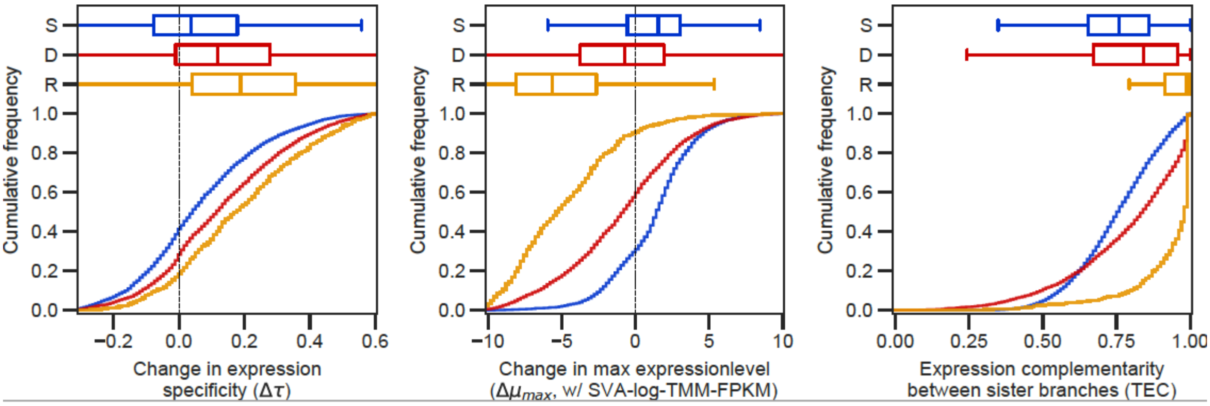


**Analysis of expression regime shifts in branches with >99 bootstrap supports, corresponding to Fig. 3D-F**

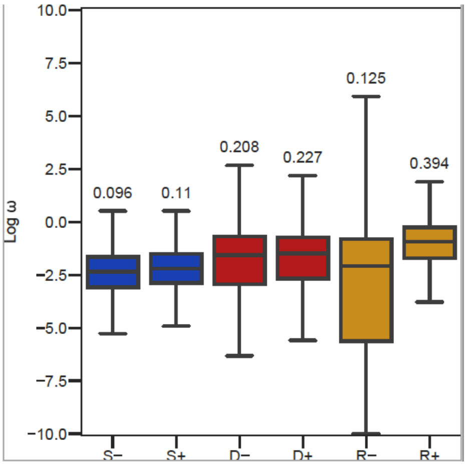

**Analysis of expression regime shifts in branches with >99 bootstrap supports, corresponding to Fig. 4A**

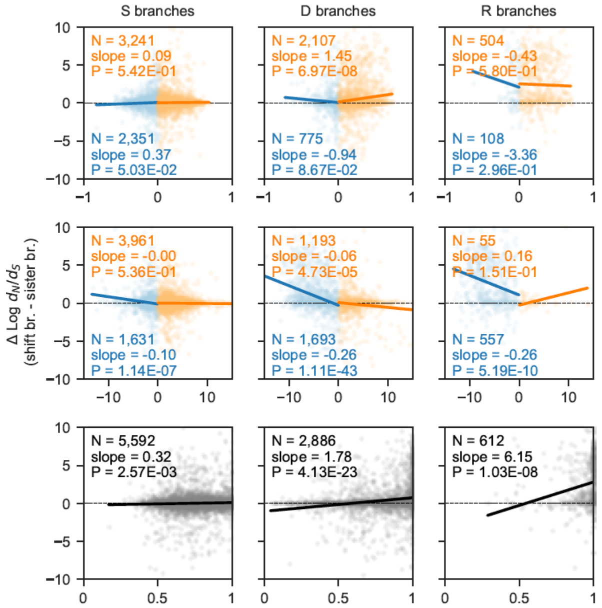


**Analysis of expression regime shifts in branches with >99 bootstrap supports, corresponding to Fig. 4B**

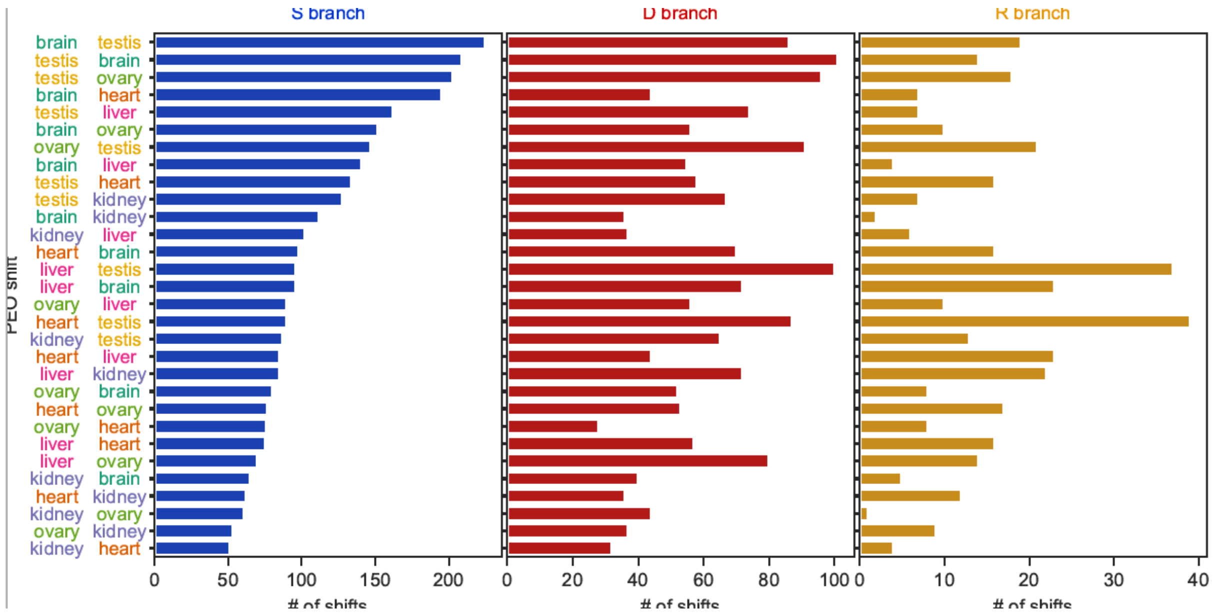


**Analysis of expression regime shifts in branches with >99 bootstrap supports, corresponding to Fig. 5A**

**Minor comments:**

**1) I am confused by the use of both TPM and FPKM throughout this paper. TPM is a more mathematically correct unit of RNA abundance and is preferred (https://pubmed.ncbi.nlm.nih.gov/22872506/). Furthermore, presenting both sets of data is confusing and renders the paper difficult to read. I would advocate to presenting only TPM results throughout the paper. Furthermore, the authors say that TMM normalization is not suitable for TPM but I'm unclear as to why this is? In either case, I don't know if using TMM normalized TPM would change the results in any major way, but they may want to check the normalization factors when using TPM to confirm this is true.**

**Response:** TPM is an estimated relative abundance that must, by definition, sum to $10^6$. If the TMM normalization is applied, the total is no longer $10^6$. This is why TMM is not applicable to relative abundance estimates (incl. TPM) but compatible with absolute abundance estimates (incl. FPKM) in which the total count has no hard constraint. We are aware that there is literature where TMM is operationally applied to TPM, but for the above reason, we decided not to do so. Because phylogenetic comparative methods we used in this paper have been designed and tested mainly with absolute trait values, rather than relative values, we prefer to present TMM-FPKM in the main text, while keeping all TPM-based analysis in the supplement for readers to check our methodological robustness in terms of absolute/relative estimates. In response to this comment, we explained the above reason in the main text.

**Change:** Because the TMM normalization destroys the estimated relative abundance of TPM, in which, by definition, the total counts must be $10^6$, this scaling method was applied only to FPKM values, but not to TPM values.

**2) Figure 1F seems unnecessary and a statistical truism - variance around the mean will necessarily decrease as sample size increases.**

**Response:** We agree with the notion of the relationship between mean and variance. Thanks to this comment, we noticed that the important aspect of this data is the relatively constant expression levels among organs. We deleted the original claim and emphasized this aspect in the new version of the manuscript.

**Change:** This idea is supported by subsampling analysis on a housekeeping gene GAPDH (glyceraldehyde-3-phosphate dehydrogenase), where, as more data are used, estimated expression levels in different organs tend to quickly converge to a similar range of values (Fig. 1F, ca. 15 SVA-log-TMM-FPKM; Fig. S4L, ca. 11.5 SVA-log-TPM).

**3) Line 377 - "Although the adjusted P value was not statistically significant, it is noteworthy that the top-ranked term for the brain–testis connection was "Endocrine and other factor-regulated calcium reabsorption" annotated to four genes including GNAQ, which has been implicated to tumor formation in neuronal tissues. " - what is the adjusted p-value here? If very high (>0.3), I do not think this result should be reported.**

**Response:** In response to this comment, we added the P value in the main text.

**Change:** The genes descended from the testis–ovary PEO shifts enriched only one KEGG pathway term "Cell cycle" (Table S6; adjusted P value < 0.05, Fisher's exact test with the Benjamini–Hochberg correction), likely reflecting their function in meiosis. Although the

adjusted *P* value was not statistically significant, it is noteworthy that the top-ranked term for the brain–testis connection was “Endocrine and other factor-regulated calcium reabsorption” (unadjusted *P* value = $1.66\times10^{-3}$; adjusted *P* value = 0.26) annotated to four genes including GNAQ, which has been implicated to tumor formation in neuronal tissues [52,53].

REVIEWERS' COMMENTS:

Reviewer #3 (Remarks to the Author):

My points from the previous round of review have now been satisfactorily corrected.

We thank the reviewers for their thorough and thoughtful reviews. A point-by-point response is provided below. We believe this has resulted in an improved manuscript that is appropriate for a *Nature Communications* audience.

## Reviewer #3

**My points from the previous round of review have now been satisfactorily corrected.**

**Response:** We thank the reviewer for their kind comments.
